# Nuclear MYH9-induced CTNNB1 transcription, targeted by staurosporin, promotes gastric cancer cell anoikis resistance and metastasis

**DOI:** 10.7150/thno.46001

**Published:** 2020-06-12

**Authors:** Gengtai Ye, Qingbin Yang, Xuetao Lei, Xianjun Zhu, Fengping Li, Jiayong He, Hao Chen, Ruoyu Ling, Haisheng Zhang, Tian Lin, Zhiping Liang, Yanrui Liang, Haipeng Huang, Weihong Guo, Haijun Deng, Hao Liu, Yanfeng Hu, Jiang Yu, Guoxin Li

**Affiliations:** 1 Department of General Surgery, Nanfang Hospital, Southern Medical University, Guangdong Provincial Engineering Technology Research Center of Minimally Invasive Surgery, Guangzhou, Guangdong 510515, China.; 2 Guangdong Provincial Key Laboratory of Precision Medicine for Gastrointestinal Tumor, Guangzhou, Guangdong 510515, China.; 3 Division of Molecular and Cellular Oncology, Dana-Farber Cancer Institute, Harvard Medical School, Boston, MA, USA.; 4 Present address: Department of General Surgery, Panyu Central Hospital, Guangzhou, Guangdong 511400, China.

**Keywords:** gastric cancer, MYH9, CTNNB1, anoikis resistance, metastasis

## Abstract

**Rationale:** Peritoneal metastasis predicts poor prognosis of gastric cancer (GC) patients, and the underlying mechanisms are poorly understood.

**Methods:** The 2-DIGE, MALDI-TOF/TOF MS and single-cell transcriptome were used to detect differentially expressed proteins among normal gastric mucosa, primary GC and peritoneal metastatic tissues. Lentiviruses carrying shRNA and transcription activator-like effector nuclease technology were used to knock down myosin heavy chain 9 (MYH9) expression in GC cell lines. Immunofluorescence, immune transmission electron microscopy, chromatin fractionation, co-immunoprecipitation, and assays for chromatin immunoprecipitation, dual luciferase reporter, agarose-oligonucleotide pull-down, flow cytometry and cell anoikis were performed to uncover nuclear MYH9-induced β-catenin (*CTNNB1*) transcription *in vitro*. Nude mice and conditional transgenic mice were used to investigate the findings *in vivo*.

**Results:** We observed that MYH9 was upregulated in metastatic GC tissues and was associated with a poor prognosis of GC patients. Mechanistically, we confirmed that MYH9 was mainly localized in the GC cell nuclei by four potential nuclear localization signals. Nuclear MYH9 bound to the *CTNNB1* promoter through its DNA-binding domain, and interacted with myosin light chain 9, β-actin and RNA polymerase II to promote *CTNNB1* transcription, which conferred resistance to anoikis in GC cells* in vitro* and *in vivo*. Staurosporine reduced nuclear MYH9 S1943 phosphorylation to inhibit *CTNNB1* transcription, Wnt/β-catenin signaling activation and GC progression in both orthotropic xenograft GC nude mouse and transgenic GC mouse models.

**Conclusion:** This study identified that nuclear MYH9-induced CTNNB1 expression promotes GC metastasis, which could be inhibited by staurosporine, indicating a novel therapy for GC peritoneal metastasis.

## Introduction

Although decade-long advancements in gastric cancer (GC) research have significantly improved surgical practices 1, 2 and comprehensive GC treatment regimens 3, GC still ranks as the fifth most frequently diagnosed cancer and the third leading cause of cancer-related deaths, responsible for over 1,000,000 new cases and causing 783,000 deaths worldwide in 2018 4. GC is a histologically and etiologically heterogeneous disease with marked phenotypic diversity and various molecular subtypes 5, 6. Established genetic and epigenetic alterations include gene amplifications (e.g. *JAK2*, *PD-L1* and *PD-L2*), gene mutations (e.g. *CDH1*, *RHOA* and *ARID1A*), DNA methylation (e.g. *TFF2*, *H19* and *MFAP2*), and oncogenic pathway activation (e.g. Wnt/β-catenin, proliferation/stem cell, Hippo and NF-κB pathways) 7-9. Based on the well-established “seed-and-soil” hypothesis 10, cancer cell-intrinsic determinants 11 and tumor cell-stromal cell interactions 12 in cancer cell metastasis have been studied extensively. However, the molecular mechanisms underlying GC dissemination, especially peritoneal metastasis, remain poorly defined. To identify candidate driver proteins that contribute to GC peritoneal metastasis, we detected differentially expressed proteins (DEPs) using two-dimensional fluorescence differential gel electrophoresis (2D-DIGE) and matrix-assisted laser desorption time-of-flight mass spectrometry (MALDI-TOF/TOF MS). We found that MYH9 expression was significantly higher in peritoneal metastases than in primary tumors and normal mucosae.

MYH9, also known as myosin IIa or non-muscle myosin heavy chain 9 (NMMHC-IIA), is a 226 kDa subunit of the class II conventional myosin, which exists as a hexameric enzyme composed of two heavy chains and two pairs of light chains 13. It is well known that MYH9 interacts with actins in the cytoplasm to take part in different cellular processes such as cell motility, cell migration and cell adhesion 13. Defects of MYH9 in humans lead to MYH9-related disease, which is an autosomal dominant thrombocytopenia with giant platelets 14. Ablation of the MYH9 gene results in lethality of mouse embryos within stage E7.5 due to defects in cell adhesion and the visceral endoderm 15, which suggests that MYH9 is involved in a number of irreplaceable roles that are not fully elucidated. In addition, the functions of MYH9 in cancer are controversial 16-19, especially in GC 16, 17. As an effector molecule in the Rho-Rho-associated protein kinase (ROCK) pathway, MYH9 was shown to promote the invasion and metastasis of GC cells 16, 20. However, MYH9 knockdown was also reported to induce intracytoplasmic mucin and cellular elongation in mucinous gastric carcinoma (MGC) 17, which has garnered further interest in MYH9.

In this study, we identified epithelium-derived cells in GC tissues and confirmed that MYH9 was upregulated in metastatic GC cells by single-cell transcriptome analysis, which supported our 2D-DIGE data. We found MYH9 was mainly located in the GC cell nuclei, and presented the first evidence showing nuclear MYH9, with special nuclear localization signals and a DNA-binding domain, directly bound to the *CTNNB1* promoter to induce β-catenin transcription and increase activation of the canonical Wnt/β-catenin signaling pathway, which equipped GC cells with anoikis resistance and promoted GC metastasis. We also confirmed that staurosporine decreased nuclear MYH9 phosphorylation at S1943 to inhibit the MYH9-CTNNB1 axis-mediated canonical Wnt/β-catenin signaling activation in cell lines and in the GC mouse models (orthotropic xenograft GC mouse models and conditional transgenic GC mouse models).

## Results

### MYH9 expression is associated with a poor GC prognosis and an increase in CTNNB1 transcription

**Figure 1:**
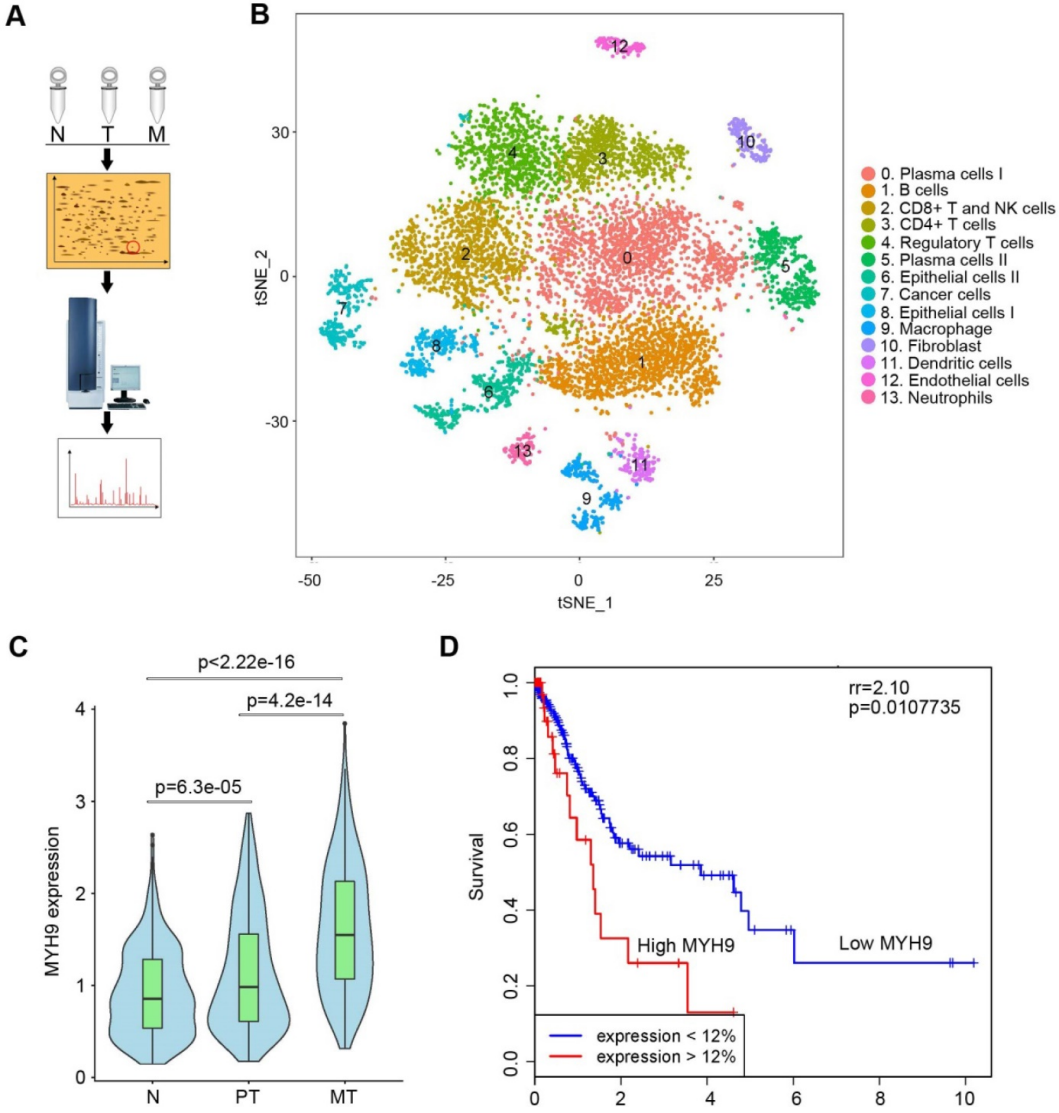
** MYH9 was upregulated in metastatic GC tissues and associated with poor survival of GC patients.** (A) Illustration of 2D-DIGE and MALDI-TOF/TOF MS analyses for GC tissues. N, normal gastric mucosae; T, primary GC tissues; M, peritoneal metastasis tissues. (B) t-distributed stochastic neighbor embedding (t-SNE) plot of 10,189 single cells from two advanced GC patients. The tissues included normal gastric epithelium (N), primary tumor (PT) and peritoneal metastasis (MT). Clusters were assigned to indicated cell types by differentially expressed genes (see also Figure S3 and Table S7). (C) The level of *MYH9* mRNA in epithelium-derived cells (Cluster 6, 7 and 8) was analyzed using the single-cell transcriptome data (Kruskal-Wallis, *p* < 2.2e-16). (D) The Kaplan-Meier survival analysis of overall survival in TCGA GC data based on MYH9 expression. The level of *MYH9* mRNA was divided into low (<12th percentile) and high (>12th percentile) groups for analysis.

To search driver proteins that contribute to GC peritoneal metastasis, we analyzed DEPs among normal gastric mucosa, primary GC tissues and peritoneal metastases using 2D-DIGE and MALDI-TOF/TOF MS (Figure 1A, S1A and S1B; Tables S1). We identified 35 DEPs (Table S2) and confirmed MYH9 was significantly upregulated in metastatic GC tissues by western blot (Figure S1C) and qPCR (Figure S2A; Table S3). This was further supported by the data from The Cancer Genome Atlas (TCGA; Figure S2B) and Gene Expression across Normal and Tumor tissues (GENT; Figure S2C). Since single-cell RNA sequencing (scRNA-seq) offered a potential solution for dissecting the tissue heterogeneity, we performed scRNA-seq on tissues from two advanced GC patients, including primary GC tissues, peritoneal metastases and corresponding normal gastric mucosae (Table S4). After analysis of all 10,189 cells, we classified these cells into cell type groups using graph-based clustering on the informative principle components, which identified cell clusters that could be assigned to known cell lineages by marker genes (Figure 1B, S3A, S3B and Table S4). We found that the level of MYH9 mRNA in epithelium-derived cells from peritoneal metastases was the highest, followed by that of epithelium-derived cells from primary GC tissues and normal gastric mucosa (Figure 1C). Furthermore, we found that *MYH9* mRNA was inversely associated with survival of GC patients from TCGA (Figure 1D) and KMplot (http://kmplot.com) datasets (Figure S4A-D), and positively associated with the pT stage of TCGA GC patients (Figure S4E).

**Figure 2:**
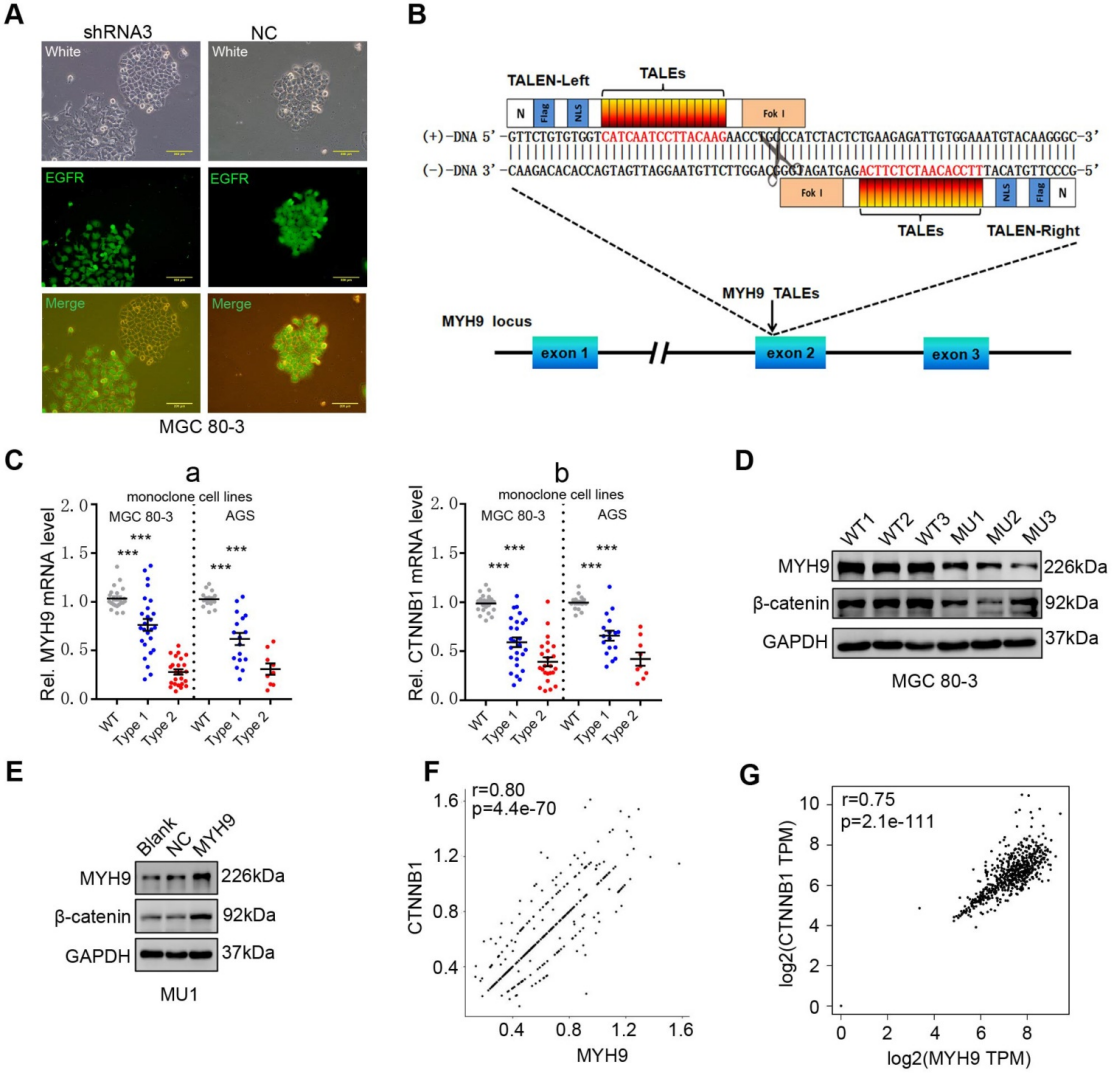
** MYH9 promotes CTNNB1 expression in GC cells.** (A) Morphology of MYH9 shRNA3-transfected or NC-transfected MGC 80-3 cells (green) were observed under a fluorescence microscope. The nude cells (no green) served as internal controls. (B) Schematic illustration of a pair of TALENs (L2/R3) binding to *MYH9* exon 2. (C) Monoclonal MGC 80-3 and AGS sublines were generated by transfection of L2 and R3 plasmids and then subjected to qPCR analysis of *MYH9* (a) and *CTNNB1* (b) mRNA. WT, wild type monoclonal cells; Type 1, monoclonal cells whose single chain of MYH9 DNA was edited; Type 2, monoclonal cells whose double chains of MYH9 DNA were edited. (D) Three MGC 80-3 monoclonal cells with MYH9 knocked down were subjected to western blot analysis. Three wild-type monoclonal cells were used as controls. (E) Monoclonal MGC-80-3 cells (MU1) was transfected with MYH9 plasmid and subjected to western blot analysis. (F) The correlation between *MYH9* and *CTNNB1* mRNA expression in gastric epithelium-derived cells (including both gastric epithelial and cancer cells) was analyzed using the single-cell transcriptome data. (G) The correlation between *MYH9* and *CTNNB1* mRNA expression in GC tissues and normal gastric mucosae was analyzed using the GEPIA dataset.

We then constructed GC cell lines (MGC 80-3 and AGS) with stable MYH9 knockdown by transfecting MYH9 shRNAs (Table S5). Cells transfected with shRNA3 were chosen for this study (details in Figure S5A-C). Using fluorescence microscopy, we found that MYH9 shRNA3-infected cells had loose intercellular connections (Figure 2A) and a morphology similar to cells undergoing an epithelial-mesenchymal transition 21, 22, which suggests that MYH9 may be a tumor suppressor. However, MYH9 has been confirmed to be an oncogene and promote GC cell metastasis in our previous study 16. To clarify this contradiction, we performed western blotting and the results showed no significant association of MYH9 expression with levels of vimentin, E-cadherin, or Snail in MYH9 shRNA-infected cells (Figure S5D, S5E). Unexpectedly, we found that the levels of β-catenin protein (Figure S5D, S5E and S6) and mRNA (Figure S5F) were significantly downregulated in these MYH9 knockdown cells. Our rescue experiments revealed that levels of both *MYH9* and *CTNNB1* mRNA were re-expressed (Figure S5F), suggesting that MYH9 promoted *CTNNB1* transcription. To further confirm this finding, we used transcription activator-like effector nuclease (TALEN) technology to knock down the *MYH9* gene in GC cells (details in Figure 2B, S7A-E; Table S6, S7). The mRNA level (Figure 2C) and protein level of β-catenin (Figure 2D) in GC monoclonal sublines (Table S7) with MYH9 knockdown was significantly reduced, whereas overexpression of MYH9 in one of these monoclonal sublines (MU1) restored MYH9 and β-catenin protein expression (Figure 2E). Indeed, our scRNA-seq analysis showed that *MYH9* and *CTNNB1* mRNA levels in epithelium-derived cells had a significantly positive association (r=0.80; *p*=4.4e-70; Figure 2F), consistent with the combined analysis of the TCGA GC dataset and GTEx stomach data (Figure 2G).

### Nuclear MYH9 interacts with myosin light chain 9, β-actin and RNA polymerase II to promote CTNNB1 transcription

**Figure 3:**
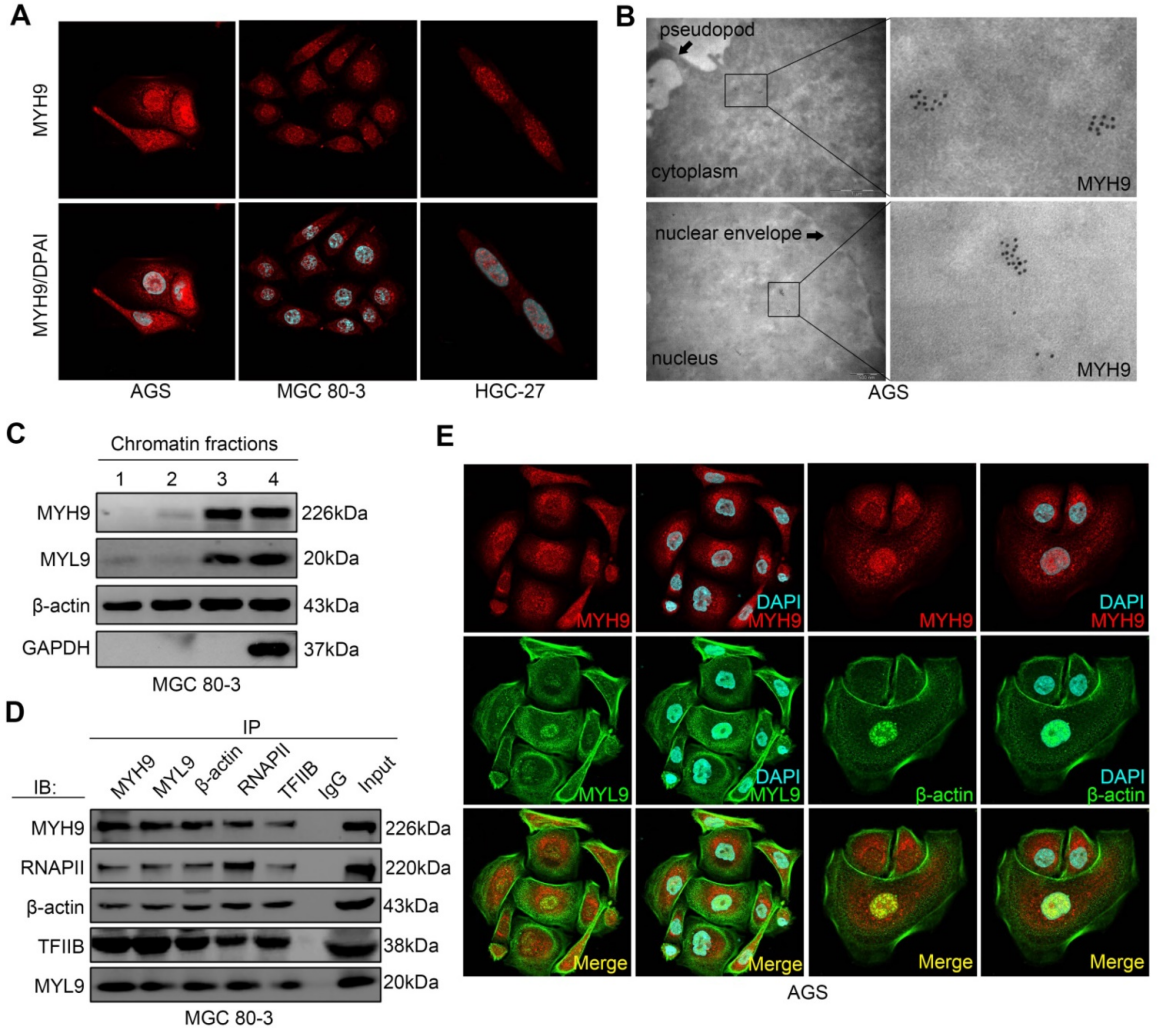
** MYH9 interacts with MYL9, β-actin, RNAPII and TFIIB in the nucleus of GC cells.** (A) AGS, MGC 80-3 and HGC-27 cells were grown and subjected to immunofluorescence staining (MYH9, red; DAPI, blue) and confocal microscopy. (B) AGS cells were subjected to immune transmission electron microscopic analysis of MYH9 protein (black dots). (C) MGC 80-3 cells were subjected to chromatin fractioning and western blot. 1, 0.4 M chromatin fraction; 2, 0.8 M chromatin fraction; 3, chromatin residual pellet. 4, control. (D) MGC 80-3 cells were subjected to nuclear protein extraction, IP and western blot. Rabbit IgG was used as the negative control. (E) AGS cells were subjected to immunofluorescence staining (MYH9, MYL9 and β-actin proteins) and confocal microscopy.

To explore the mechanism by which MYH9 increased *CTNNB1* transcription, we first assessed MYH9 protein localization in GC cell lines, and found that MYH9 accumulated abundantly in the GC cell nuclei by confocal microscopy (Figure 3A), transmission electron microscopy (Figure 3B) and western blot (Figure S8A, S8B). This result is consistent with the predicted outcome from PSORT II (http://www.genscript.com/cgi-bin/tools/psort2.pl), a protein nuclear localization prediction website.

To assess whether MYH9 regulated gene transcription 23, 24, we performed chromatin fractionation followed by western blot. Our data showed the presence of β-actin in all three chromatin fractions, whereas MYH9 and myosin light chain 9 (MYL9) proteins were only detected in the residual pellet and controls (Figure 3C), indicating that MYH9 and MYL9 might interact with chromatin. Moreover, we found that the MYH9 antibody pulled down MYL9, β-actin, RNA polymerase II (RNAP II) and transcription factor II B (TFIIB; Figure 3D) by co-immunoprecipitation. This finding confirms the role of MYH9 as part of the transcriptional machinery in GC cells. In addition, using immunofluorescence we found that MYH9 was co-localized with MYL9 and β-actin in the nucleus of AGS cells (Figure 3E) and GC tissues (Figure S8C, S8D).

**Figure 4:**
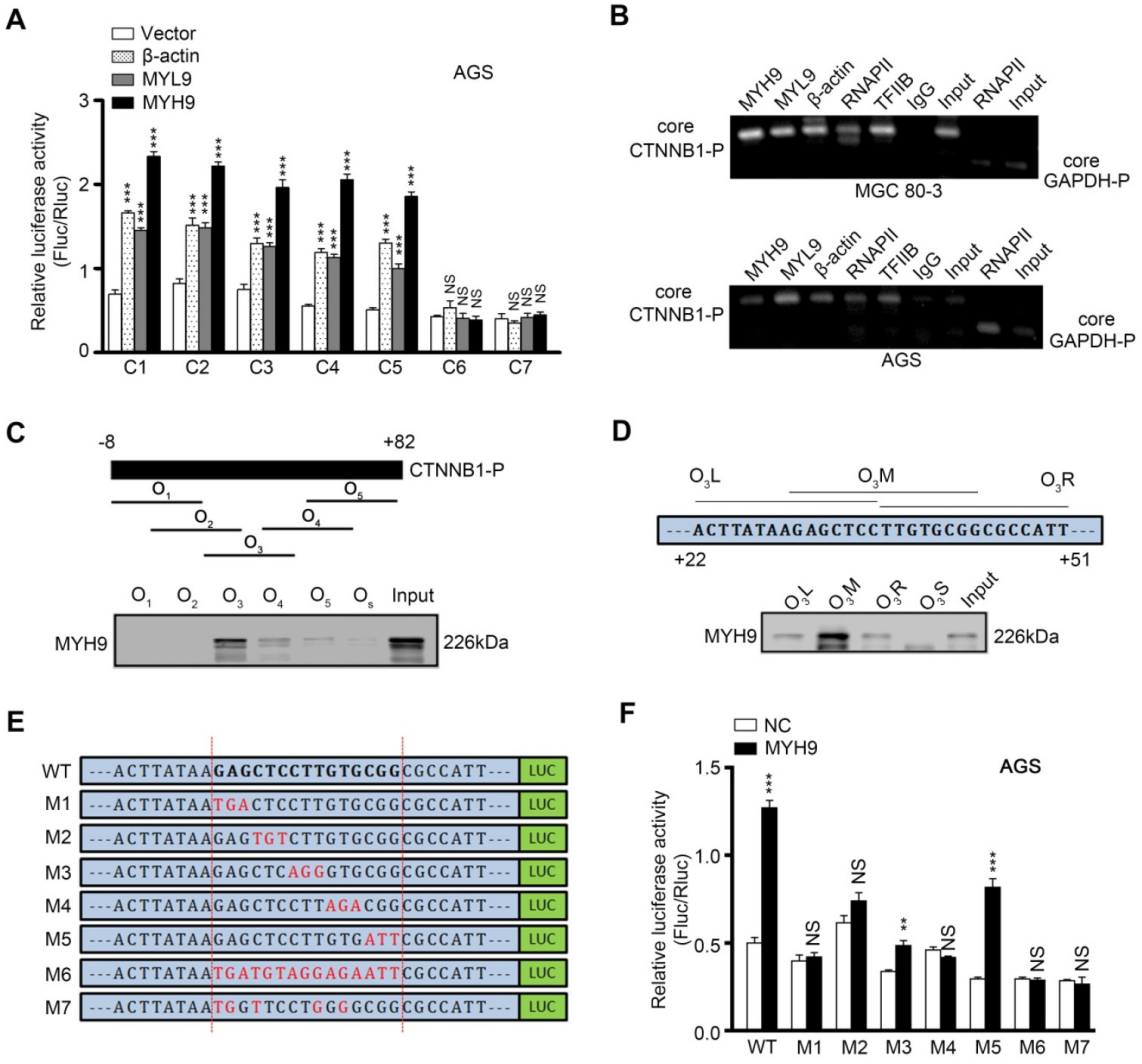
** Nuclear MYH9 binds to the *CTNNB1* promoter with special sequences to promote *CTNNB1* transcription *in vitro*.** AGS cells were co-transfected with a Firefly luciferase reporter plasmid, a Renilla luciferase reporter plasmid and a gene overexpression plasmid for 24 h and then subjected to luciferase reporter assay. The Firefly luciferase reporter plasmid contains different lengths of the *CTNNB1* promoter cDNA (C1-7). The gene overexpression plasmids contain MYH9, MYL9 or β-actin cDNA. (B) MGC 80-3 and AGS cells were subjected to ChIP analysis. MYH9, MYL9, β-actin, RNAPII and TFIIB antibodies were used. GAPDH primers served as a positive control, while IgG served as a negative control. Nuclear extracts of AGS cells were prepared and pulled down with biotinylated oligonucleotides (C, O1-5; D, O3L, O3M, O3R and O3S) targeting the *CTNNB1* promoter (the top graph), which were then subjected to western blot analysis of MYH9 protein. Input served as a positive control. O3S was a scramble biotinylated oligonucleotide that served as a negative control. (E) Schematic representation of mutated *CTNNB1* promoters that were cloned into the upstream of the luciferase reporter (M1-7). (F) MYH9-overexpressed (LV-MYH9) AGS cells were co-transfected with a Firefly luciferase reporter plasmid (M1-7) and a Renilla luciferase reporter plasmid for 24 h, and then subjected to luciferase reporter assay. WT, the C3 plasmid, served as a positive control.

To investigate whether MYH9 directly bound to the *CTNNB1* promoter, we first generated seven luciferase reporter vectors (C1-7) containing various lengths of the *CTNNB1* promoter (Figure S9A), and co-transfected one of these plasmids with MYH9, MYL9 or β-actin plasmid into AGS (Figure 4A) and MGC 80-3 (Figure S9B) cells. The dual luciferase reporter assay showed that the construct with a 43 bp promoter region (between +3 bp and +45 bp) was mostly responsible for *CTNNB1* transcription (Figure 4A, S9A and S9B). Our chromatin immunoprecipitation (ChIP) assay also showed that MYH9, along with MYL9, β-actin, RNAPII and TFIIB, could bind to the +3 to +270 bp region of CTNNB1 promoter (Figure 4B). Furthermore, we identified the MYH9-binding motif on the *CTNNB1* promoter by using a 91 bp (between -8 bp and +82 bp) *CTNNB1* promoter for agarose-oligonucleotide pull-down assays in the nuclear extracts of GC cells. Among the five overlapping oligonucleotides (Figure 4C) for the first-round screening, oligonucleotide O3 pulled down the MYH9 protein (Figure 4C; Table S8), indicating that the region (+22 to +51 bp) targeted by O3 contains the MYH9-binding motif. Through second-round screening with three smaller overlapping oligonucleotides, we found oligonucleotide O3M, targeted to the +30 to +44 bp region of *CTNNB1* promoter, pulled down the MYH9 protein (Figure 4D). Moreover, we constructed six three-point mutation plasmids (mutated within the +30 to +44 bp region of *CTNNB1* promoter) based on the firefly fluorescent plasmid (Figure 4E; pGL3-CTNNB1P; +3 to +270 bp). The dual luciferase reporter assay showed that four plasmids (M1, M2, M4 and M6) failed to induce luciferase activity in both AGS and MGC 80-3 cells (Figure 4F, S9C), suggesting that these mutated points were critical for MYH9-induced *CTNNB1* transcription.

### Nuclear MYH9 contains one DNA-binding domain for regulating CTNNB1 transcription and four nuclear localization signals

**Figure 5:**
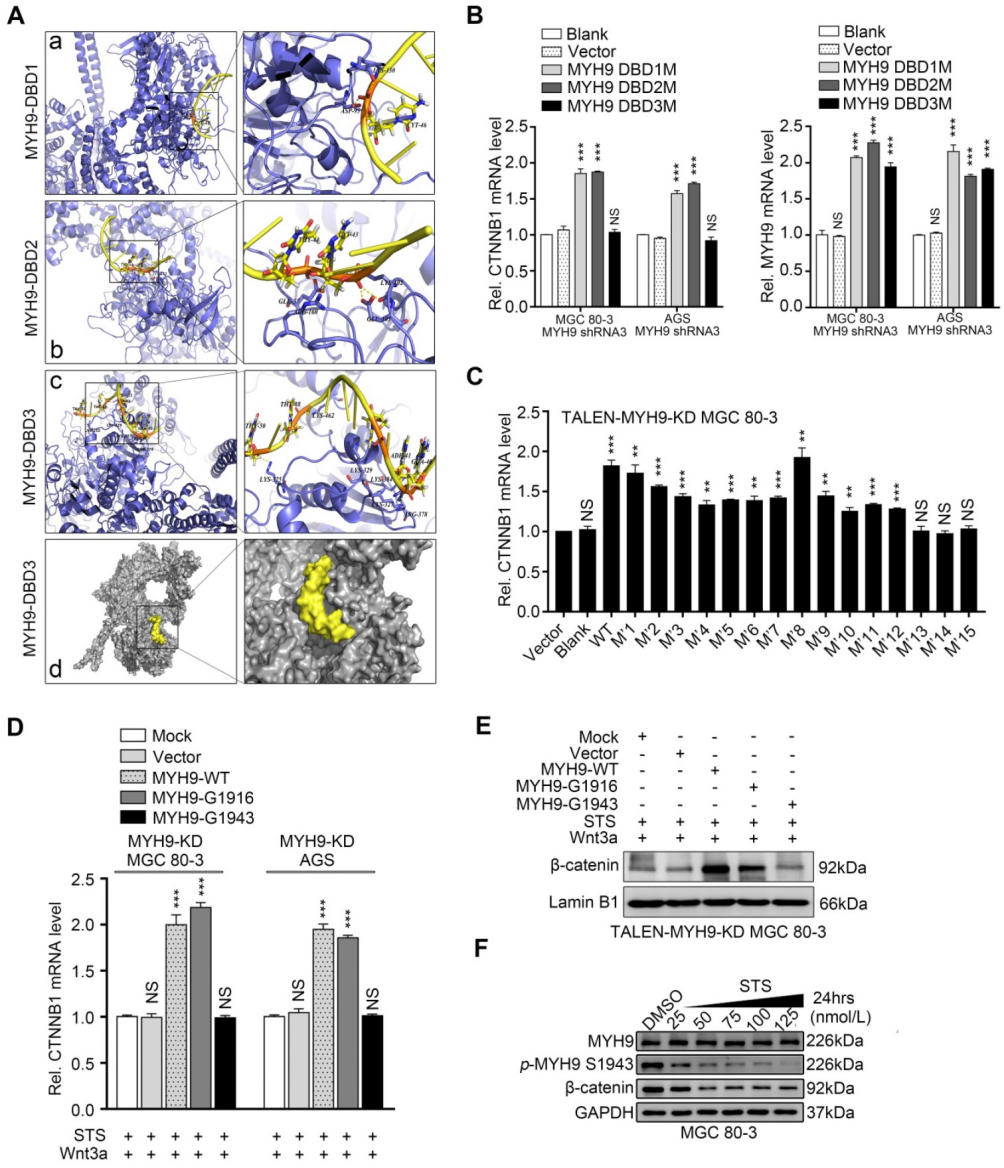
** STS inhibits nuclear MYH9 phosphorylation at S1943 to decrease CTNNB1 expression.** (A) Structural illustration of *CTNNB1* promoter (*CTNNB1*-P) and MYH9 protein. a-c. Ribbon diagram of three potential interactions between CTNNB1-P and MYH9 DBDs using Pymol software. d. Surface representation of the interaction (grey, MYH9; yellow, *CTNNB1*-P) confirmed by our research. (B) MGC 80-3 and AGS cells were transiently transfected with three different mutated DNA binding sites (DBS1M, DBS2M or DBS3M) after knockdown of MYH9 expression using MYH9 shRNA3, and then subjected to qPCR analysis of *CTNNB1* and *MYH9* mRNAs. *GAPDH* mRNA served as an internal control. (C) MGC 80-3 cells with MYH9 knockdown (MYH9-KD) were transiently transfected with different NLS mutation plasmids (M1-15) and then subjected to qPCR analysis of *CTNNB1* mRNA. (D) AGS and MGC 80-3 cells with MYH9 knockdown (MYH9-KD) were transfected with different MYH9 mutation plasmids (G1916 or G1943) for 12 h, then treated with STS (100 nmol/L) for 24 h. The cells were treated with Wnt3a (20 ng/mL) for 8 h before being harvested for RNA cleavage. The expression of *CTNNB1* mRNA was detected by qPCR. (E) MGC 80-3 cells with MYH9 knockdown (TALEN-MYH9-KD) were transfected with MYH9 mutation plasmids (G1916 or G1943), treated with STS (100 nmol/L) and Wnt3a (20 ng/mL), and then subjected to nuclear protein extraction and western blot analysis of β-catenin expression. (F) MGC 80-3 cells were treated with STS (25, 50, 75, 100 and 125 nmol/L) for 24 h and then subjected to western blot analysis of *p*-MYH9 (S1943) and β-catenin levels.

We then searched the PSORT II website and found three potential MYH9 DBDs (i.e. MYH9-DBD1 [5'-LEEEQIILEDQNCKLAKEKKLL-3'], MYH9-DBD2 [5'-LEKTRRKLEGDSTDLSDQIAEL-3'] and MYH9-DBD3 [5'-LEKAKQTLENERGELANEVKVL-3']). Following MYH9 3D structure modeling and DNA-protein docking analysis, we used Pymol software to analyze the interaction between *CTNNB1*-P and MYH9 DBDs (Figure 5A, Table S9, details in supplementary methods). Our results showed that the binding energy between MYH9 DBDs and *CTNNB1*-P was -24.7498 kJ/mol for DBD1, -120.947 kJ/mol for DBD2 and -781.88 kJ/mol for DBD3. Furthermore, transfection with MYH9 plasmids carrying the DBD1 or DBD2 mutation (DBD1M or DBD2M) but not the DBD3 mutation (DBD3M) upregulated the level of *CTNNB1* mRNA in MYH9 shRNA3-infected MGC 80-3 and AGS cells (Figure 5B; Table S10). We also mutated the firefly fluorescent plasmid (Figure 4E, 4F and S9C) as M7 plasmid based on the docking result (Table S9) for the luciferase reporter assay, and found that the M7 mutation plasmid failed to change the luciferase activity in both AGS and MGC 80-3 cells (Figure 4F and S9C) further suggesting that the MYH9 DBD3 was the core domain for MYH9 binding to the *CTNNB1* promoter.

We further identified four putative MYH9 nuclear localization signals (NLSs) using the PSORT II prediction program, namely three mono-partite NLSs (KKRH, HKRK and KRKK) and one bipartite NLS (5'-RREEKQRQELEKTRRKL-3'; Table S11). Subsequently, we selectively mutated each of the NLSs by replacing lysine (K) or arginine (R) residues with alanines (A) to obtain four MYH9 NLS mutation plasmids (Table S11). After transfecting each of these plasmids into MGC 80-3 KD1 cells, we detected green florescent protein (GFP) expression using an immunofluorescence assay. The results showed that in all of the tested cells MYH9 was predominantly localized in the nucleus when the cells were transfected with NLS1M or NLS2M, while MYH9 in 91.67% (55/60) and 95% (57/60) of the tested cells was predominantly localized in the nucleus when the cells were transfected with NLS3M and NLS4M, respectively (Figure S10; Table S12). These results suggest that NLS3 and NLS4 are more important in the cytoplasmic-to-nuclear translocation of MYH9, and any single putative NLS mutation was not sufficient to prevent this process. Thus, we constructed 11 plasmids with combined mutations of these four NLSs and transfected them into MGC 80-3 KD1 cells. The results showed that four combinations of MYH9 NLS mutations (i.e. M'10, M'13, M'14 and M'15; Table S12) could significantly decrease MYH9 nuclear localization, especially M'13 and M'15 (Figure S10 and Table S12). Moreover, we found the level of *MYH9* mRNA was upregulated in all MYH9-mutated plasmid-transfected cells (Figure S11), while the level of *CTNNB1* mRNA was only upregulated in cells transfected with 12 MYH9 mutated plasmids (M'1-12; Figure 5C), which suggests three combined mutations (M'13-15) could prevent MYH9 translocation into the nucleus to induce *CTNNB1* transcription. In addition, although the M'10 plasmid (NLS3M+4M) still induced MYH9 nuclear accumulation in 58.33% of the tested cells, it failed to significantly upregulate the level of *CTNNB1* mRNA, suggesting that the nuclear MYH9 accumulation of these tested cells was not sufficient, and that NLS1 and NLS2 also contributed to the nuclear accumulation of MYH9.

### STS downregulates CTNNB1 transcription by inhibition of nuclear MYH9 phosphorylation at S1943

To identify effective inhibitors of MYH9-mediated *CTNNB1* transcription in the nucleus, we treated GC cells with blebbistatin (type II non-myosin ATPase inhibitor), CX-4945 (the selective casein kinase 2 inhibitor), ML-7 (the myosin light chain kinase inhibitor), microcystin-LR (MCLR; the myosin light chain phosphatase), staurosporine (STS; the kinase inhibitor) or Y-27632 (ROCK inhibitor). These compounds have been reported to inhibit myosin-actin interactions (Figure S12) 13. After determining IC_50_ values for the various agents in GC cells (Figure S13), we found that only STS treatment significantly downregulated *CTNNB1* mRNA expression in both MGC 80-3 and AGS cells (Figure S14). Therefore, we selected STS (100 nmol/L for 24 h treatment) for further study.

STS was previously reported to suppress MYH9 phosphorylation at S1916 13. We hypothesized that STS could downregulate *CTNNB1* mRNA through suppression of MYH9 phosphorylation. Since five well-known phosphorylation sites are localized at the C-terminus of MYH9 13, we mutated these five phosphorylation sites (Table S10) and transfected the plasmids into GC cell lines with MYH9 knockdown (MYH9-KD cells). Our results showed that the S1943 mutation (G1943) failed to upregulate the level of *CTNNB1* mRNA in both cell lines, whereas the other four mutated plasmids were able to significantly upregulate the level of *CTNNB1* mRNA (Figure S15), suggesting that MYH9 S1943 phosphorylation, but not S1916 phosphorylation, might be important in the regulation of *CTNNB1* transcription in GC cells. Since β-catenin, encoded by the *CTNNB1* gene, was a transducer of the canonical Wnt signaling to the cell nucleus and its nuclear accumulation could be activated by Wnt3a, we treated GC cells with Wnt3a according to a previous study 25. It was observed that Wnt3a significantly upregulated the amount of nuclear β-catenin in AGS cells in a time-dependent manner, and 8 hours was the optimal drug stimulation time (Figure S16A). Furthermore, we transfected MYH9-mutated plasmids (G1916 or G1943) into MYH9-KD GC cells, and treated them with STS (100 nmol/L; 24 h) and Wnt3a (20 ng/mL; 8h). We found that G1916 overexpression, but not G1943 overexpression, significantly upregulated the level of *CTNNB1* mRNA (Figure 5D) and increased nuclear accumulation of β-catenin in TALEN-MYH9-KD GC cells (Figure 5E, S16B). This result further indicated the importance of MYH9 S1943 phosphorylation in *CTNNB1* transcription. For confirmation, we assessed the level of β-catenin in GC cells after STS treatment and found that STS significantly inhibited both MYH9 S1943 phosphorylation and β-catenin expression in a dose-dependent manner (Figure 5F, S16C). We further found that the S1943 plasmid, but not the G1943 plasmid, significantly increased the level of β-catenin in MGC 80-3 cells (Figure S16D). In addition, we found STS treatment (100 nmol/L) significantly downregulated both nuclear and cytoplasmic levels of *p*-MYH9 S1916 and *p*-MYH9 S1943 proteins, while CX-4945 treatment (5 μmol/L) only reduced the cytoplasmic level of *p*-MYH9 1943 proteins (Figure S16E), suggesting that STS might have better nuclear permeability, and supporting the fact that STS suppresses MYH9 S1943 phosphorylation in GC cells. It has been well established that MYH9 S1943 is phosphorylated by casein kinase II (CK-II), and CX-4945 is a selective ATP-competitive small molecule protein kinase CK-II inhibitor 13 (Figure S12). However, a previous study reported that STS could also inhibit CK-II in the rat brain 26 (Figure S12), which suggested that STS inhibited nuclear MYH9 S1943 phosphorylation by decreasing nuclear CK-II activity in GC cells.

### Nuclear MYH9-mediated CTNNB1 expression induces activation of canonical Wnt/β-catenin signaling and anoikis resistance in GC cells

**Figure 6:**
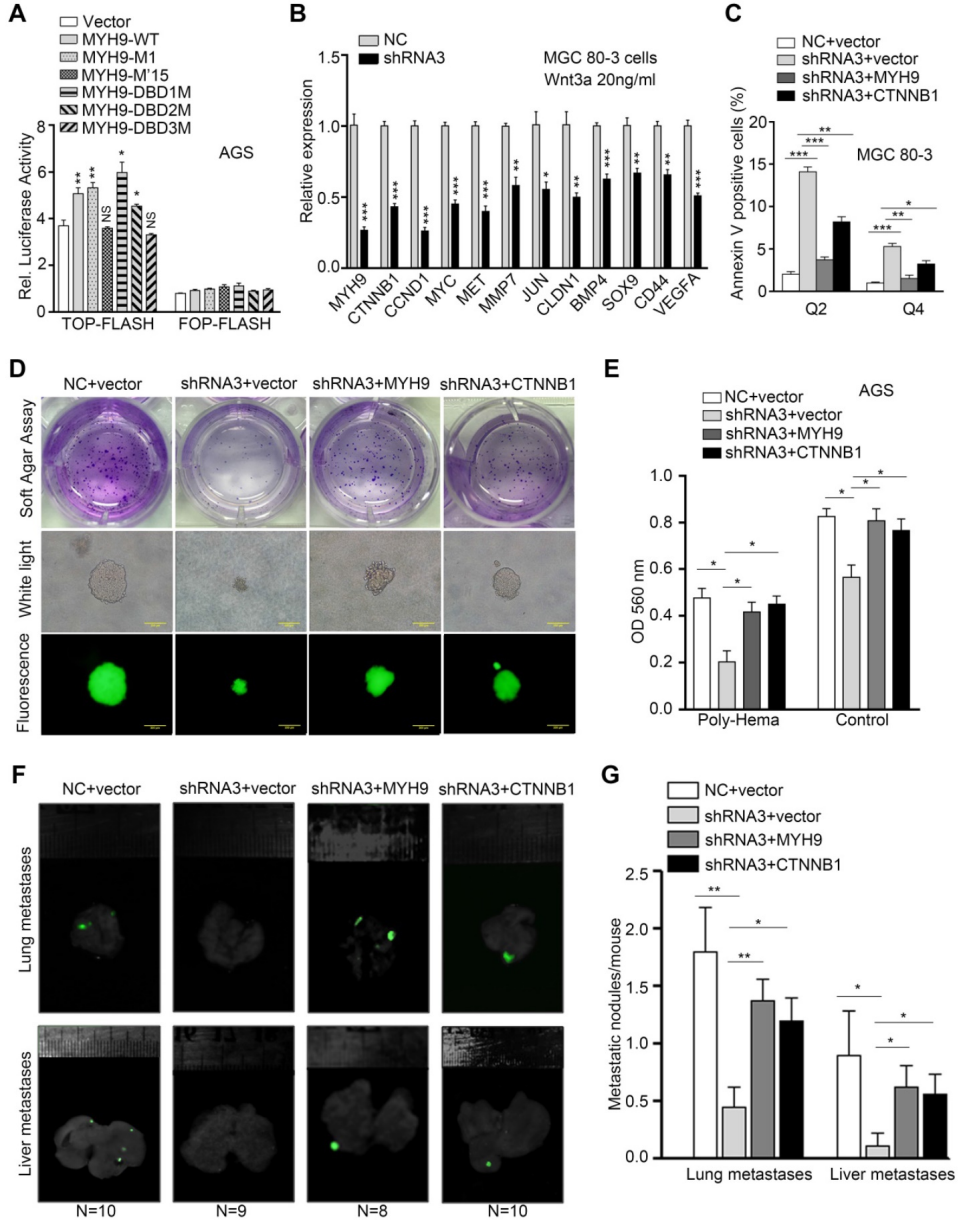
** MYH9-mediated *CTNNB1* transcription promotes anoikis resistance in GC cells by increasing the activation of Wnt/β-catenin signaling.** AGS cells were co-transfected with a MYH9 plasmid (wild type or mutated), a luciferase reporter plasmid (TOP-flash or FOP-flash) and a Renilla control plasmid for 16 h, and then subjected to luciferase reporter assay. The cells also treated with Wnt3a (20 ng/ml) for 8 h before the assay. (B) MGC 80-3 cells transfected with MYH9 shRNA3 were treated with Wnt3a (20 ng/mL) for 8 h, and then subjected to qPCR analysis of β-catenin-induced target genes. (C) MGC 80-3 cells were subjected to annexin-V and 7-AAD staining for flow cytometry analysis. (D) MGC 80-3 cells transfected with different plasmids (NC+vector, shRNA3+vector, shRNA3+MYH9 or shRNA3+CTNNB1) were seeded into 12-well plates and then subjected to a soft agar colony formation assay for 21 days. The colony formation was observed under naked eyes, the bright-field microscope (200x) and fluorescence microscope (200x; see also Figure S20A and Figure S20B). (E) AGS cells transfected with different plasmids (NC+vector, shRNA3+vector, shRNA3+MYH9 or shRNA3+CTNNB1) were seeded into a poly-hema pre-coated plate (or a control cell culture plate), grown for 24 h, then subjected to the MTT assay. (F) MGC 80-3 cells were injected into the mouse tail vein. Eight weeks after tumor cell injection, the mice were sacrificed and subjected to a multi-functional* in vivo* imaging system. (G) The number of lung and liver metastatic nodules per mouse was counted and analyzed.

Since the canonical Wnt/β-catenin signaling is one of the most activated signaling pathways in GC 27, we hypothesized that MYH9 induces β-catenin expression to increase activation of canonical Wnt signaling and improve GC cell survival. To test this hypothesis, we performed TOP/FOP-flash assays and found that MYH9 knockdown significantly inhibited the activity of the TOP-flash reporter in GC cells (Figure S17A), while MYH9 overexpression enhanced its activity, especially in Wnt3a-treated cells (Figure S17B). We also transfected MYH9 S1943-mutated plasmid (G1943) or positive control MYH9 S1943 plasmid (S1943) into AGS cells for assessment of TOP/FOP-flash activity. Our results show that MYH9 G1943 overexpression failed to induce activity of the TOP-flash reporter in AGS cells, even after Wnt3a treatment (20 ng/mL; Figure S17C). Furthermore, we transfected one of the mutation plasmids (NLS mutation plasmid, M'15; DBD mutation plasmids, DBD1-3M) into GC cells and found that both M'15 and DBD3M failed to increase the activation of the TOP-flash reporter in AGS cells (Figure 6A). In addition, we found MYH9 knockdown suppressed the mRNA levels of β-catenin-induced target genes in MGC 80-3 cells after Wnt3a treatment (20 ng/mL) (Figure 6B), while transfection with the MYH9 overexpression plasmid reversed these effects (Figure S17D). These findings were further supported by a Pearson's correlation analysis of MYH9 and β-catenin-induced target genes in epithelium-derived cells from scRNA-seq data (Figure S18). However, we also found that transfection with the MYH9 overexpression plasmid failed to upregulate vascular endothelial growth factor A (VEGFA; Figure S17D) and that the level of MYH9 mRNA was not associated with that of Jun mRNA (Figure S18), which support gastric cancer cell heterogeneity and suggest that VEGFA and Jun expression may be regulated by multiple factors in cell lines and GC tissues. Overall, these results support the conclusion that MYH9-mediated CTNNB1 expression increases canonical Wnt/β-catenin signaling activation in GC cells.

To determine if nuclear MYH9-associated CTNNB1 expression is involved in the biological functions of GC cells, we established four GC cell models transfected with negative control and vector (NC + vector), MYH9 shRNA3 and vector (shRNA3 + vector), MYH9 shRNA3 and MYH9 plasmid (shRNA3 + MYH9) or MYH9 shRNA3 and CTNNB1 plasmid (shRNA3 + CTNNB1). The results of the apoptosis assay revealed that MYH9 knockdown (MYH9 shRNA3 + vector) increased tumor cell apoptosis when compared with the control group (NC + vector), while MYH9 or CTNNB1 overexpression (MYH9 shRNA3 + MYH9 or MYH9 shRNA3 + CTNNB1) rescued tumor cell apoptosis (Figure 6C, S19A and S19C), suggesting that MYH9 knockdown-induced cell apoptosis is associated with a reduction of CTNNB1 expression. In addition, the cell cycle distribution assay revealed that MYH9 knockdown blocked the cell cycle at the G2-M phase (S19B and S19D). MYH9 overexpression, but not CTNNB1 overexpression, restored the G2-M arrest, which suggests that MYH9-associated CTNNB1 expression is not associated with cell cycle. Since β-catenin (CTNNB1) could trigger tumor cell anchorage-independent growth and inhibit cancer cell anoikis 28, a special type of apoptosis that occurs in GC cells disseminating into the abdominal cavity 29, we hypothesized that MYH9-mediated *CTNNB1* transcription promotes GC cell anoikis resistance. Indeed, our soft agar colony formation assay showed that the number of soft agar clones was significantly reduced and the clone diameter was also smaller in MYH9-knockdown MGC 80-3 cells (MYH9 shRNA3 + vector) when compared with the control cells (NC + vector). Similarly, both MYH9 and CTNNB1 overexpression in MYH9-knockdown cells (MYH9 shRNA3 + MYH9 or MYH9 shRNA3 + CTNNB1) showed an increase in clone number and size (Figure 6D, S20A and S20B). Our anoikis analysis also revealed that MGC 80-3 cells with MYH9 knockdown (MYH9 shRNA3 + vector) had an increased rate of apoptosis when compared with that of the control group (NC + vector), whereas restoration of MYH9 or CTNNB1 expression (MYH9 shRNA3 + MYH9 or MYH9 shRNA3 + CTNNB1) blocked tumor cell apoptosis (Figure 6E, S20C). The nude mouse GC metastasis models injected with GC cells through the tail vein further showed that MYH9 knockdown (MYH9 shRNA3 + vector) significantly reduced lung and liver metastatic nodules when compared with the control group (NC + vector), whereas restoration of MYH9 or CTNNB1 expression (MYH9 shRNA3 + MYH9 or MYH9 shRNA3 + CTNNB1) increased lung and liver metastatic nodules (Figure 6F, 6G and S20D).

### Stomach-specific MYH9 overexpression enhances gastric tumorigenesis in transgenic mice

Since the role of MYH9 in human tumorigenesis is not well understood 16, 19, we generated parietal cell lineage-specific hMYH9-enhanced (LSL-hMYH9; Atp4b-cre) and Myh9-deficient (Myh9^fl/fl^; Atp4b-cre) mice by crossing mice containing a floxed STOP cassette (LSL-hMYH9) or a floxed MYH9 exon 3 with mice expressing Cre-recombinase under control of the parietal cell-specific gene (β-subunit of H^+^/K^+^ ATPase; Atp4b) promoter 30, respectively (Table S13). As expected, we observed that expression of mouse Ctnnb1 was significantly upregulated in LSL-hMYH9; Atp4b-cre mice when compared with LSL-hMYH9 littermates, while Ctnnb1 expression was significantly downregulated in Myh9^fl/fl^; Atp4b-cre mice when compared with Myh9^fl/fl^ littermates (Figure S21A). However, gastric adenomas or carcinomas did not occur in any of these mice during the two years of observation, suggesting that either hMYH9 overexpression or Myh9 knockout in the parietal cells was insufficient to transform gastric epithelial cells into tumors in mice.

**Figure 7:**
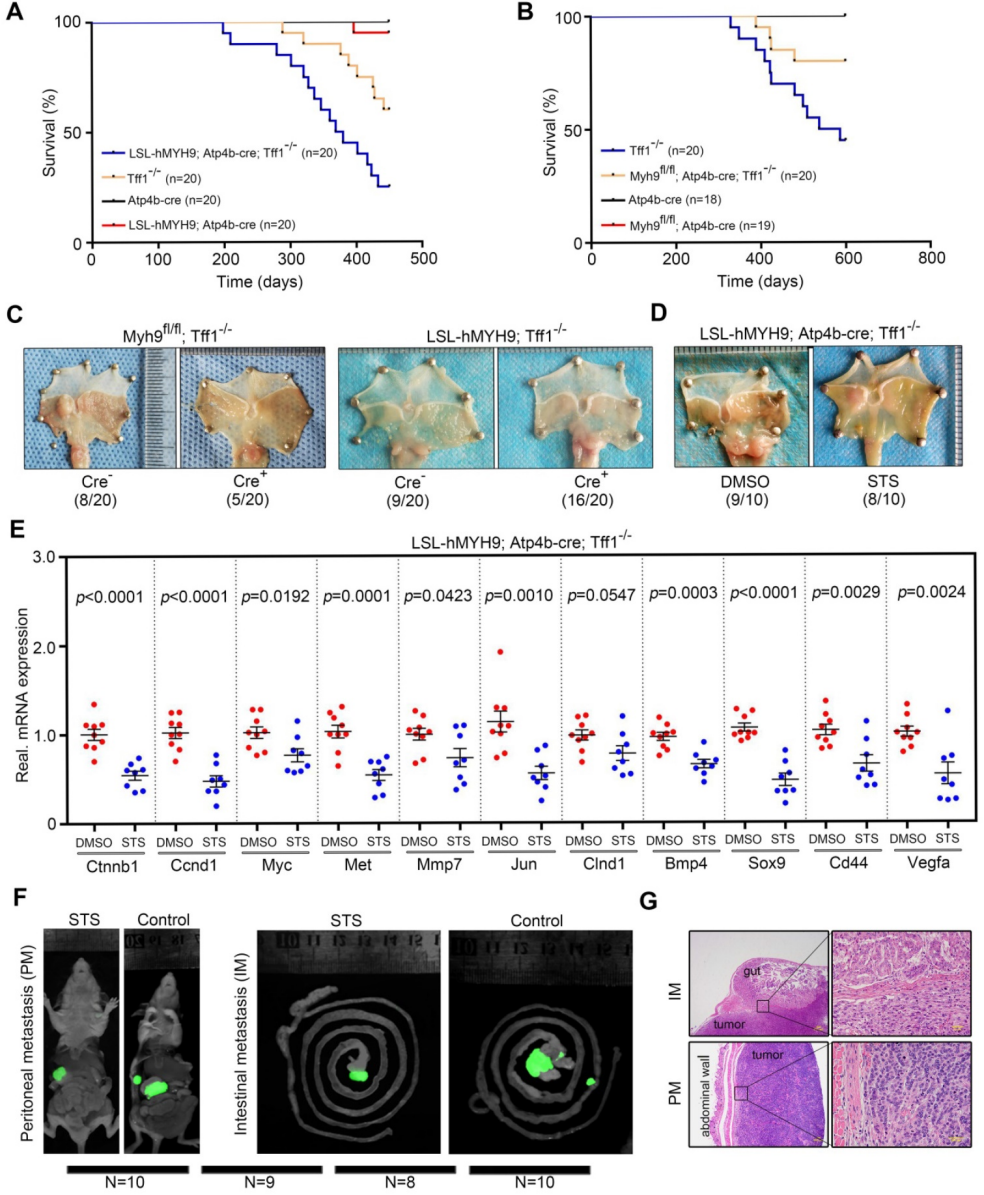
** MYH9-mediated CTNNB1 expression promotes gastric cancer progression and metastasis *in vivo*.** (A) Survival of LSL-hMYH9; Atp4b-cre; Tff1^-/-^ mice (n = 20) compared with Tff1^-/-^ mice (n = 20; *p* = 0.0099), Atp4b-cre mice (n = 20; *p* < 0.0001), or LSL-hMYH9; Atp4b-cre mice (n = 20; *p*< 0.0001) were analyzed using the log-rank test. (B) Survival of Myh9^fl/fl^; Atp4b-cre; Tff1^-/-^ mice (n = 20) compared with Tff1^-/-^ mice (n = 20; *p* = 0.027), Atp4b-cre mice (n = 18; *p* = 0.048) or Myh9^fl/fl^ mice (n = 19; *p* = 0.042) were analyzed using the log rank test. (C) Representative stomach tumors in Myh9^fl/fl^; Atp4b-cre; Tff1^-/-^ mice (5/20) at 450 days old and LSL-hMYH9; Atp4b-cre; Tff1^-/-^ mice (16/20) at 600 days old. Myh9^fl/fl^; Tff1^-/-^ mice (8/20) and LSL-hMYH9; Tff1^-/-^ mice (9/20) were shown as negative controls, respectively. (D) Representative stomach tumors in LSL-hMYH9; Atp4b-cre; Tff1^-/-^ mice treated with STS (9/10) or DMSO (8/10). (E) GC tissues from LSL-hMYH9; Atp4b-cre; Tff1^-/-^ mice treated with STS (9/10) or DMSO (8/10) were resected and subjected to qPCR analysis of β-catenin-induced mRNA. (F) Whole-body fluorescence images of metastatic tumors in an orthotropic xenograft GC nude mouse model implanted with AGS cells and treated with STS. (G) The metastatic nodules (IM, intestinal metastasis; PM, peritoneal metastasis) were detected using a hematoxylin-eosin (H&E) stain.

Trefoil factor 1 (Tff1/pS2), which could maintain the homeostasis of oxyntic epithelial progenitors, was frequently downregulated in GC 31. Tff1 KO mice consistently developed gastric adenomas with the occasional carcinoma in the pyloric antrum 32. Loss of Tff1 expression promoted β-catenin (Ctnnb1) nuclear translocation and activated β-catenin signaling and GC cell proliferation 32. Therefore, we assessed the synergistic effects of Myh9-mediated *Ctnnb1* transcription and Tff1 KO-mediated Ctnnb1 nuclear translocation on GC progression by crossing LSL-hMYH9; Atp4b-cre mice or Myh9^fl/fl^; Atp4b-cre mice with Tff1^-/-^ mice. We randomly selected 20 LSL-hMYH9; Atp4b-cre; Tff1^-/-^ mice, 20 Tff1^-/-^ mice, 20 Atp4b-cre mice, and 20 LSL-hMYH9; Atp4b-cre mice to observe for up to 450 days. The results showed that conditional hMYH9 overexpression significantly increased tumor incidence but reduced mouse survival of Tff1^-/-^ mice, when compared with the control littermates (Figure 7A). We also randomly selected 20 Tff1^-/-^ mice, 20 Myh9^fl/fl^; Atp4b-cre; Tff1^-/-^ mice, 18 Atp4b-cre mice, and 19 Myh9^fl/fl^; Atp4b-cre mice to observe for up to 600 days. The results revealed that conditional Myh9 knockout significantly decreased tumor incidence and prolonged survival of Tff1^-/-^ mice, when compared with the control littermates (Figure 7B).

Furthermore, we randomly selected an additional 20 Myh9^fl/fl^; Atp4b-cre; Tff1^-/-^ mice, 20 Myh9^fl/fl^; Tff1^-/-^ mice, 20 LSL-hMYH9; Atp4b-cre; Tff1^-/-^ mice and 20 LSL-hMYH9; Tff1^-/-^ mice to observe for 400 days. We found that the tumor incidence in Myh9^fl/fl^; Atp4b-cre; Tff1^-/-^ mice was significantly reduced (5/20, 25%) and the tumor size was smaller, when compared with Myh9^fl/fl^; Tff1^-/-^ mice (8/20, 40%; Figure 7C, S21B). Conversely, the tumor incidence in LSL-hMYH9; Atp4b-cre; Tff1^-/-^ mice was significantly enhanced (16/20, 80%) and the tumor size was larger, when compared with LSL-hMYH9; Tff1^-/-^ mice (9/20, 45%; Figure 7C, S21B). We also identified three LSL-hMYH9; Atp4b-cre; Tff1^-/-^ mice with advanced gastric cancer (Figure S21C, S21D), suggesting that hMYH9 promoted GC progression. To explore the association of Myh9 with expression of Ctnnb1 and Wnt/β-catenin-targeted genes, we performed qPCR on the tumor tissues listed above and found that conditional hMYH9 expression in Tff1^-/-^ mice upregulated the expression of Wnt/β-catenin targeted genes, while conditional Myh9 knockout in Tff1^-/-^ mice significantly downregulated the expression of Wnt/β-catenin targeted genes (Figure S21E, S21F). This suggests that hMYH9- or Myh9-mediated *Ctnnb1* transcription and Tff1 KO-mediated β-catenin nuclear translocation synergistically promoted GC progression by increasing Wnt/β-catenin signaling activation in these transgenic mice.

To explore the treatment effect of STS* in vivo*, we selected an additional 20 four-week-old LSL-hMYH9; Atp4b-cre^+^; Tff1^-/-^ mice to randomly divide into STS treatment and control (DMSO) groups. We assessed the maximum tolerated dose (MTD) of STS and determined the dosage regimen (i.p. injection of 0.6 mg/kg/daily for five consecutive days, every two months for 420 days; details in Supplementary Methods; Table S14) in four-week-old male C57BL/6 mice using dose-escalating experiments 33. Our data showed that STS dramatically reduced the tumor size (Figs. 7D, S22A) and downregulated levels of the Wnt/β-catenin-targeted genes (Figure 7E) but failed to reduce tumor incidence (Fig 7F). With the absence of peritoneal metastasis in our transgenic mouse models of GC during our observation period, we also assessed the treatment effect of STS on GC metastasis using the orthotropic xenograft GC mouse model as described in our previous study 16. We assessed that the MTD of STS was 0.6 mg/kg (five consecutive i.p. injections; details in Supplementary Methods; Table S14) in five-week-old female BALB/c nu/nu mice using dose-escalating experiments 33. After STS treatment, we found that the tumor volume in the STS-treated mice was smaller compared to the control group (Figure 7F, S22B), and the number of metastatic nodules was also significantly reduced in the STS-treated group (Figure 7F, 7G and S22C). Furthermore, we detected *p*-MYH9 1943 and β-catenin expression in primary tumors of the STS-treated group by western blot, which was significantly downregulated compared with the primary tumors of the control group (Figure S22D).

## Discussion

In this study, we found that nuclear MYH9 conferred anoikis resistance to GC cells and promoted GC cell metastasis by identifying the *CTNNB1* promoter to induce β-catenin transcription and increasing activation of canonical Wnt/β-catenin signaling* in vitro* and *in vivo*. We further confirmed that staurosporine decreased nuclear MYH9 phosphorylation at S1943 to inhibit the MYH9-CTNNB1 axis-mediated canonical Wnt/β-catenin signaling activation, which therefore might serve as a potential inhibitor for GC peritoneal metastasis.

MYH9 is a well-known cytoplasmic effector molecule in Rho-ROCK signaling pathways involved in cancer cell invasion and metastasis 13. MYH9 was recently found to be localized on the cell surface, and acts as a functional entry receptor for herpes simplex virus-1 34, but it is also localized in the nucleus where it can regulate post-transcriptional p53 stabilization in squamous cell carcinomas 19 or bind to long non-coding RNA to regulate activity of a bidirectional promoter in thyroid cancer cells 35. Our current study first confirmed that MYH9 was mainly localized to the cell nucleus in GC cells and was bound directly to the *CTNNB1* promoter. This binding activated *CTNNB1* transcription and lead to GC cell anoikis resistance. Anoikis resistance has been reported to be caused by abnormal expression of certain genes (such as *TrkB*, *CTNNB1* and *HSP70A1A*) or pathways (such as the Wnt/β-catenin pathway), and enhance the metastatic ability of cancer cells 29, 36-38. In this study, we uncovered that MYH9-induced *CTNNB1* transcription promoted the peritoneal metastasis of gastric cancer cells. We also unexpectedly discovered the nuclear localization signals and DNA-binding domain of MYH9 in GC cells for the first time. Since β-catenin (CTNNB1) is an important regulator of the Wnt/β-catenin signaling pathway, and many transcriptional regulators have been shown to regulate the transcription of *CTNNB1* (such as MALAT1, CDYL, Hsp70 and p14^ARF^ 39-41), nuclear MYH9-induced *CTNNB1* transcription in GC cells found in this study further contributed to our understanding of the mechanisms regulating the transcription of *CTNNB1*.

MYH9 has also been shown to contribute to a secretory phenotype that reshaped the local environment and vasculature to support growth of melanoma cells 42, and is required for T lymphocyte migration 43, maturation of the immunological synapse 43 and the cytotoxicity of natural killer cells 44. Therefore, MYH9 knockdown in immune cells might decrease tumor immunosurveillance and serve as a tumor suppressor. Previous studies of MYH9 in cancers have been controversial 16-19. To avoid the interference of stromal cells, especially immune cells, we introduced scRNA-seq technology and confirmed that *MYH9* mRNA is upregulated in metastatic GC cells when compared to normal gastric epithelial cells or primary GC cells. Moreover, we used conditional transgenic mice to specifically knockout or overexpress the *MYH9* gene in mouse gastric epithelial cells and further confirmed that MYH9 promotes GC progression in the Tff1^-/-^ GC mouse model.

To fully describe the GC metastasis microenvironment, it was necessary to analyze more advanced GC cases using single-cell sequencing. Although our transgenic GC mouse models (LSL-hMYH9; Atp4b-cre; Tff1^-/-^ mice and Myh9^fl/fl^; Atp4b-cre; Tff1^-/-^ mice) supported the finding that MYH9-mediated *CTNNB1* transcription promotes tumor progression (proliferation and invasion) and shortens the survival of Tff1^-/-^ mice, we failed to find peritoneal metastasis in our models. Therefore, other GC mouse models prone to peritoneal metastasis should to be introduced for further study 45. Although STS was confirmed to significantly inhibit nuclear MYH9 phosphorylation at S1943 and GC metastasis, it is a pan-kinase inhibitor, and how to improve the intracellular nuclear targeting of STS requires further research. The nuclear MYH9-mediated gene expression profile and signal pathways in GC cells are still poorly understood, and other MYH9-associated nuclear biological processes, such as DNA replication, heterochromatic breaks or DNA repair 46 also require further exploration. In addition, since β-catenin was shown to be the important regulator of cell death and peritoneal metastasis of GC cells and its transcription was regulated by many factors (such as MALAT1, CDYL, Hsp70 and p14^ARF^) 39-41 , it should be confirmed in future work whether GC cells have a molecular interaction network that influences the actions of β-catenin 41.

In summary, our study illustrates that nuclear MYH9 S1943 phosphorylation, inhibited by staurosporine, promotes GC cell anoikis resistance and metastasis by increasing *CTNNB1* transcription and activating Wnt/β-catenin signaling (Figure S23, S24). These findings identify staurosporine as a novel inhibitor for the treatment of advanced GC.

## Methods

### Human samples and patient information

This study was approved by the Ethics Committee of Nanfang hospital (Guangzhou, China) and written informed consent was obtained from individual patients before enrollment into this study. GC and normal tissues without any pre-surgical chemoradiation therapy were collected from patients, snap-frozen, and maintained at -80 °C at the General Surgery Department, Nanfang Hospital, Southern Medical University (Guangzhou, China) between January 2012 and October 2018. GC was histologically diagnosed using endoscopic biopsy combined with surgical specimens by two pathologists of Nanfang Hospital according to American Joint Committee on Cancer TNM Staging Classification for Carcinoma of the Stomach (7th ed., 2010). The clinicopathological data for each patient were retrieved from their medical records. Moreover, it is worth mentioning that patients with advanced GC and peritoneal metastasis only underwent palliative resection to stop tumor-induced bleeding and/or remove obstruction. Samples of these procedures are rare and are mainly used for proteomics analysis, qPCR or single-cell transcriptome analysis.

### Cell lines and culture

Human GC cell lines (MGC 80-3, MKN-45, HGC-27, AGS, NCI-N87, BGC-823, AGS, SNU-5, KATO III and SGC-7901), human embryonic kidney 293 cell line (HEK-293T), a human gastric epithelial cell line (GES-1), human colorectal cancer cell lines (SW620, HT-29, SW480 and Caco-2), a human colon cell line (FHC), a rat intestinal crypt cell line (IEC-6) and a murine colon cancer cell line (CT-26) were obtained from the Type Culture Collection of the Chinese Academy of Sciences (Shanghai, China). These cell lines were cultured in Roswell Park Memorial Institute medium-1640 (RPMI-1640) for MGC 80-3, MKN45, HGC-27, BGC-823, SGC-7901, HEK-293T, GES-1, FHC, SW620, HT-29, SW480, IEC-6 and CT-26 cells, F12 medium for AGS cell, or Dulbecco's Modified Eagle's Medium (DMEM) for NCI-N87, KATOIII, SNU-5 and Caco-2 cells. All media was supplemented with 10% fetal bovine serum (FBS) and cells were cultured in a humidified incubator with 5% CO_2_ at 37 °C.

### Animal models

The orthotropic GC model and GC lung metastasis model were established in athymic BALB/c nu/nu mice (China, Guangzhou, China). A transgenic GC mouse model with parietal cell-specific MYH9 overexpression was established by crossing LoxP-Stop-LoxP human MYH9-transgenic mice (LSL-hMYH9) with ATP4b-cre mice and Tff1 KO mice. The transgenic GC mouse model with parietal cell-specific Myh9 KO was established by crossing Myh9 conditional KO mice (Myh9^fl/fl^) with ATP4b-cre mice and Tff1-KO mice. More detailed information about these models is provided in the Supplementary Methods.

### Statistical analysis

All experiments were performed at least in triplicate and repeated three times, while all representative images were obtained from at least three independent experiments. All data were presented as mean ± standard error of the mean (SEM), and all statistical analyses were performed using GraphPad Prism (version 6; San Diego, CA, USA) or SPSS statistical software package (SPSS^®^ version 16.0, SPSS Inc., Chicago, IL, USA). The differences between two groups were assessed using Student's *t*-test. The association of MYH9 mRNA with overall survival (OS) or disease-free survival (DFS) of GC patients was shown using the Kaplan-Meier survival analysis of the GC dataset from KMplot (http://kmplot.com). The difference of survival rate between two groups was analyzed using the log-rank test. Association of MYH9 expression with clinicopathological data from patients was assessed using a non-parametric χ^2^ test. The Spearman's rank correlation coefficient was used to assess the association of *MYH9* mRNA with *CTNNB1* mRNA level. *p < 0.05, **p < 0.01 and ***p < 0.001 were all considered statistically significant.

All other details related to the Materials and Methods are provided in the Supplementary Information.

## Supplementary Material

Supplementary figures and tables.

## Abbreviations

2D-DIGE: Two-dimensional fluorescence differential gel electrophoresis
CTNNB1: Catenin beta-1
DBD: DNA-binding domain
DEP: Differentially expressed protein
GC: Gastric cancer
MALDI-TOF/TOF MS: Matrix-assisted laser desorption time-of-flight mass spectrometry
MTD: Maximum tolerated dose
MYH9: myosin IIa
NLS: Nuclear localization signal
STS: Staurosporine
scRNA-seq: single-cell RNA sequencing
TALEN: Transcription activator-like effector nucleases technology

## References

[1] Yu J, Huang CM, Sun YH, Su XQ, Cao H, Hu JK (2019). Effect of Laparoscopic vs Open Distal Gastrectomy on 3-Year Disease-Free Survival in Patients With Locally Advanced Gastric Cancer The CLASS-01 Randomized Clinical Trial. JAMA.

[2] Hu Y, Huang C, Sun Y, Su X, Cao H, Hu J (2016). Morbidity and Mortality of Laparoscopic Versus Open D2 Distal Gastrectomy for Advanced Gastric Cancer: A Randomized Controlled Trial. J Clin Oncol.

[3] Alyami M, Hubner M, Grass F, Bakrin N, Villeneuve L, Laplace N (2019). Pressurised intraperitoneal aerosol chemotherapy: rationale, evidence, and potential indications. Lancet Oncol.

[4] Bray F, Ferlay J, Soerjomataram I, Siegel RL, Torre LA, Jemal A (2018). Global cancer statistics 2018: GLOBOCAN estimates of incidence and mortality worldwide for 36 cancers in 185 countries. CA Cancer J Clin.

[5] Shah MA (2015). Update on metastatic gastric and esophageal cancers. J Clin Oncol.

[6] Wong SS, Kim KM, Ting JC, Yu K, Fu J, Liu S (2014). Genomic landscape and genetic heterogeneity in gastric adenocarcinoma revealed by whole-genome sequencing. Nat Commun.

[7] Ooi CH, Ivanova T, Wu J, Lee M, Tan IB, Tao J (2009). Oncogenic pathway combinations predict clinical prognosis in gastric cancer. PLoS Genet.

[8] Bass AJ, Thorsson V, Shmulevich I, Reynolds SM, Miller M, Bernard B (2014). Comprehensive molecular characterization of gastric adenocarcinoma. Nature.

[9] Wang K, Yuen ST, Xu J, Lee SP, Yan HH, Shi ST (2014). Whole-genome sequencing and comprehensive molecular profiling identify new driver mutations in gastric cancer. Nat Genet.

[10] Fidler IJ (2003). The pathogenesis of cancer metastasis: the 'seed and soil' hypothesis revisited. Nat Rev Cancer.

[11] Zhuo W, Liu Y, Li S, Guo D, Sun Q, Jin J (2019). Long Noncoding RNA GMAN, Up-regulated in Gastric Cancer Tissues, Is Associated With Metastasis in Patients and Promotes Translation of Ephrin A1 by Competitively Binding GMAN-AS. Gastroenterology.

[12] Lin C, He H, Liu H, Li R, Chen Y, Qi Y (2019). Tumour-associated macrophages-derived CXCL8 determines immune evasion through autonomous PD-L1 expression in gastric cancer. Gut.

[13] Vicente-Manzanares M, Ma X, Adelstein RS, Horwitz AR (2009). Non-muscle myosin II takes centre stage in cell adhesion and migration. Nat Rev Mol Cell Biol.

[14] Balduini CL, Pecci A, Savoia A (2011). Recent advances in the understanding and management of MYH9-related inherited thrombocytopenias. Br J Haematol.

[15] Conti MA, Even-Ram S, Liu C, Yamada KM, Adelstein RS (2004). Defects in cell adhesion and the visceral endoderm following ablation of nonmuscle myosin heavy chain II-A in mice. J Biol Chem.

[16] Ye G, Huang K, Yu J, Zhao L, Zhu X, Yang Q (2017). MicroRNA-647 Targets SRF-MYH9 Axis to Suppress Invasion and Metastasis of Gastric Cancer. Theranostics.

[17] Rokutan H, Hosoda F, Hama N, Nakamura H, Totoki Y, Furukawa E (2016). Comprehensive mutation profiling of mucinous gastric carcinoma. J Pathol.

[18] Park SY, Kim H, Yoon S, Bae JA, Choi SY, Jung YD (2014). KITENIN-targeting microRNA-124 suppresses colorectal cancer cell motility and tumorigenesis. Mol Ther.

[19] Schramek D, Sendoel A, Segal JP, Beronja S, Heller E, Oristian D (2014). Direct in vivo RNAi screen unveils myosin IIa as a tumor suppressor of squamous cell carcinomas. Science.

[20] Liang S, He L, Zhao X, Miao Y, Gu Y, Guo C (2011). MicroRNA let-7f inhibits tumor invasion and metastasis by targeting MYH9 in human gastric cancer. PloS one.

[21] Williams ED, Gao DC, Redfern N, Thompson EW (2019). Controversies around epithelial-mesenchymal plasticity in cancer metastasis. Nat Rev Cancer.

[22] Dongre A, Weinberg RA (2019). New insights into the mechanisms of epithelial-mesenchymal transition and implications for cancer. Nat Rev Mol Cell Bio.

[23] Hofmann WA, Stojiljkovic L, Fuchsova B, Vargas GM, Mavrommatis E, Philimonenko V (2004). Actin is part of pre-initiation complexes and is necessary for transcription by RNA polymerase II. Nat Cell Biol.

[24] Li Q, Sarna SK (2009). Nuclear myosin II regulates the assembly of preinitiation complex for ICAM-1 gene transcription. Gastroenterology.

[25] Zhang N, Wei P, Gong A, Chiu WT, Lee HT, Colman H (2011). FoxM1 promotes beta-catenin nuclear localization and controls Wnt target-gene expression and glioma tumorigenesis. Cancer cell.

[26] Yanagihara N, Tachikawa E, Izumi F, Yasugawa S, Yamamoto H, Miyamoto E (1991). Staurosporine: an effective inhibitor for Ca2+/calmodulin-dependent protein kinase II. J Neurochem.

[27] Chang HR, Nam S, Kook MC, Kim KT, Liu X, Yao H (2016). HNF4alpha is a therapeutic target that links AMPK to WNT signalling in early-stage gastric cancer. Gut.

[28] Orford K, Orford CC, Byers SW (1999). Exogenous expression of beta-catenin regulates contact inhibition, anchorage-independent growth, anoikis, and radiation-induced cell cycle arrest. J Cell Biol.

[29] Douma S, Van Laar T, Zevenhoven J, Meuwissen R, Van Garderen E, Peeper DS (2004). Suppression of anoikis and induction of metastasis by the neurotrophic receptor TrkB. Nature.

[30] Zhao Z, Hou N, Sun Y, Teng Y, Yang X (2010). Atp4b promoter directs the expression of Cre recombinase in gastric parietal cells of transgenic mice. J Genet Genomics.

[31] Feng G, Zhang Y, Yuan H, Bai R, Zheng J, Zhang J (2014). DNA methylation of trefoil factor 1 (TFF1) is associated with the tumorigenesis of gastric carcinoma. Mol Med Rep.

[32] Karam SM, Tomasetto C, Rio MC (2004). Trefoil factor 1 is required for the commitment programme of mouse oxyntic epithelial progenitors. Gut.

[33] Tang WL, Chen WC, Roy A, Undzys E, Li SD (2016). A Simple and Improved Active Loading Method to Efficiently Encapsulate Staurosporine into Lipid-Based Nanoparticles for Enhanced Therapy of Multidrug Resistant Cancer. Pharm Res.

[34] Arii J, Goto H, Suenaga T, Oyama M, Kozuka-Hata H, Imai T (2010). Non-muscle myosin IIA is a functional entry receptor for herpes simplex virus-1. Nature.

[35] Wang Y, He H, Li W, Phay J, Shen R, Yu L (2017). MYH9 binds to lncRNA gene PTCSC2 and regulates FOXE1 in the 9q22 thyroid cancer risk locus. Proc Natl Acad Sci U S A.

[36] Kasioumi P, Vrazeli P, Vezyraki P, Zerikiotis S, Katsouras C, Damalas A (2019). Hsp70 (HSP70A1A) downregulation enhances the metastatic ability of cancer cells. Int J Oncol.

[37] Liotta LA, Kohn E (2004). Anoikis: cancer and the homeless cell. Nature.

[38] Tan YJ, Lin KL, Zhao Y, Wu QJ, Chen DP, Wang J (2018). Adipocytes fuel gastric cancer omental metastasis via PITPNC1-mediated fatty acid metabolic reprogramming. Theranostics.

[39] Li GQ, Fang YX, Liu Y, Meng FR, Wu X, Zhang CW (2019). MALAT1-Driven Inhibition of Wnt Signal Impedes Proliferation and Inflammation in Fibroblast-Like Synoviocytes Through CTNNB1 Promoter Methylation in Rheumatoid Arthritis. Hum Gene Ther.

[40] Zhou X, Xu B, Zhang D, Jiang X, Chang HM, Leung PCK (2020). Loss of CDYL Results in Suppression of CTNNB1 and Decreased Endometrial Receptivity. Front Cell Dev Biol.

[41] Damalas A, Velimezi G, Kalaitzakis A, Liontos M, Papavassiliou AG, Gorgoulis V (2011). Loss of p14(ARF) confers resistance to heat shock- and oxidative stress-mediated cell death by upregulating beta-catenin. Int J Cancer.

[42] Georgouli M, Herraiz C, Crosas-Molist E, Fanshawe B, Maiques O, Perdrix A (2019). Regional Activation of Myosin II in Cancer Cells Drives Tumor Progression via a Secretory Cross-Talk with the Immune Microenvironment. Cell.

[43] Kumari S, Vardhana S, Cammer M, Curado S, Santos L, Sheetz MP (2012). T Lymphocyte Myosin IIA is Required for Maturation of the Immunological Synapse. Front Immunol.

[44] Sanborn KB, Mace EM, Rak GD, Difeo A, Martignetti JA, Pecci A (2011). Phosphorylation of the myosin IIA tailpiece regulates single myosin IIA molecule association with lytic granules to promote NK-cell cytotoxicity. Blood.

[45] Seidlitz T, Chen YT, Uhlemann H, Scholch S, Kochall S, Merker SR (2019). Mouse Models of Human Gastric Cancer Subtypes With Stomach-Specific CreERT2-Mediated Pathway Alterations. Gastroenterology.

[46] Caridi CP, D'Agostino C, Ryu T, Zapotoczny G, Delabaere L, Li X (2018). Nuclear F-actin and myosins drive relocalization of heterochromatic breaks. Nature.

