# Supplementary material for: Nuclear MYH9-induced CTNNB1 transcription, targeted by staurosporin, promotes gastric cancer cell anoikis resistance and metastasis
Source: Theranostics. 2020 Jun 12;10(17):7545–60. doi: 10.7150/thno.46001 (PMC7359096; doi:10.7150/thno.46001)
Supplement: Supplementary file 1 — Supplementary figures and tables. [file thnov10p7545s1.zip › supplementary files/Supplementary files.docx]

**Supplementary information**

**Key resources table**

| Reagent or Resource | Source | Identifier |
| --- | --- | --- |
| Antibodies |  |  |
| Anti-MYH9 (H-40) (1:1000) | Santa Cruz Biotechnology | Cat. #sc-98978 |
| Anti-Myosin II (1:1000) | Cell Signaling Technology | Cat. #3403 |
| Anti-β-catenin (1:2000) | BD | Cat. #[610154](http://www.bdbiosciences.com/ptProduct.jsp?prodId=26399&key=610154&param=search&mterms=true&from=dTable) |
| Anti-β-catenin (B-9) (1:500) | Santa Cruz Biotechnology | Cat. #sc-376841 |
| Anti-GAPDH (1:500) | Santa Cruz Biotechnology | Cat. #sc-32233 |
| Anti-Vimentin (1:500) | Santa Cruz Biotechnology | Cat. #sc-66002 |
| Anti-E-cadherin (1:500) | Santa Cruz Biotechnology | Cat. #sc-71007 |
| Anti-SNAIL (1:500) | Proteintech | Cat. #13099-1-AP |
| Anti-phospho-Myosin IIa (Ser1943) (1:1000) | Cell Signaling Technology | Cat. #5026 |
| Anti-phospho-Myosin IIa (Ser1916) (1:1000) | BBI Life Sciences | Cat. #J508021-0001 |
| Anti-β-Actin (C4) (1:500) | Santa Cruz Biotechnology | Cat. #sc-47778 |
| Anti-MYL9 (A-10) (1:500) | Santa Cruz Biotechnology | Cat. #sc-376606 |
| Anti-RNA Polymerase II Antibody (ChIP) | Thermo Scientific | Cat. #26156 |
| Normal Rabbit IgG (ChIP) | Thermo Scientific | Cat. #26156 |
| Anti-TFIIB (D-3) (1:500) | Santa Cruz Biotechnology | Cat. #sc-271736 |
| Anti-Lamin B1 (1:3000) | Proteintech | Cat. #12987-1-AP |
| Anti-SRF, clone 1E1 (1:500) | Millipore | Cat. #MAB4369 |
| Anti-GFP (1:200) | Santa Cruz Biotechnology | Cat. #sc-101525 |
| Anti-Mouse IgG H&L (HRP) (1:2000) | Abcam | Cat. #Ab136815 |
| Anti-Rabbit IgG H&L (HRP) (1:2000) | Abcam | Cat. #Ab136817 |
| Anti-Rabbit IgG (H+L), F(ab')2 Fragment (Alexa Fluor® 555 Conjugate) (red) (1:1000) | Cell Signaling Technology | Cat. #4413 |
| Anti-Mouse IgG (H+L), F(ab')2 Fragment (Alexa Fluor® 488 Conjugate) (green) (1:1000) | Cell Signaling Technology | Cat. #4408 |
| Anti-Mouse IgG (H+L), F(ab')2 Fragment (Alexa Fluor® 555 Conjugate) (red) (1:1000) | Cell Signaling Technology | Cat. #4409 |
| Anti-Rabbit IgG (H+L), F(ab')2 Fragment (Alexa Fluor® 488 Conjugate) (green) (1:1000) | Cell Signaling Technology | Cat. #4412 |
| Anti-Rabbit IgG (whole molecule)-Gold (10nm colloidal gold) (1:50) | Sigma-Aldrich | Cat. #G7402 |
| Chemicals, Peptides and Recombinant Proteins | | |
| Invitrogen™ Lipofectamine® 3000  Transfection Reagent | Invitrogen | Cat. #L3000015 |
| Puromycin | Sigma-Aldrich | Cat. #P7255 |
| FITC phalloidin | YEASEN | Cat. #40735ES75 |
| Low Melting Point Agarose | YEASEN | Cat. #10214ES08 |
| TRIzol™ Reagent (Invitrogen) | Invitrogen | Cat. #15596026 |
| PrismScript RT reagent Kit | TaKaRa | Cat. #RR036A |
| SYBR Premix Ex Taq | TaKaRa | Cat. #RR820A |
| Recombinant Human Wnt-3a Protein | R&D Systems | Cat. #5036-WN-500 |
| Blebbistatin | Selleck | Cat. #S7099 |
| ML-7 hydrochloride | MedChemExpress (MCE) | Cat. #HY-15417 |
| STS | MCE | Cat. #HY-15141 |
| Y-27632 dihydrochloride | MCE | Cat. #HY-10583 |
| CX-4945 | MCE | Cat. #HY-50855 |
| MCLR | Enzo Life Sciences | Cat. #ALX-350-012-C050 |
| 4',6-Diamidino-2-Phenylindole (DAPI) | Thermo Scientific | Cat. #D1306 |
| Recombinant DNA |  |  |
| CTNNB1-EGFP | Genechem | Cat. #GOSE51722 |
| Vector-EGFP (GV230) | Genechem | Cat. #CON083 |
| MYH9 lentivirus particle (LV-MYH9) | GeneCopoeia | Cat. #LPP-T1335-Lv105-A00 |
| NC-EGFP (control lentivirus particle LV-NC) | GeneCopoeia | Cat. #LPP-eGFP-Lv105-100 |
| MYH9-EGFP | GeneCopoeia | Cat. #EX-T1335-M98-5 |
| ACTB-EGFP | GeneCopoeia | Cat. #EX-T1780-M98 |
| MYL9-EGFP | GeneCopoeia | Cat. #EX-A4086-M98 |
| Vector-EGFP (M98) | GeneCopoeia | Cat. #EX-NEG-M98 |
| TOPflash (TCF Reporter Plasmid) | Merck Millipore | Cat. #21-170 |
| FOPflash (mutant TCF binding sites) | Merck Millipore | Cat. #21-169 |
| Renilla luciferase reporter plasmid (pR-TK) | Promega | Cat. #E2241 |
| Critical Commercial Assays |  |  |
| 2D Clean-up Kit | GE Healthcare | Cat. #80-6484-51 |
| 2D Quant Kit | GE Healthcare | Cat. #80-6483-56 |
| CyDye™ DIGE Fluor Minimal Labeling Kit | GE Healthcare | Cat. ##25-8010-65 |
| FastTALETM TALEN Assembly Kit | SIDANSAI Biotechnology | Cat. #1802-030 |
| PrismScript RT reagent Kit | TaKaRa | Cat. #RR036A |
| SYBR Premix Ex Taq | TaKaRa | Cat. #RR820A |
| Mammalian Protein Extraction Kit | CWBIO | Cat. #CW0889 |
| Tissue Protein Extraction Kit | CWBIO | Cat. #CW0891 |
| BCA Protein Assay Kit | CWBIO | Cat. #CW0014S |
| NE-PER™ Nuclear and Cytoplasmic Extraction Reagents | Thermo Scientific | Cat. #78835 |
| Immobilon Western HRP | Millipore | Cat. #WBKLS0500 |
| Dual-Glo® Luciferase Assay System | Promega | Cat. #E2940 |
| GeneTailor™ Site-Directed Mutagenesis System | Invitrogen | Cat. #12397-022 |
| Pierce™ Agarose ChIP Kit | Thermo Scientific | Cat. #26156 |
| Pierce™ Biotin 3' End DNA Labeling Kit | Thermo Scientific | Cat. #89818 |
| CytoSelect™ 24-Well Anoikis Assay | Cell BioLabs | Cat. #CBA-080 |
| Cell Counting Kit-8 (CCK-8) | BestBio | Cat. #BB-4202-2 |
| Cell Cycle Detection Kit | KEYGEN | Cat. #KGA512 |
| Annexin V-APC/7-AAD Apoptosis Kit | MultiSciences Biotech | Cat. #AP105-30 |
| Mouse Direct PCR Kit | Biotool | Cat. #B40015a |
| Software and Algorithms |  |  |
| Progenesis Same Spot Software | Non-Linear Dynamics, USA | N/A |
| Flex Analysis 2.4 software | Bruker Daltonics, Germany | N/A |
| ImageJ | NIH | N/A |
| GraphPad Prism | GraphPad Software | Version 5 and 6 |
| SPSS statistical software package | SPSS | Version 16.0 |
| Pymol molecular graphics software | Schrödinger | Version 1.8 |
| Websites |  |  |
| c-Bioportal (TCGA) | https://www.cbioportal.org | |
| GENT | http://medical-genome.kribb.re.kr/GENT | |
| KMplot | http://kmplot.com | |
| GEPIA | http://gepia.cancer-pku.cn/index.html | |
| NCBI | https://www.ncbi.nlm.nih.gov/ | |
| Swiss-Model online server | http://swissmodel.expasy.org/ | |
| Modeller | http://salilab.org/modeller/ | |
| PDB | http://www.rcsb.org/ | |
| PSORT II | http://www.genscript.com/cgi-bin/tools/psort2.pl | |
| PROCHECK | http://servicesn.mbi.ucla.edu/PROCHECK/ | |
| ZDOCK server | http://zdock.umassmed.edu/ | |
| R software | https://www.R-project.org/ | |

**Supplementary Methods**

**Proteomic analysis**

Tissue specimens from six GC patients with peritoneal metastases who underwent palliative gastrectomy without any chemo-radiation treatments were collected for our proteomic analysis. The specimens included primary GC tissues, peritoneal metastatic tissues and their corresponding normal gastric mucosae. These tissue samples (100 mg each) were grounded into tissue powder in liquid nitrogen and lysed at 4 °C for 1 h in lysis buffer (7 M urea, 2 M thiourea, 2% NP-40, 1% Triton X-100, 100 mM dithiothreitol, 5 mM phenylmethane sulfonyl fluoride (PMSF), 4% 3-[(3-cholamidopropyl)dimethylammonio]-1-propanesulfonate (CHAPS), 0.5 mM ethylene diaminetetra acetic acid (EDTA), 40 mM Tris-HCl and 2% pharmalyte at pH 8.5). Lysed tissue samples were then centrifuged at 12,000 rpm for 30 min at 4 °C. The supernatants were then collected and precipitated with the 2D Clean-up Kit (Cat. #80-6484-51; GE Healthcare, Fairfield, CT, USA) according to the manufacturer’s protocol, and re-suspended in the lysis buffer. Next, the concentration of these protein samples was measured using the 2D Quant Kit (Cat. #80-6483-56; GE Healthcare) according to the manufacturer’s instructions.

For DIGE, 50 μg protein samples were labeled with the CyDye™ DIGE Fluor Minimal Labeling Kit (Cat. #25-8010-65; GE Healthcare) according to the minimal labeling protocol from the manufacturer. The CyDye labeling scheme is shown in Table S1. Briefly, the protein samples were paired to generate six sample pairs and then labeled with Cy3 or Cy5. Next, an equal amount of each protein sample was mixed to generate an internal standard, and 50 μg of this internal standard was labeled with Cy2. Therefore, each gel consisted of one Cy3-labeled sample, one Cy5-labeled sample and one Cy2-labeled internal standard sample. Next, the 2D-DIGE separation was conducted as previously described [[1](#_ENREF_1)]. In summary, all labeled samples were pooled together, loaded onto IPG strips (13 cm, pH 3–10) and rehydrated for 15 h to yield rehydrated strips. These strips were subjected to iso-electric focusing and separated on 13% poly-acrylamide gels. This procedure was repeated three times with alternative dye swapping to minimize experimental error.

To identify the DEP spots in the gels, the labeled gels were scanned using a Typhoon TRIO Variable Mode Imager (Amersham Biosciences, Piscataway, NJ, USA) and analyzed using the Progenesis Same Spot Software (Non-Linear Dynamics, Durham, NC, USA). Next, the labeled gels were stained with silver stain, and the protein spots of interest were manually excised from the gel and digested with trypsin for MALDI-TOF/TOF MS analysis. The data were processed and analyzed using Flex Analysis 2.4 software (Bruker Daltonics, Germany).

**Single-cell transcriptome analysis**

*(i) Samples and processing*

Human GC tissues were ground and enzymatically digested for 30 min using the cocktail dissociation solution (Sigma-Aldrich) according to the manufacturer’s instructions. Next, the tissues were incubated with DMEM containing 10% FBS and then filtered through a 70 mm nylon filter (BD Falcon, Bedford, MA, USA). The filtered cells were incubated in 1x Red Blood Cell Removal Solution (Cat. #64010-00-100; BioGems International, Westlake Village, CA, USA) for 5 min to remove the red blood cells. Next, the dead cells were removed using a Dead Cell Removal Kit (Cat. #130-090-101; Miltenyi Biotec, Auburn, CA, USA) according to the manufacturer’s protocol.

*(ii) Library preparation and DNA sequencing*

These samples were further treated with the 10x Genomics Single Cell 3' Reagent Kit v2 with the help of Guangzhou Saliai Stem Cell Science and Technology Co. Ltd (Guangzhou, China). Briefly, the samples were washed twice in PBS (Invitrogen) containing 0.04% BSA (Sigma-Aldrich), centrifuged and re-suspended in PBS containing 0.04% BSA, then filtered through a 40 mm nylon filter (BD Falcon). Next, the cell viability was assessed using trypan blue (Thermo Fisher Scientific) and the appropriate volume of each sample was determined and loaded into the 10x Genomics single-cell-A chip (120236; 10x Genomics Inc., Pleasanton, CA, USA). Next, the chips were transferred into a pre-chilled 8-well tube (Eppendorf, Hamburg, Germany) and heat-sealed for the reverse transcription in a Veriti 96-well thermal cycler (Thermo Fisher Scientific). The cDNA was recovered using the Recovery Agent obtained by 10x Silane DynaBead clean-up (Thermo Fisher Scientific), performed according to the user guide. The purified cDNA samples were amplified for 12 cycles and cleaned up with SPRIselect beads (Beckman, Indianapolis, IN, USA). These samples were then diluted at 4:1 and analyzed in a Bioanalyzer (Agilent Technologies, Santa Clara, CA, USA) to determine cDNA concentration. The construction of the cDNA libraries was accomplished according to the Single Cell 3' Reagent Kits v2 user guide with appropriate modifications to the PCR cycles based on the calculated cDNA concentration (as recommended by 10x Genomics).

The molarity of each library was calculated based on the library size as measured by the Bioanalyzer (Agilent Technologies) and qPCR amplification data. The samples were then pooled together, normalized to 10 nM and diluted to 2 nM using the elution buffer containing 0.1% Tween-20 (Sigma-Aldrich). Next, the samples were sequenced using the Novaseq 6000 (Illumina, San Diego, CA, USA) under the following run parameters: read 1, 150 cycles; read 2, 150 cycles; index 1, 8 cycles. A median sequencing depth of 50,000 reads/cell was used for each sample.

*(iii) Quality control and data analysis*

The raw DNA sequencing data (bcl files) were converted to fastq files using the Illumina bcl2fastq (version 2.19.1; Illumina) and then aligned to the human genome reference sequence (GRCh38). Next, the CellRanger (version 2.2.0; 10x Genomics Inc.) pipeline was performed to generate a digital gene expression matrix for each piece of data. However, the dataset was removed if the data captured fewer than 300 unique genes, or the data contained greater than 10% mitochondrial reads. Next, the raw digital gene expression matrix (UMI counts per gene per cell) was filtered, normalized, integrated and clustered using the Seurat R package (version 2.4). The clusters were visualized using t-distributed stochastic neighbor embedding (t-SNE). For each cluster, the Wilcoxon rank-sum test was performed to assess genes that were differentially expressed.

Furthermore, the cell clusters were also annotated based on expression of curated known cell markers (Table S4). For example, clusters with epithelial cell markers (EPCAM, VIL1, CLDN7 and CDH1) and proliferative cell markers (MKI67, BIRC5 and CDK1) were used for sub-clustering between GC and normal epithelial cells. In addition, the Kruskal-Wallis test was used to analyze the level of MYH9 mRNA among tissues from gastric epithelium (N), gastric primary tumor (PT) and peritoneal metastasis of gastric tumor (MT) using the single-cell transcriptome data, while the Pearson's correlation analysis was performed to assess the association of MYH9 mRNA with CTNNB1 (β-catenin) mRNA or β-catenin-induced target mRNAs in derived from the gastric epithelium (gastric epithelial cells and GC cells) using the single-cell transcriptome data.

**Dataset and survival analysis stratified by MYH9 expression**

The transcriptome data on GC tissues were downloaded from the TCGA database. The differential MYH9 expression between GC and normal tissues was analyzed using tools from the TCGA and GENT websites (http://medical-genome.kribb.re.kr/GENT/search/view_result.php). The association of MYH9 mRNA level with survival of GC patients was analyzed using tools from the TCGA and KMplot websites (http://kmplot.com), while the association of MYH9 with CTNNB1 mRNA levels was assessed using the GEPIA website (http://gepia.cancer-pku.cn/index.html).

**MYH9 3D structure modeling and DNA-protein docking analysis**

To determine the interaction between MYH9 protein and CTNNB1-P, the amino acid sequences of MYH9 were retrieved from NCBI (https://www.ncbi.nlm.nih.gov/), while the 3D structure of MYH9 protein was obtained from Modeller (http://salilab.org/modeller/) as reported in a previous study [[2](#_ENREF_2)]. The MYH9 potential DBDs were analyzed using an online tool from the PSORT II Prediction program (http://www.genscript.com/cgi-bin/tools/psort2.pl). To evaluate the MYH9 protein modeling, the Ramachandran plot was generated using tools from the PROCHECK (http://servicesn.mbi.ucla.edu/PROCHECK/) program, while the ZDOCK server (http://zdock.umassmed.edu/) was used to dock DNA and protein, and Pymol (Version 1.8) molecular graphics software was used to analyze DNA-protein interaction.

**RNA isolation and qPCR**

TRIzol reagent (Cat. #15596026; Invitrogen, Carlsbad, CA, USA) was used to isolate total cellular RNAs from gastric tissues or cells, while the PrismScript RT Reagent Kit (Cat. #RR036A; TaKaRa, Dalian, China) was utilized to generate cDNA with 1 μg RNA according to the manufacturer’s instructions. qPCR was performed using SYBR Premix Ex Taq (Cat. #RR820A; TaKaRa) in the LightCycler 480 system (Roche, Basel, Switzerland). The primers are listed in Table S3. The level of GAPDH mRNA was used as an internal control and the relative level of each mRNA expression was analyzed using the 2^−ΔΔCt^ method [[3](#_ENREF_3)].

**Co-IP and western blot**

For western blot analysis, cell lysates were prepared using the mammalian protein extraction kit (Cat. #CW0889; CWBIO, Beijing, China), while tissue lysates were prepared using the tissue protein extraction kit (Cat. #CW0891; CWBIO). For co-IP, cell lysates were prepared using IP lysis buffer containing 20 mM Tris-HCl at pH 7.5, 150 mM NaCl, 1 mM EDTA, 1 mM EGTA, 1% Triton X-100, 2.5 mM sodium pyrophosphate, 1 mM β-glycerolphosphate, 1 mM Na_3_VO_4_ and 1 μg/mL leupeptin. The cell nuclear extracts were obtained using NE-PER™ nuclear and cytoplasmic extraction reagents (Cat. #78835; Thermo Scientific, Waltham, MA, USA). The protein concentration was determined using the bicinchoninic acid (BCA) protein assay kit (Cat. #CW0014S; CWBIO).

The western blot procedures were performed according to a standard protocol from our laboratory. Specifically, protein samples were separated on 8% sodium dodecyl sulfate-polyacrylamide gel electrophoresis (SDS-PAGE) gels and transferred onto nitrocellulose membranes (Bio-Rad, Hercules, CA, USA). The membranes were first blocked in 5% bovine serum albumin (BSA) in Tris-based saline-Tween 20 at room temperature for 1 h and then incubated with the appropriate primary antibody (see the Key Resources Table). An anti-phosphorylated MYH9 S1916 primary antibody was generated using the CDAMNREVS-pS-LKNKLRRG peptides with the help of BBI Life Sciences (Guangzhou, China). Subsequently, these membranes were incubated with the appropriate secondary antibody. The positive protein signal was visualized with the Immobilon-Western HRP kit (Cat. #WBKLS0500; Millipore, Burlington, MA, USA) and exposed to x-ray films.

For co-IP followed by western blot, the protein lysates were first pre-cleared by incubation with protein G sepharose at 4 °C for 2 h and immunoprecipitated at 4 °C overnight with 2.5 μg of each indicated antibody. The next day, 100 μL of 50% protein G sepharose slurry was added to the reaction mixture and incubated at 4 °C for 2 h. Next, the mixture was washed five times with a lysis buffer through centrifugation, and the precipitates were re-suspended in 35 μL SDS sample buffer, heated to 100 °C for 10 min, and then subjected to western blot.

**Construction of TALEN-mediated somatic MYH9 KO vectors**

To specifically KO MYH9 in GC cells, six TALEN binding pairs were selected from MYH9 CDs in the second exon (Figure 2B) according to previous studies [[4](#_ENREF_4), [5](#_ENREF_5)]. The genome recognition sequences of the left and right TALEN arms are shown in Table S6. After PCR amplification, the vectors carrying the left and right TALEN arms, namely TALEN-MYH9-Ls and TALEN-MYH9-Rs, respectively, were generated using a one-step ligation with the FastTALE^TM^ TALEN assembly kit (SIDANSAI Biotechnology, Shanghai, China) according to the manufacturer’s protocol (Figure S7A). After DNA sequencing confirmation, these six pairs of TALEN-MYH9 vectors were respectively transfected into MGC80-3 and AGS cells, which were then selected with puromycin. The MYH9 deletion clones were confirmed by PCR and DNA sequencing (Figure S7B). The vector pair of TALEN-MYH9-L2 and TALEN-MYH9-R3 was confirmed to be the most effective pair and selected for subsequent experiments.

**Vector construction**

The reporter vectors were constructed using the basic pGL3 reporter plasmid (Promega, Madison, WI, USA) to detect and confirm MYH9 binding to the CTNNB1 promoter. In brief, different lengths of the CTNNB1 promoter were selected, PCR-amplified, and subcloned into pGL3 (Figure S9A). The primers used in this study were P1 (5'-CGGGGTACCAGGAATCAGTACCTGAAAACGCC-3' for -1686/+270), P2 (5'-CGGGGTACCACAGCCTTCGTGAGTGG-3' for -771/+270), P3 (5'-CGGGGTACC AACTTCCGCCCTCCCAG-3' for -444/+270), P4 (5'-CGGGGTACCTCAGACGGCAGCAGACT-3' for -110/+270), P5 (5'-CGGGGTACCATACAGCGGCTTCTGC-3' for +3/+270), P6 (5'-CGGGGTACC CGCCATTTTAAGCCTCTC-3' for +45/+270), P7 (5'-CGGGGTACC CTGAGGAGCAGCTTCAGT-3' for +132/+270), and a common antisense primer P8 (5'-CCCAAGCTTAGAGGAAGCGGGAGCAG-3'). The amplified DNA fragments of the CTNNB1 promoter included *Kpn* I and *Hind* III sites at the 5'- and 3'-ends, respectively. Furthermore, the CTNNB1 promoter constructs with the mutant MYH9 binding sites were generated using the GeneTailor™ site-directed mutagenesis system (Cat. #12397-022; Invitrogen) (Figure S4E) and confirmed with DNA sequencing.

Furthermore, plasmids with MYH9, β-actin or MYL9 cDNA were obtained from GeneCopoeia (Cat. Nos. #EX-T1335-M98-5, #EX-T1780-M98 and #EX-A4086-M98, respectively) and shown in the Key Resources Table. The MYH9 plasmids with mutations in potential DBDs or phosphorylation sites were generated using the GeneTailor™ site-directed mutagenesis system (Cat. #12397-022; Invitrogen) and shown in Table S10. The key bases within potential DBDs and the special bases (serine (S) or threonine (T)) from the five well-known phosphorylation sites (T1800, S1803, S1808, S1916 and S1943) within the MYH9 gene were all replaced with glycine (Gly).

In addition, the functional significance of four putative NLSs (Table S11) in MYH9 was also assessed. In brief, 15 MYH9 NLS mutation plasmids were constructed by replacing lysine or arginine residues with alanine, and subcloned into the MYH9 plasmid (Cat. #EX-T1335-M98-5; GeneCopoeia) using the GeneTailor™ site-directed mutagenesis system (Cat. #12397-022; Invitrogen), which were designated as M'1–15. These four putative NLSs were mutated from 5'-AAGAAGAGGCAC-3' (between 421 bp and 432 bp in MYH9 gene) to 5'-GCCGCCGCCCAC-3' in the M'1 plasmid, from 5'-CACAAGCGCAAG-3' (between 3733 bp and 3744 bp) to 5'-GCGGCCGCCGCC-3' in the M'2 plasmid, from 5'-AAGCGCAAGAAA-3' (between 3736 bp and 3747 bp) to 5'-GCCGCCGCCGCC-3' in the M'3 plasmid, and from 5'-CGCAGGGAGGAGAAGCAGCGACAGGAGCTGGAGAAGACCCGCCGGAAGCTG-3' (between 3109 bp and 3159 bp) to 5'-GCCGCCGAGGAGGCCCAGGCCCAGGAGCTGGAGGCCACCGCCGCCGCCCTG-3' in the M'4 plasmid. In addition, the M'5 to M'15 plasmids were constructed with combinations of these four mutations (Table S12).

**Cell transfection**

Cells were plated into 6-well plates at a density of 5x10^4^ cells/well, grown overnight, and then transfected with plasmids using Lipofectamine 3000 (Cat. #L3000015; Invitrogen) according to the manufacturer’s instructions. Next, the cells were cultured and selected with puromycin (Cat. #P7255; Sigma, St. Louis, MO, USA) for further study.

To generate MYH9-knockdown cells, GC cells were infected with a lentivirus carrying MYH9 or NC shRNA (GenePharma, Suzhou, China; Table S5). Next, the cells were grown and selected in growth media containing 2 μg/mL puromycin. The puromycin-resistant colonies were then selected, pooled and cultured for further experiments under puromycin-selection conditions.

**Immunofluorescence**

Cancer cell lines (e.g., MGC 80-3, AGS and Caco-2) grown on glass-bottom cell culture dishes or frozen human gastric tissue sections were fixed in 4% paraformaldehyde, permeabilized in 0.1% Triton X-100 and blocked in 4% BSA. Next, cells or tissue sections were incubated with a mouse- or rabbit-derived primary antibody (details in the Key Resources Table), followed by incubation with an anti-rabbit IgG (H+L), F(ab')2 fragment conjugated with Alexa Fluor® 555 (red), anti-rabbit IgG (H+L), F(ab')2 fragment conjugated with Alexa Fluor® 488 (green), anti-mouse IgG (H+L), F(ab')2 fragment conjugated with Alexa Fluor® 488 (green) or anti-mouse IgG (H+L), F(ab')2 fragment conjugated with Alexa Fluor® 555 (red). Subsequently, they were incubated with 4,6-diamidino-2-phenylindole (DAPI) to visualize the cellular DNA. To detect the microfilament morphology, the cells or tissue sections were incubated with FITC-conjugated phalloidin. These immunostained cells or tissue sections were reviewed and scored under an Olympus FV300 laser-scanning confocal microscope (Olympus America Inc., Melville, NY, USA).

**Transmission electron microscopy**

GC cell lines were collected from the cell culture dishes and fixed in 3% paraformaldehyde with 0.1% glutaraldehyde in Sorenson buffer (SB; 0.1 M Na/K phosphate buffer at pH 7.3) at 4 °C overnight. Next, the samples were washed with SB through centrifugation, placed in 1% low-melting agarose in SB, and embedded in LR White (Polysciences; polymerized under UV light at 4 °C for 48 h) according to previous studies [[6](#_ENREF_6), [7](#_ENREF_7)]. The blocks were sectioned into 80 nm thick ultrathin sections and subsequently incubated at room temperature for 45 min with a rabbit monoclonal IgG to MYH9 (sc-98978; Santa Cruz Biotechnology, Dallas, TX, USA) at a 1:100 dilution and then with a 10 nm gold-labeled goat anti-rabbit IgG (Cat. #G7402; Sigma-Aldrich). Next, these ultrathin sections were contrasted with saturated uranyl acetate and reviewed under a Philips Morgagni electron microscope equipped with a CCD Mega View II camera (SIS, Germany).

**Luciferase reporter gene assay**

GC cells (1x10^5^) were seeded into 6-well plates, grown overnight, and then co-transfected with MYH9 plasmid and full-length or truncated CTNNB1 promoter constructs using Lipofectamine 3000 (Cat. #L3000015; Invitrogen) for 24 h according to the protocol provided by the manufacturer, while the renilla luciferase reporter plasmid pR-TK (Cat. #E2241; Promega) was used as a control. Furthermore, to assess the MYH9-mediated CTNNB1 transcription in activation of the Wnt/β-catenin signaling pathway, the TOP/FOP-flash assay was used by transfecting GC cells with TOP-flash (the TCF reporter plasmid; Cat. #21-170; Merck Millipore) or FOP-flash (plasmid carrying the mutant TCF binding sites; Cat. #21-169; Merck-Millipore) using Lipofectamine 3000 for 24 h, while the renilla luciferase reporter plasmid pR-TK was again used as the control. Next, these transfected cells were harvested and lysed for extraction of total cellular protein and then subjected to a luciferase assay. The luciferase activity was measured by using the Promega Dual-Glo® luciferase assay system (Cat. #E2940) and the Turner BioSystems 20/20n Luminometer according to the manufacturer’s instructions. The experiments were performed in triplicate and repeated three times.

**ChIP assay**

The ChIP assay was performed in MGC 80-3 and AGS cells using the Pierce^TM^ agarose ChIP kit (Cat. #26156; Thermo Scientific) according to the manufacturer’s protocol, and IP was performed using the appropriate primary antibody listed in the Key Resources Tables. The ChIP-yielded DNA was amplified with PCR using primers for the core CTNNB1 promoter (core CTNNB1-P), i.e., 5'-CGGGGTACCTCAGACGGCAGCAGACT-3' and 5'-CCCAAGCTTGAGAGGCTTAAAATGGCG-3'. The anti-RNAPII antibody and GAPDH PCR primers were used as the positive controls for ChIP and PCR amplification, respectively. Normal rabbit IgG was used as a negative control.

**Chromatin fractionation**

Chromatin fractions were prepared as previously described [[8](#_ENREF_8)]. In particular, GC cell lines were washed with phosphate-buffered saline (PBS), resuspended in 2 mL chromatin fractionation buffer (0.15 M NaCl, 10 mM MgCl_2_, 10 mM CaCl_2_, 1 mM PMSF, 15 mM Tris at pH 7.5 and 0.1% Tween 20), and ruptured using Ultra-Turrax in 0.1% NP-10. After centrifugation (800 x *g*) at 4 °C for 10 min, the cell nuclei were collected and digested with DNase I (0.2 μg/L) at 30 °C for 10 min and centrifuged to pellet. The cell pellet was re-suspended in chromatin fractionation buffer, NaCl was added to reach a final concentration of 0.4 M NaCl, and then samples were incubated at 4 °C for 30 min and centrifuged (21,000 x *g*) for 10 min. The supernatant was collected and saved as 0.4 M chromatin fraction. Next, the 0.8 M chromatin fraction was prepared similarly by adding NaCl to a final concentration of 0.8 M, while the final pellet was saved as the residual pellet. These 0.4 M and 0.8 M chromatin fraction samples as well as the residual pellet were subjected to western blot analysis.

**Agarose-oligonucleotide pull-down assay**

DNA affinity purification was performed as previously described [[8](#_ENREF_8)]. The oligonucleotides for the human *CTNNB1* promoter and their complementary strands were synthesized by Sigma Genosys (Woodlands, TX, USA) and biotinylated using Pierce™ Biotin 3' End DNA Labeling Kit (Cat. #89818; Thermo Scientific) (details in Table S8). These oligonucleotides were annealed to form double stranded oligonucleotides, which were then incubated with streptavidin-conjugated agarose beads at 4 °C for 60 min and washed twice with IP lysis buffer. Next, the nuclear extract (50 μg each) in 200 μL IP lysis buffer was pre-cleared with agarose beads at 4 °C for 90 min to reduce any nonspecific binding, and then incubated with oligo/streptavidin-conjugated beads at 4 °C overnight. The mixtures were washed five times with IP lysis buffer through centrifugation the following day, and the affinity-purified proteins were eluted by boiling in SDS sample buffer for 5 min. The samples were then subjected to analysis by western blot.

**Drug stimulation experiments**

The recombinant human Wnt3a protein (Cat. #5036-WN-500; R&D Systems) was reconstituted in PBS containing 0.1% BSA to reach a final concentration of 200 μg/ml. According to a previous study [[9](#_ENREF_9)] and our preliminary data (not shown), 20 ng/mL Wnt3a protein was the optimal concentration for our experiment. According to the data from Figure S16A, the level of β-catenin in the nuclei was increased substantially after Wnt3a addition in a time-dependent manner, and an 8 h treatment was selected for the luciferase reporter gene assay.

To explore the effects of drugs on the inhibition of nuclear MYH9-mediated CTNNB1 transcription, our experiments used GC cell lines to investigate blebbistatin (Cat. #S7099; Selleck), ML-7 hydrochloride (Cat. #HY-15417; MCE), STS (Cat. #HY-15141; MCE), Y-27632 dihydrochloride (Cat. #HY-10583; MCE), CX-4945 (Cat. #HY-50855; MCE) and Mycrocystin LR standards (MCLR; Cat. #ALX-350-012-C050; Enzo Life Sciences). The IC_50_ value for each drug in GC cells was assessed using the cell viability cell counting kit-8 (CCK-8) assay (Cat. #BB-4202-2; BestBio).

**Flow cytometry cell cycle and apoptosis assay**

To assess cell cycle distribution, the experimental cells (1 x 10^6^) were trypsinized, re-suspended to generate single-cell suspensions, fixed in 70% ethanol at 4 °C overnight, and then stained with propidium iodide from the Cell Cycle Detection Kit (Cat. #KGA512; KEYGEN, Nanjing, China) according to the manufacturer’s instruction. Next, the cells were analyzed with a FACScan flow cytometer (BD Biosciences, San Jose, CA, USA) and quantified using ModFit 3.0 software (Verity Software House, Topsham, ME, USA).

Furthermore, to determine the level of apoptosis, 1 x 10^6^ cells were collected and stained with the Annexin V-APC/7-AAD apoptosis kit (Cat. #AP105-30; MultiSciences Biotech, Shanghai, China) following the manufacturer's protocol, analyzed with a FACScan flow cytometer (BD Biosciences), and quantified using FlowJo software (Tree Star, Inc., Ashland, OR, USA).

**Soft agar cell growth assay**

For the soft agar assay, 1 mL of 0.5% low melting agarose (Cat. #10214ES08; YEASEN, Shanghai, China) in DMEM supplemented with 10% fetal calf serum (FCS) was poured into each well of a 12-well plate. After the agar had solidified, cells (1 x 10^4^) were mixed with 1 mL of 0.3% low melting agarose in DMEM containing 10% FCS and placed on top of the agar. Cell were then grown at 37 °C in 5% CO_2_ for 21 days. The cell growth media was refreshed every two days. Next, the cell colonies were stained with 0.5% crystal violet and counted under an [inverted](javascript:;) fluorescence microscope. The number of cell clones per well and the mean volume per clone were recorded and analyzed.

**Cell anoikis assay**

Cells undergoing anoikis were identified and quantified using the CytoSelect 24-well anoikis assay kit (Cat. #CBA-080; Cell BioLabs) according to the manufacturer's protocol. Briefly, cells (5 x 10^5^) in DMEM supplemented with 10% FCS were seeded into poly-HEMA coated plates, which prevented cells from adhering to the bottom of the plates thereby mimicking anchorage independent growth conditions, and grown for 48 h. Then, the cells were subjected to a cell viability MTT assay or the Calcein AM/EthD-1 fluorometric detection assay according to the manufacturers' protocols. The data were summarized as percent of control.

***In vivo* experiments**

*(i) Nude mouse models*

The animal protocols of this study were approved by the Institutional Animal Care and Use Committee (IACUC) of Nanfang Hospital (Guangzhou, China), and followed the Guidelines of the Care and Use of Laboratory Animals issued by the Chinese Council on Animal Research. Female athymic BALB/c nu/nu mice at 4 to 5 weeks of age were purchased from the Central Laboratory of Animal Science at Southern Medical University (Guangzhou, China) and maintained in specific-pathogen-free cages with a standard diet, constant temperature, constant humidity and with standard light/dark cycles. To assess the maximum tolerated doses (MTDs) of STS *in vivo*, dose escalating experiments were performed in 5-week-old female BALB/c nu/nu mice according to a previous study [[10](#_ENREF_10)]. Briefly, STS was dissolved in acetate buffer (pH 5.0) containing 1% dimethyl sulfoxide (DMSO; v/v%) and intraperitoneally injected (i.p.) into the experimental mice daily for five days. These mice were randomly assigned into four groups (named groups A through D) for different STS dosages (Table S14). The health status of each mouse (such as body weight, physical activity, appearance, breath and posture) was monitored and recorded. The mice that reached humane endpoints were euthanized using cervical dislocation.

Furthermore, an additional 20 female nude mice were used to establish the orthotropic xenograft GC mouse model according to our previous study [[3](#_ENREF_3)]. Two weeks after establishment of the model, the mice were randomly assigned into STS or control groups (n = 10/group) and then treated daily with i.p. injections of STS (0.6 mg/kg/day) or the same amount of DMSO for five consecutive days. The body weight was monitored every two days for six weeks and the mice were then euthanized. The tumorigenesis in mice was observed by using the multi-functional *in vivo* imaging system (MIIS; Molecular Devices, Sunnyvale, CA, USA). Tumor diameters were measured using calipers, and the tumor volumes were calculated according to the formula (0.5 x length x width^2^). The primary tumors and metastatic nodules were analyzed by qPCR or resected for hematoxylin and eosin (H&E) staining.

To assess the MYH9-mediated CTNNB1 expression in the anoikis resistance of GC cells, a model of GC metastasis was established. Specifically, 40 nude mice were randomly assigned into one of four groups (NC + vector, shRNA3 + vector, shRNA3 + MYH9, shRNA3 + CTNNB1; n = 10/group) and injected with tumor cell suspensions (1 x 10^6^ cells/100 µL/mouse) into the lateral tail vein of each anesthetized nude mouse. Eight weeks after injection, the mice were sacrificed, and tumorigenesis was assessed using the multi-functional *in vivo* imaging system (MIIS; Molecular Devices). The metastatic nodules were resected for H&E staining.

*(ii) Transgenic mouse models*

LSL-hMYH9 transgenic and Myh9^fl/fl^ transgenic mice were obtained from Cyagen Biosciences Inc. (Guangzhou, China), while the Tff1-knockout mice were purchased from the Wellcome Trust Sanger Institute Mouse Genetics Project (Sanger MGP) with the help of Vital River Lab Animal Technology Co., Ltd (Beijing, China). The ATP4b-cre mouse strain was kindly provided by Dr. Xiao Yang of the State Key Laboratory of Proteomics, Genetic Laboratory of Development and Disease, Institute of Biotechnology (Beijing, China) [[11-13](#_ENREF_11)]. Genotyping of these transgenic mice was performed using PCR analysis of the tail-tip genomic DNA with the Mouse Direct PCR Kit (Cat. #B40015; Biotool, Houston, TX, USA). The PCR primer sequences are shown in Table S13. These mice were housed individually in ventilated cages under specific pathogen-free conditions and fed with a standard diet. The mice were propagated on a C57BL/6 genetic background. Age- and sex-matched littermates were used in all experiments.

Transgenic mice were earmarked and randomly assigned to an experimental group by an independent researcher. For the survival analysis, age- and sex-matched mice were housed together. The activity of the mice was monitored every two days, and the body weight was recorded once per week. For STS treatment of LSL-hMYH9; ATP4b-cre; Tff^-/-^ mice, a dose escalating study was performed in 4-week-old male C57BL/6 mice. The results confirmed that the STS therapeutic regimen used in nude mice (i.p. injection of 0.6 mg/kg/day for five consecutive days) was also optimal for transgenic mice (Table S14). Due to the length of the experiments (usually more than 1 year), an additional 15 4-week-old male C57BL/6 mice were randomly assigned into three groups (A–C groups) (Table S14). Mice in group A were treated with STS (i.p. injection of 0.6 mg/kg/day for five consecutive days every month). Mice in group B were treated with same STS dosage for five consecutive days every two months, and mice in group C were treated every three months. The experiment continued for 14 months and the results indicated that an i.p. injection of 0.6 mg STS/kg/day for five consecutive days every two months was the optimal condition (Table S14). Next, 20 4-week-old male hMYH9 transgenic mice (LSL-hMYH9; ATP4b-cre; Tff^-/-^) were randomly assigned into two groups (STS vs. DMSO group; n = 10/group). The mice were treated for 14 months (0.6 mg STS/kg/day for five consecutive days by i.p. injection every 2 months). The mice were euthanized by cervical dislocation at the end of the experiment, and stomach tissues were collected for analysis with qPCR and H&E staining.

**Supplementary data**

Supplemental data include 22 figures and 14 tables, and are retrievable online.


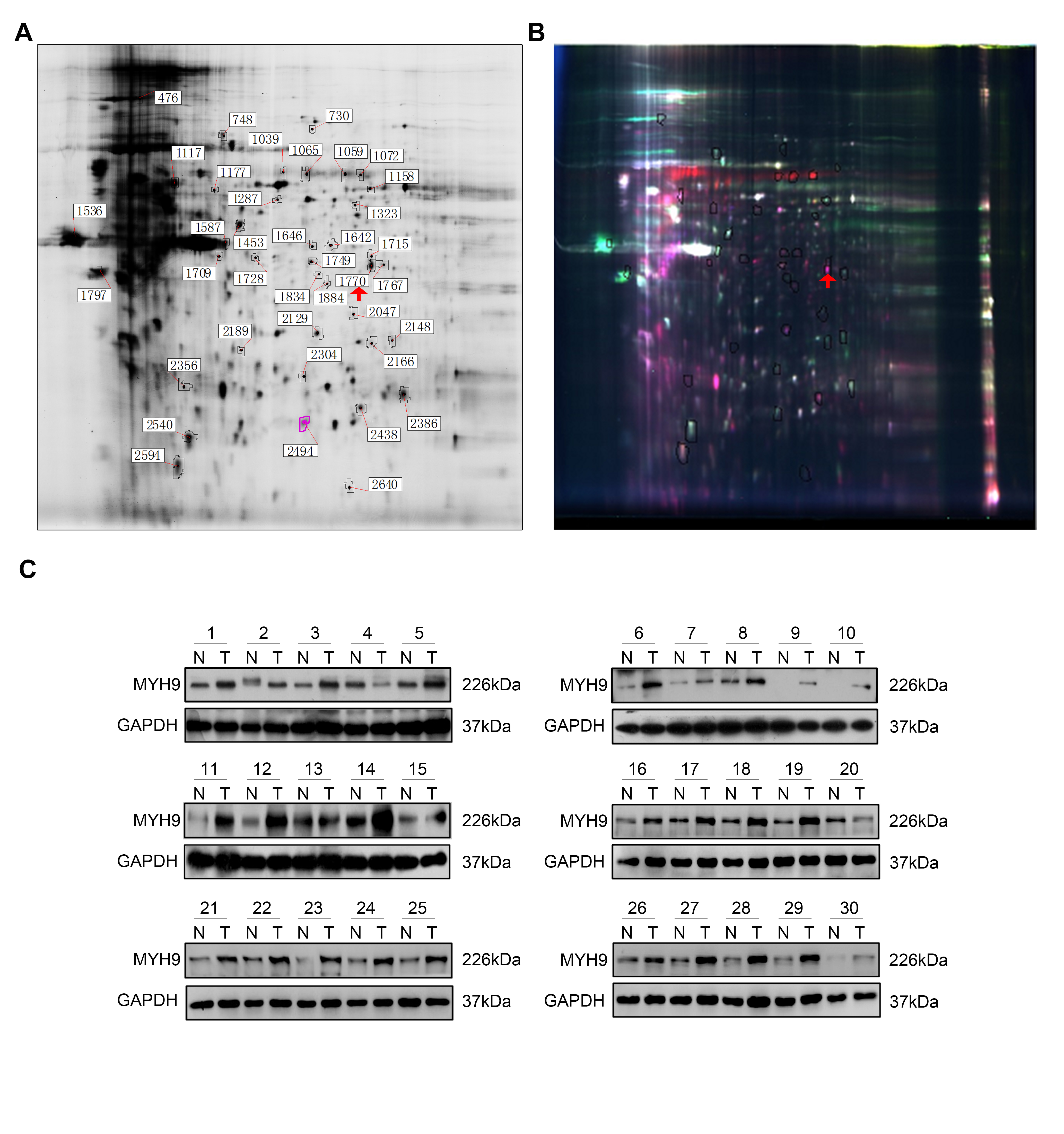


**Figure S1. MYH9 protein was upregulated in human GC tissues**

(A) Location of 39 selected protein spots in the 2D gel. Spot numbers correspond to those in Table S2. (B) The position of protein spots of interest in the 2-DIGE image. All the DEPs were assessed using MALDI-TOF/TOF MS. The MYH9 protein was marked with a red arrow. (C) Tissue samples from 30 paired GC (T) and normal gastric mucosae (N) were collected for western blot analysis of MYH9 protein levels in GC tissues.


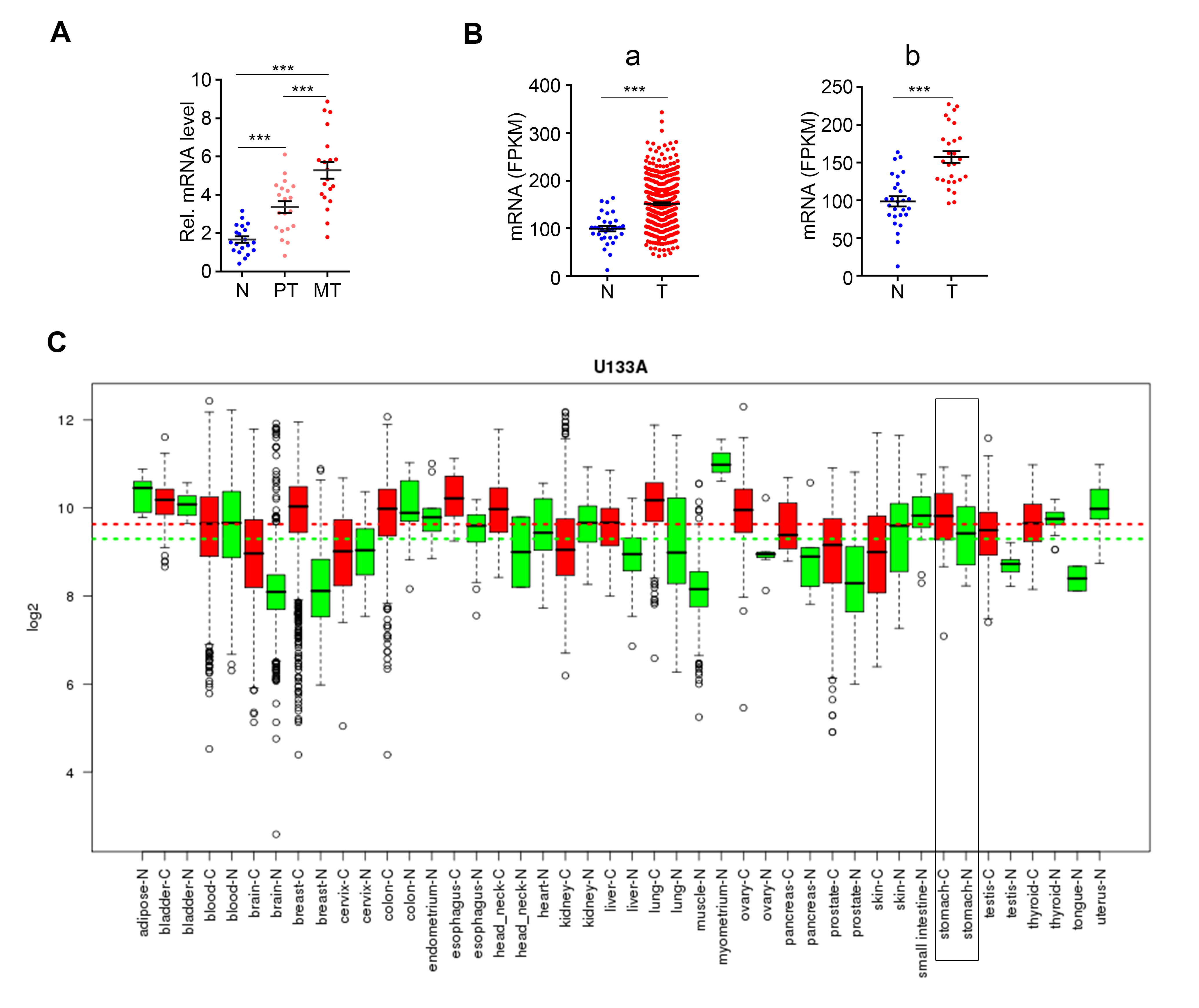


**Figure S2. MYH9 mRNA was significantly upregulated in human GC tissues**

(A) Tissues from 19 advanced GC patients were subjected to qPCR analysis of MYH9 mRNA. N, normal gastric mucosae; T, primary GC tissues; M, peritoneal metastases. (B) TCGA data analysis for MYH9 mRNA expression in GC tissues (cancer vs. normal mucosae; a, unpaired; b, paired). (C) The GENT dataset analysis for MYH9 mRNA expression in cancers.


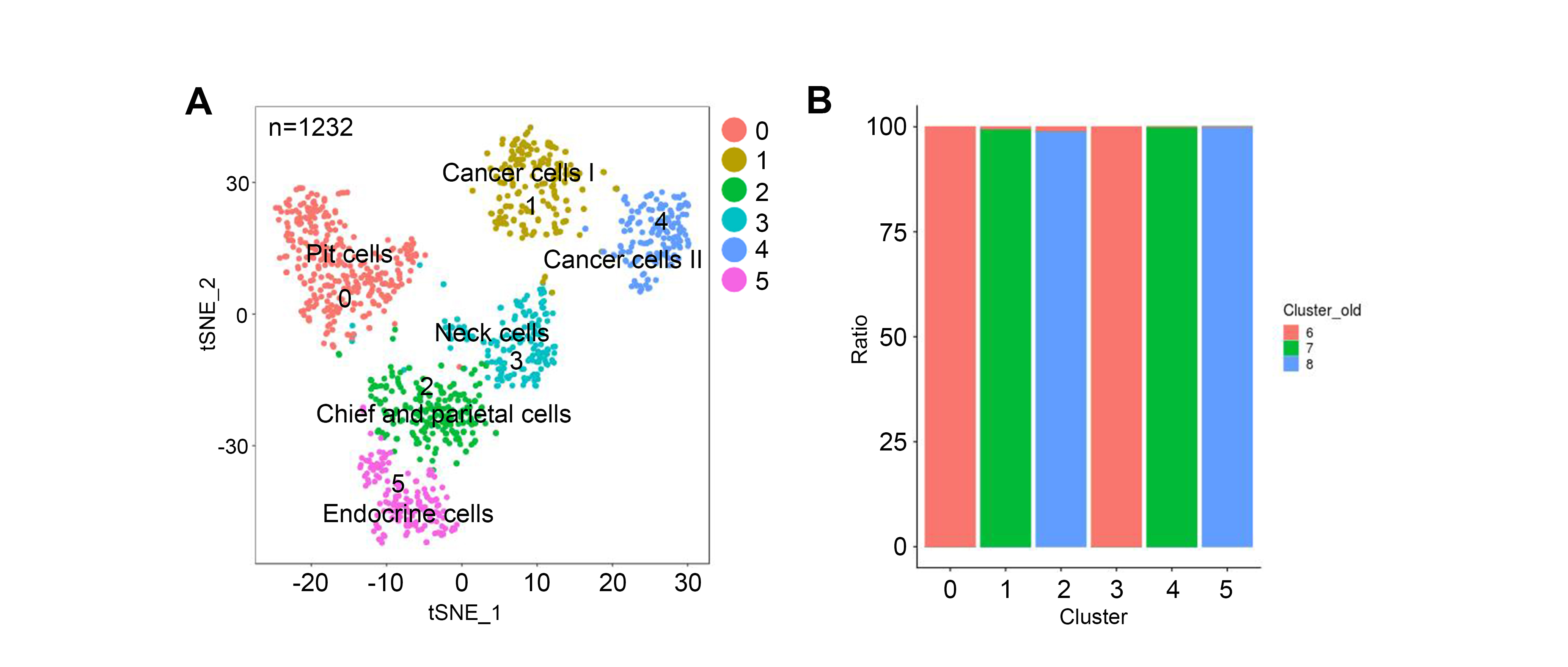


**Figure S3. Sub-cluster analysis of epithelium-derived cells**

(A) The t-SNE plot of 1232 epithelium-derived cells for sub-clustering and visualization of cell-type clusters based on expression of known marker genes (see also Table S7). (B) The proportion of epithelium-derived cells (cluster 6, 7 and 8 from Figure 1B) in the six clusters from the sub-cluster analysis.


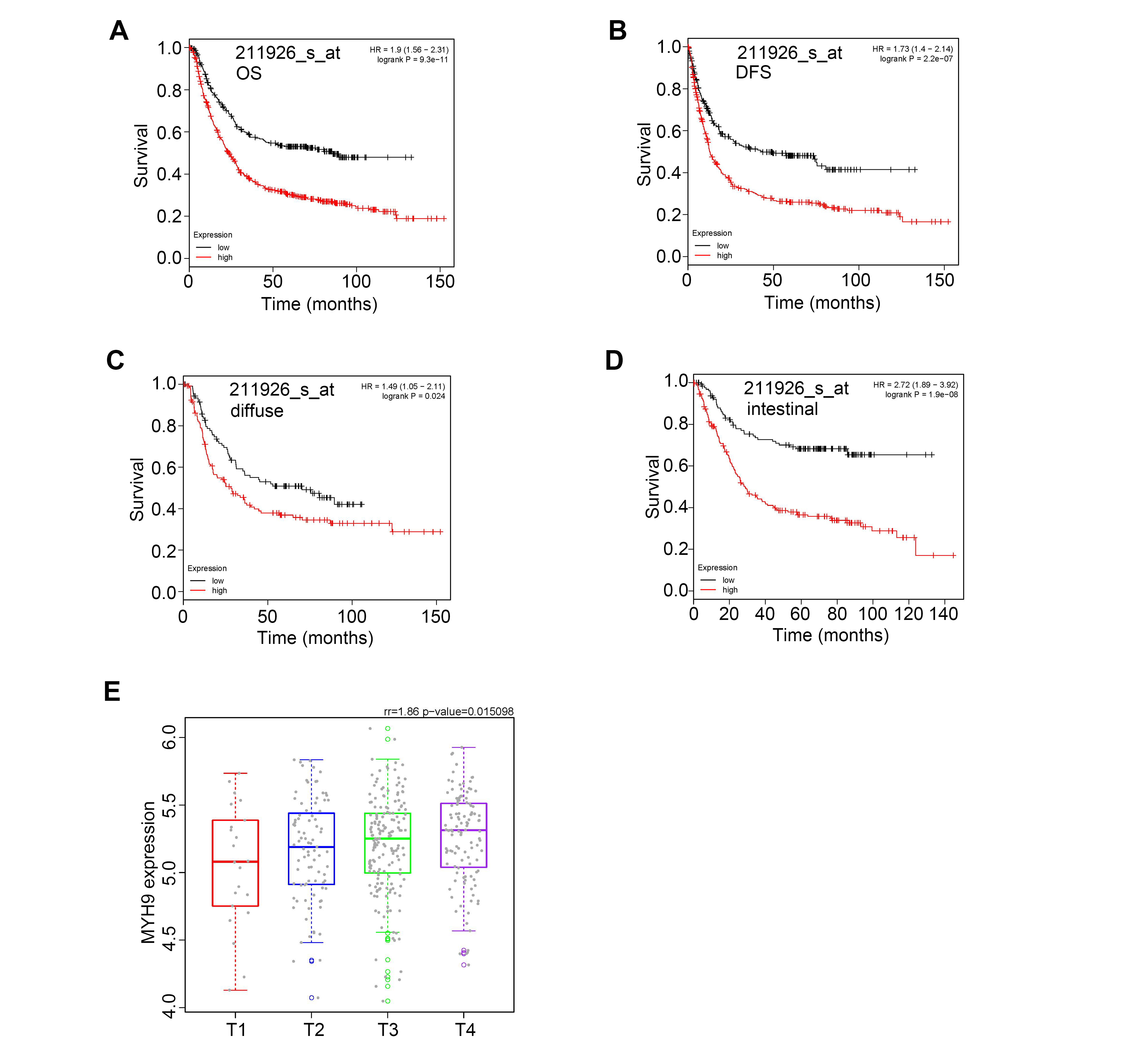


**Figure S4. The overexpression of MYH9 mRNA was associated with poor survival of GC patients and advanced pT stage of GC**

(A-D) Kaplan-Meier curves of the dataset from KMplot (http://kmplot.com). The level of MYH9 mRNA was inversely associated with overall survival (OS; A) and disease-free survival (DFS; B) of GC patients. It also was inversely associated with OS of diffuse (C) or intestinal (D) metastasis in GC patients. (E) Association of MYH9 mRNA with advanced pT stage of GC based on TCGA data.


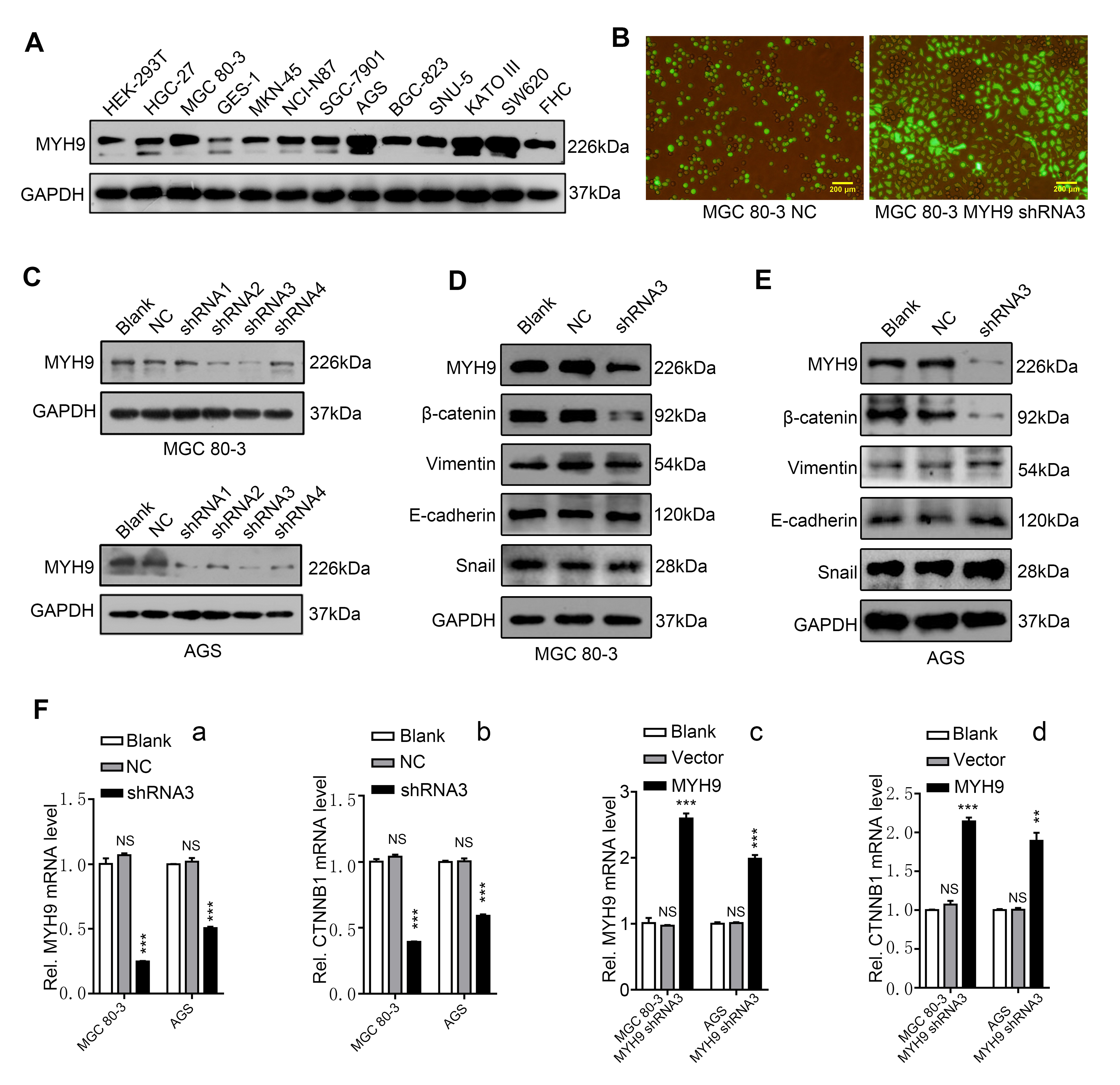


**Figure S5. Downregulation of MYH9 expression by transfecting GC cells with MYH9 shRNA promotes a change in cell morphology and leads to downregulation of β-catenin protein expression in GC cell lines**

(A) The MYH9 protein levels in human gastric epithelial GES-1 cell line, human embryonic kidney HEK-293T cell line, human intestinal epithelial FHC cell line, human gastric cancer cell lines (HGC-27, MGC 80-3, MKN-45, NCI-N87, SGC-7901, AGS, BGC-823, SNU-5 and KATO-III) and human colon cancer cell line SW620 were detected by western blot. MGC 80-3 and AGS were chosen for further study due to their relatively high levels of MYH9 protein. (B) MGC 80-3 cell line transfected with MYH9 shRNA3 were digested with trypsin for 1 min and then observed under the fluorescence microscope (200x). MGC 80-3 NC was used as a control. MYH9 shRNA3-infected cells had reduced cell contractility during trypsin digestion, which supported MYH9 as a cytoskeletal protein that is associated with cell contraction. (C) The MYH9 protein levels in MGC 80-3 and AGS cells transfected with MYH9 shRNAs were detected by western blot. The cells transfected with shRNA3 were chosen for this study. Next, the protein levels of MYH9 and EMT-associated proteins in MGC 80-3 (D) and AGS (E) cells transfected with MYH9 shRNA3 were detected by western blot. (F) The MYH9 mRNA (a) and CTNNB1 (b) mRNA levels in MGC 80-3 and AGS cells transfected with MYH9 shRNAs were detected by qPCR. Next, these MYH9 knockdown cells were transfected with MYH9 plasmids and also subjected to qPCR analysis of MYH9 (c) and CTNNB1 (d) mRNA.


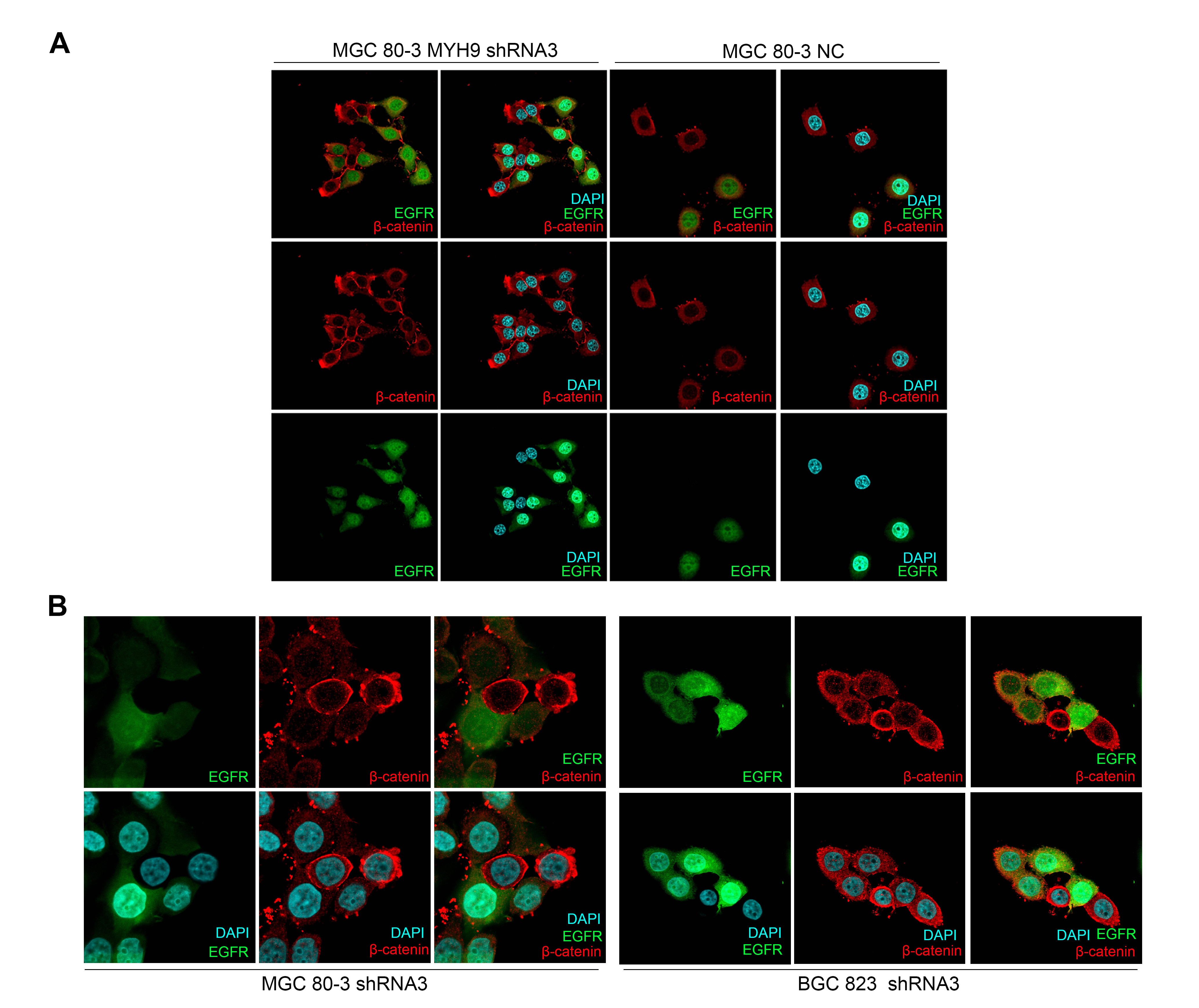


**Figure S6. The protein level of β-catenin decreases in GC cells with MYH9 downregulation**

(A) Immunofluorescence. β-catenin protein (red) in MGC 80-3 cell lines transfected with MYH9 shRNA3 and NC (green) was detected by fluorescence microscopy. (B) Confocal microscopy. β-catenin protein (red) in MGC 80-3 and BGC 823 cell lines infected with MYH9 shRNA3 (green) was detected by confocal microscopy. DAPI was used to stain the nucleus.


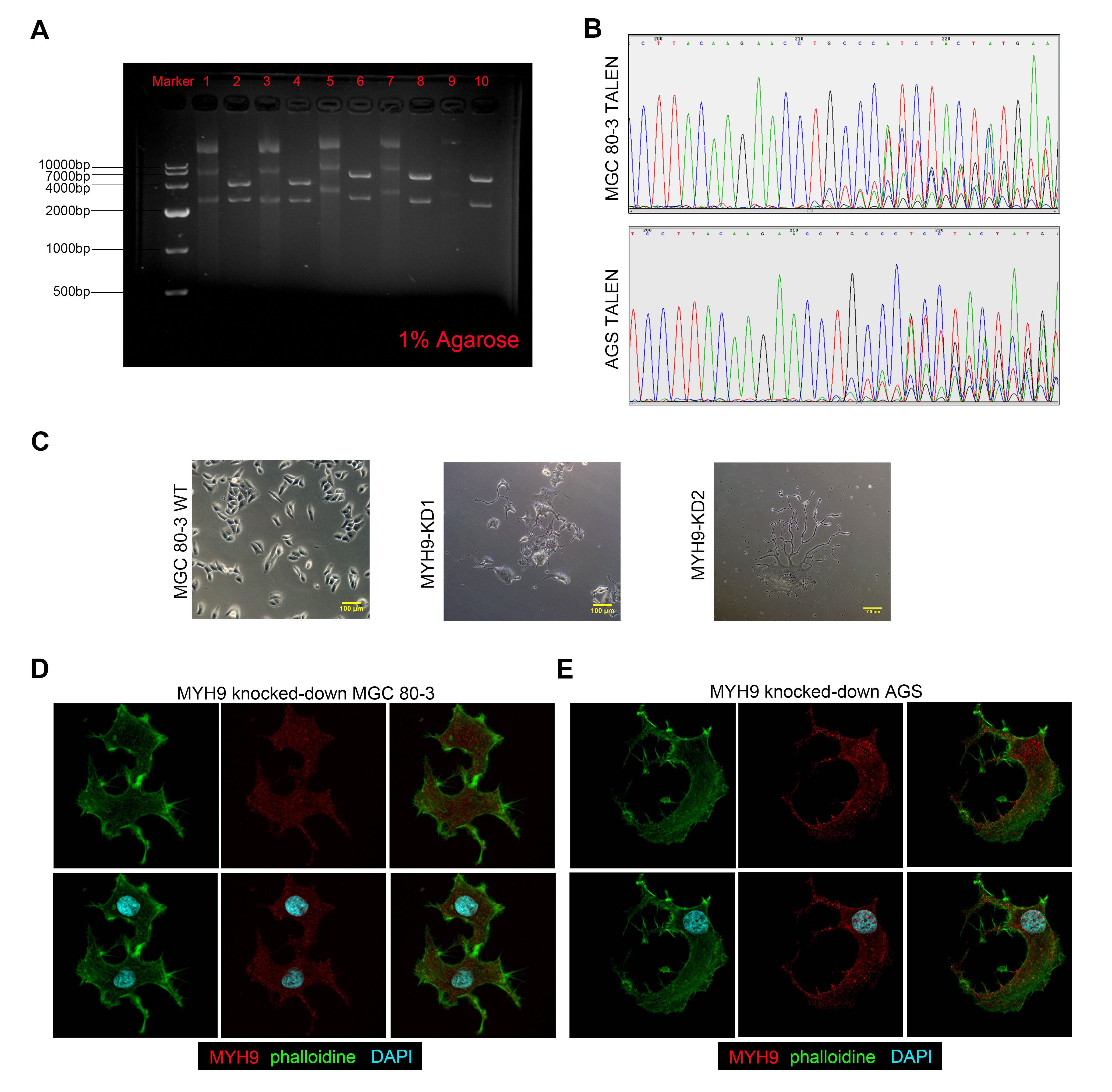


**Figure S7. Downregulation of MYH9 expression by transfecting GC cells with TALEN plasmids leads to cell morphological change as found in MYH9 shRNA3-infected cells**

(A) Gel electrophoresis. The TALEN plasmids constructed to target and knockdown MYH9 expression contained two left arm plasmids (L1 and L2) and three right arm plasmids (R1, R2 and R3). Five lanes (lane 1, 3, 5, 7 and 9) show undigested plasmids (L1, L2, R1, R2 and R3, respectively), while the other five lanes (lane 2, 4, 6, 8, and 10) show linearized plasmids (L1, L2, R1, R2 and R3, respectively). The combination of L2 and R3 plasmids were confirmed to be the most effective method for knocking down the MYH9 gene in GC cells (Table S6, S7). (B) Sequencing peak of MYH9 gene editing by TALEN. MGC 80-3 and AGS cells were transfected with the TALEN MYH9 knockdown plasmids and then subjected to genomic DNA extraction and sequencing. (C) Cell morphology. MGC 80-3 cells were grown and transfected with the TALEN MYH9 knockdown plasmids. Then, single cells were chosen for serial subcultivation and subjected to DNA sequencing. We successfully established 50 monoclonal sublines for MGC 80-3 and 26 for AGS (Figure 2C, Table S7). The DNA sequencing data revealed that at least one of the alleles did not undergo a frame shift mutation in these sublines (Table S7), leading to different degrees of MYH9 expression (data not shown). Three representative monoclonal cell lines are shown (C). WT, one cell line confirmed to be wild type MGC 80-3 by DNA sequencing; KD1, one cell line with MYH9 knockdown; KD2, one cell line that could not proliferate and eventually died. Most of these sublines showed looseness of the intercellular connection (such as MGC 80-3 MYH9-KD1), as shown in MYH9 shRNA3-infected cells (Figure 2A). Some TALEN plasmid-treated GC single cells had long pseudopods and failed to proliferate (such as MGC 80-3 MYH9-KD2), which might be MYH9 knockout (KO) cells [[14](#_ENREF_14)]. MGC 80-3 (D) and AGS (E) cells with MYH9 knockdown by TALEN technology were grown and then subjected to immunofluorescence staining of MYH9, phalloidin (a cell skeleton marker) and DAPI before analysis using immunofluorescence microscopy.


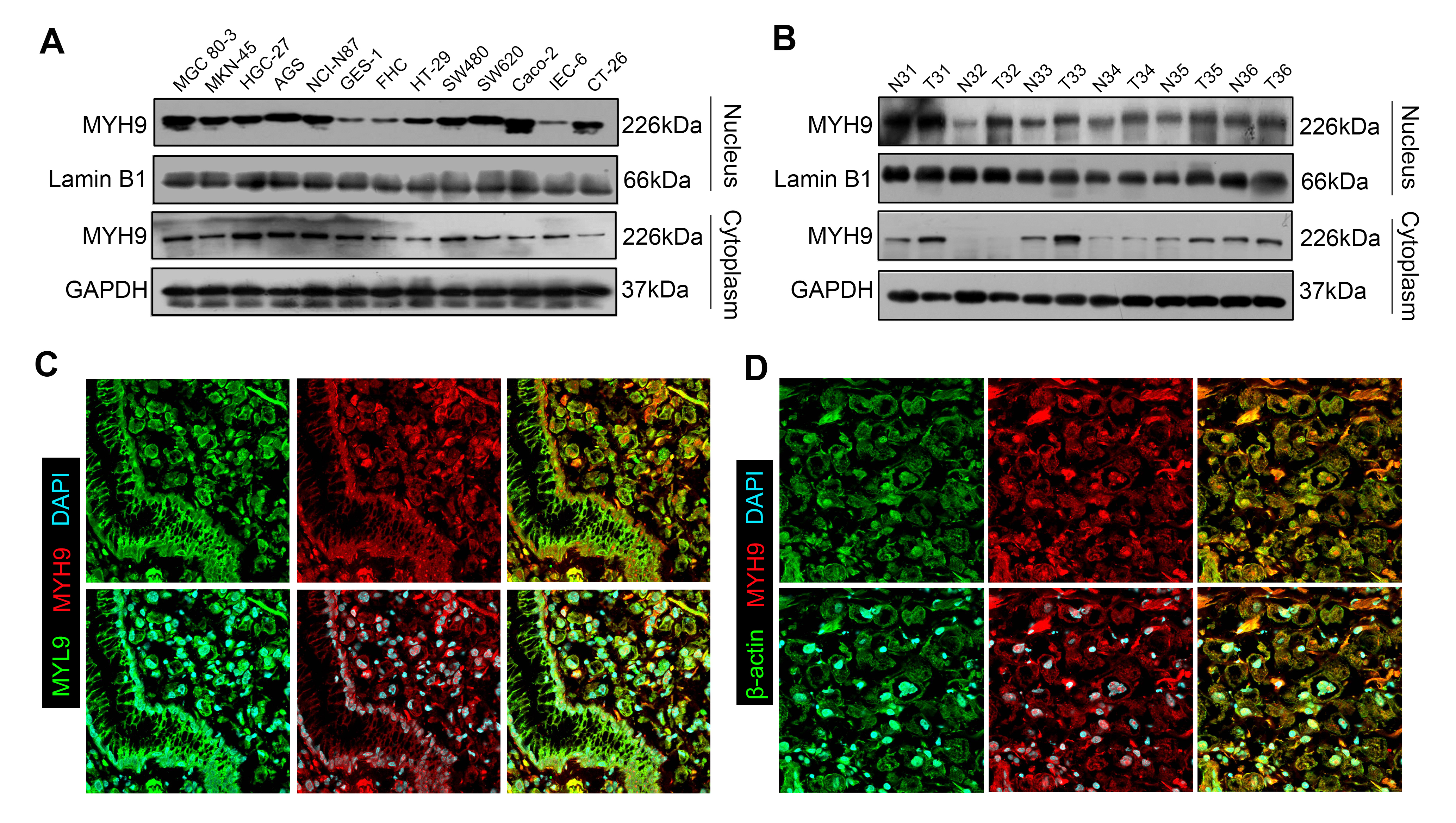


**Figure S8. MYH9 interacts with MYL9 and β-actin in the nucleus of GC cells and tissues**

(A) Human GC cell lines (MGC 80-3, MKN-45, HGC-27, AGS and NCI-N87), human gastric epithelial GES-1 cell line, human normal colonic epithelial FHC cell line, human colorectal cancer cell lines (SW480, SW620 and Caco2) and murine colorectal cancer CT-26 cell line were grown and subjected to western blot. (B) GC and paired normal tissues were used for western blot analysis. Lamin B1 and GAPDH proteins served as internal controls of nuclear and cytoplasmic proteins, respectively. GC tissues were processed and sectioned for immunofluorescence staining of MYH9, MYL9 (C) and β-actin (D) proteins.


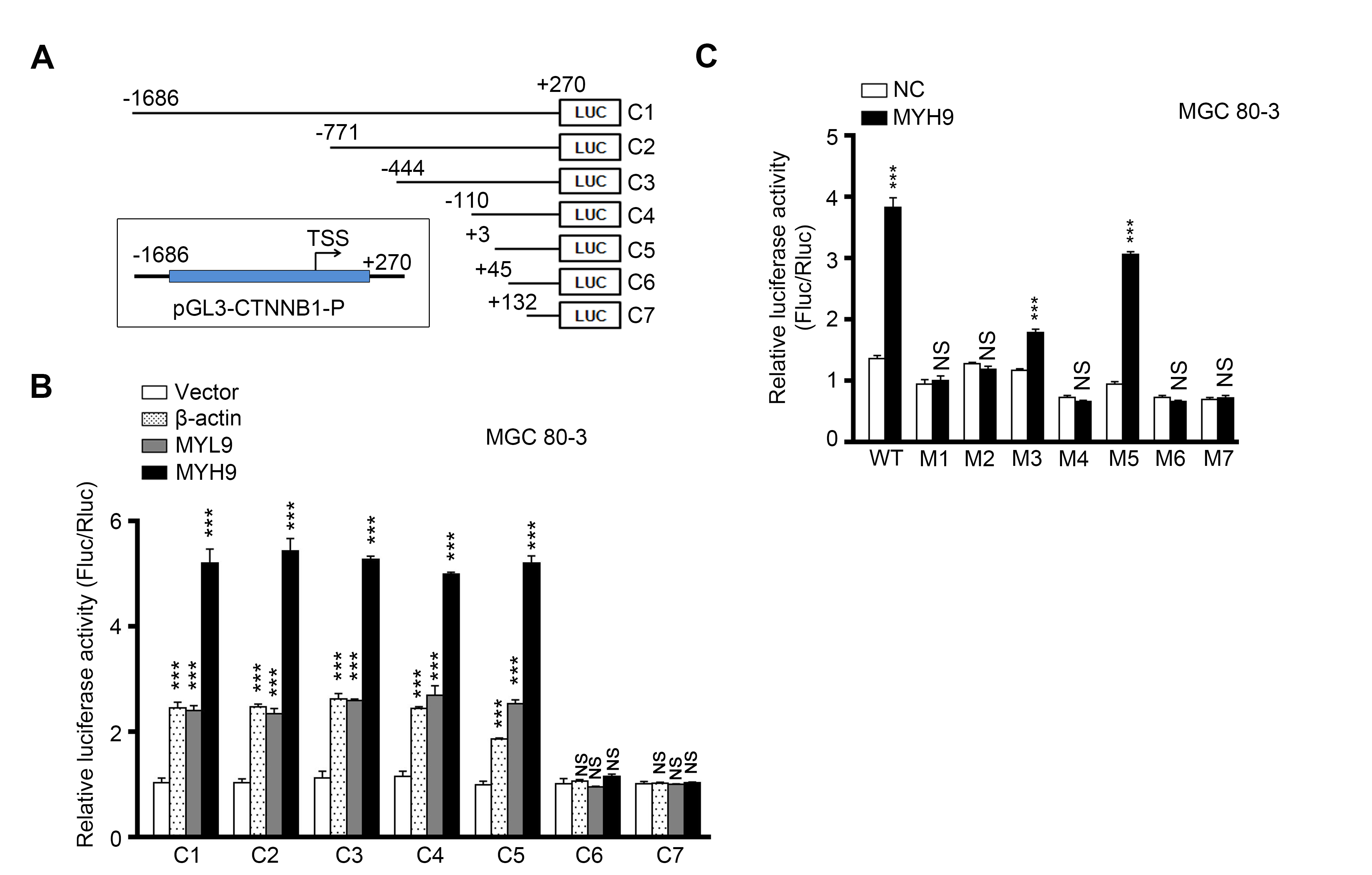


**Figure S9. MYH9 interacts with MYL9 and β-actin to bind to the *CTNNB1* promoter and promotes CTNNB1 transcription**

(A) Schematic representation of CTNNB1 promoter with different lengths that were cloned into the upstream region of the luciferase reporter vector. (B) MGC 80-3 cells were co-transfected with a Firefly luciferase reporter plasmid, a Renilla luciferase reporter plasmid and a gene overexpression plasmid for 24 h, and then subjected to luciferase reporter assay. The Firefly luciferase reporter plasmid contained different lengths of the *CTNNB1* promoter cDNA (C1–7). The gene overexpression plasmids contained MYH9, MYL9 or β-actin cDNA. (C) MYH9-overexpressed (LV-MYH9) MGC 80-3 cells were co-transfected with a Firefly luciferase reporter plasmid (M1-7; see also Figure 4E) and a Renilla luciferase reporter plasmid for 24 h, and then subjected to luciferase reporter assay. WT, the C3 plasmid, served as a positive control.


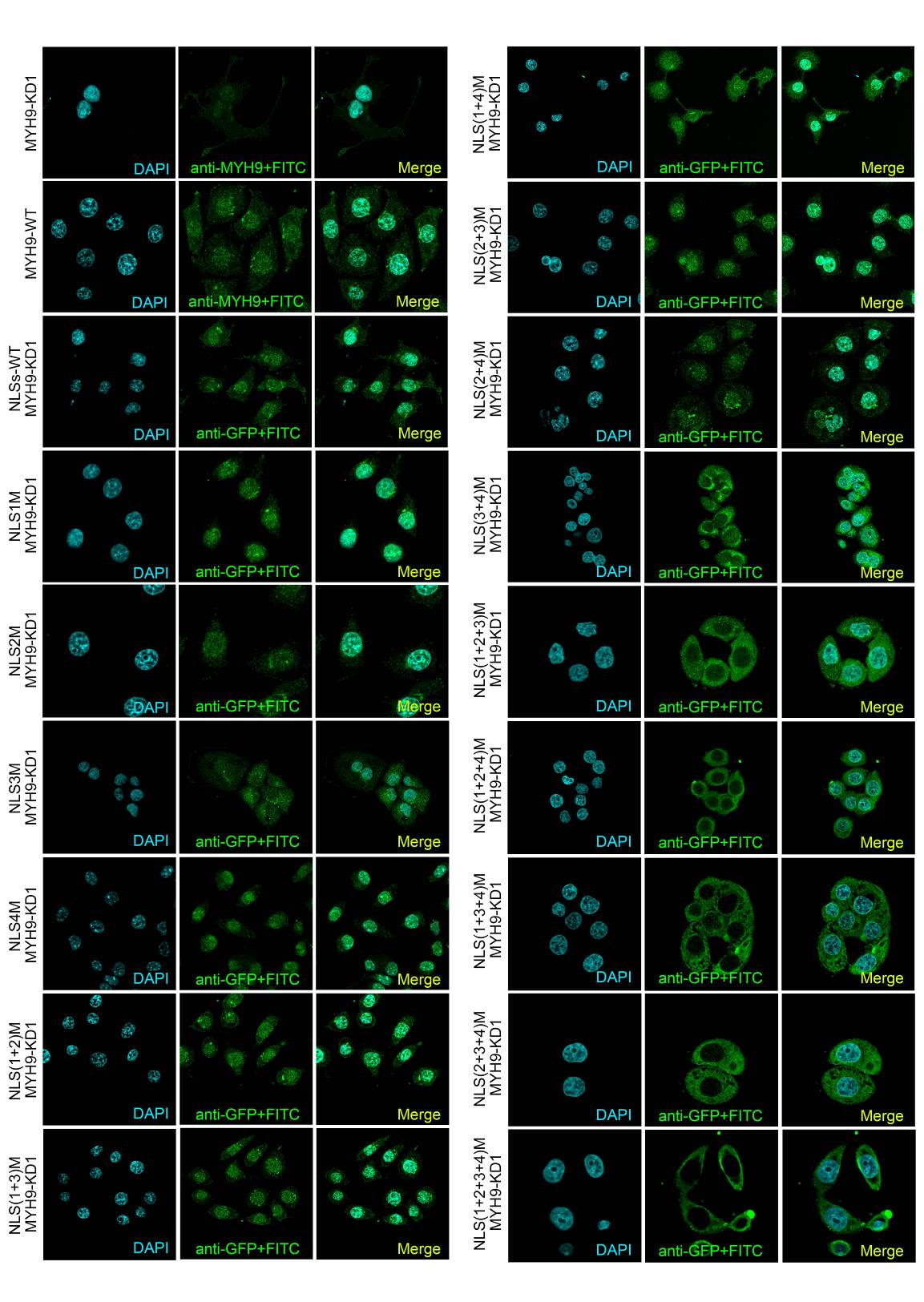


**Figure S10. The NLSs of MYH9 in GC cells**

TALEN-mediated MYH9 knockdown (MYH9-KD1) and wild type (MYH9-WT) MGC 80-3 cells were stained with anti-MYH9 and FITC secondary antibodies, while the MYH9-KD1 MGC 80-3 cells transfected with MYH9 plasmids containing different mutated NLSs were stained with anti-GFP and FITC secondary antibodies. Cellular localization of the GFP-tagged MYH9 (green) was imaged by confocal microscopy.


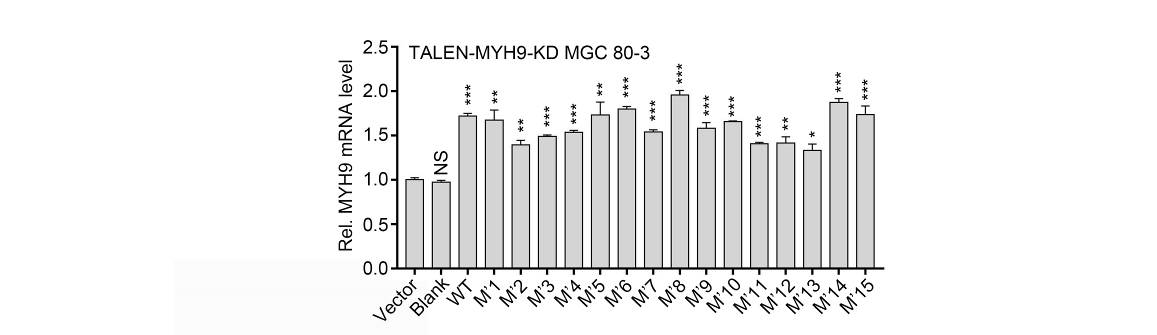


**Figure S11. Overexpression of MYH9 plasmids with various mutated NLSs in GC cells**

MGC 80-3 cells with MYH9 knockdown (TALEN-KD) were transiently transfected with different MYH9 NLS mutation plasmids (M'1-15) and then subjected to qPCR analysis of MYH9 mRNA.


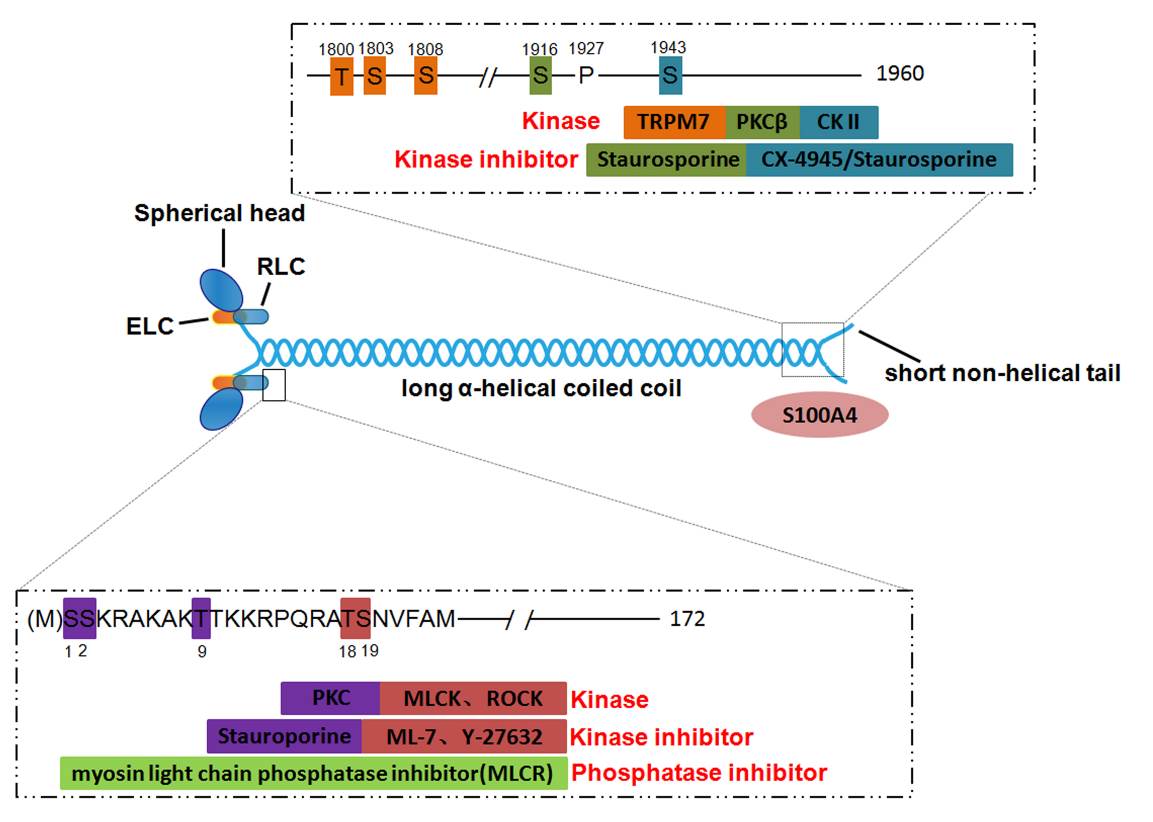


**Figure S12. Illustration of the key phosphorylation sites of MYH9 protein and the kinase or phosphatase inhibitor-binding sites.** Modified from previous studies [[15](#_ENREF_15), [16](#_ENREF_16)].


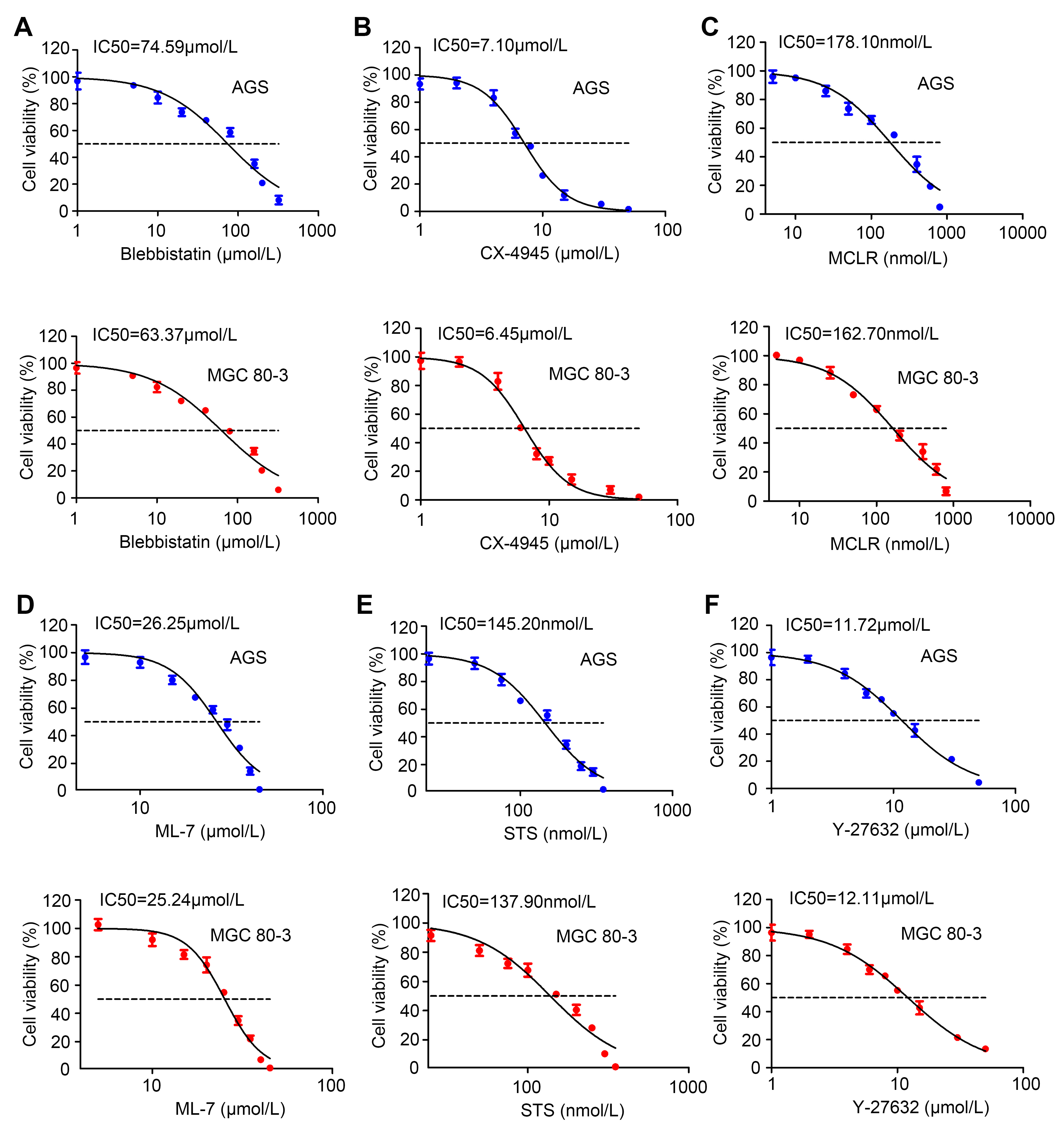


**Figure S13. The IC_50_ values of different drugs in GC cells**

The IC_50_ values of blebbistatin (A), CX-4945 (B), MCLR (C), ML-7 (D), STS (E) and Y-27632 (F) in AGS and MGC80-3 cells were evaluated by exposing cells to varying concentrations of the drugs (linear concentration gradients) for 24 h, and then subjecting cells to the cell viability CCK-8 assay.


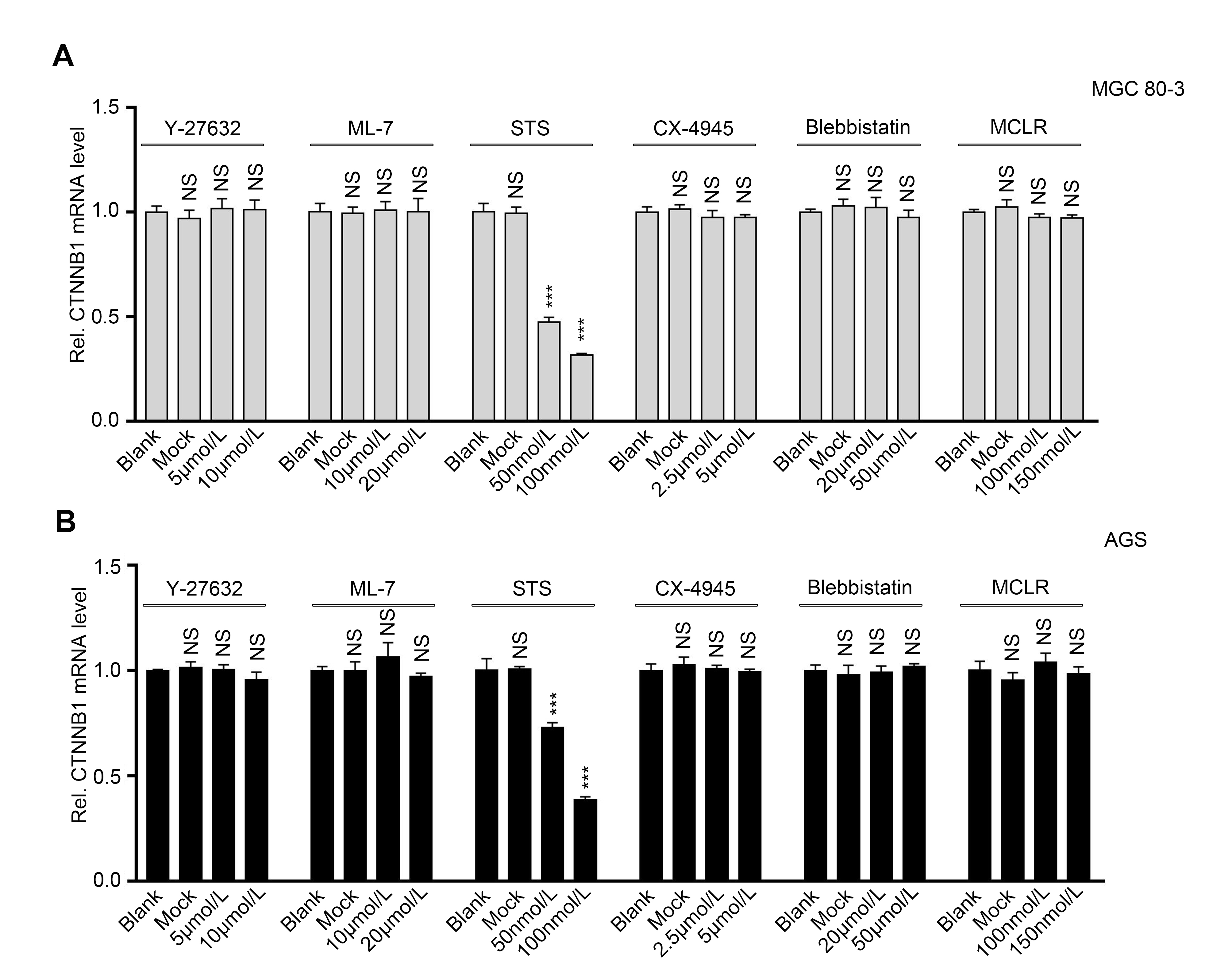


**Figure S14. CTNNB1 mRNA in MGC 80-3 and AGS cells treated with different drugs**

MGC 80-3 (A) and AGS (B) cells were grown and treated with six different drugs for 24 h and then subjected to qPCR analysis of CTNNB1 mRNA.


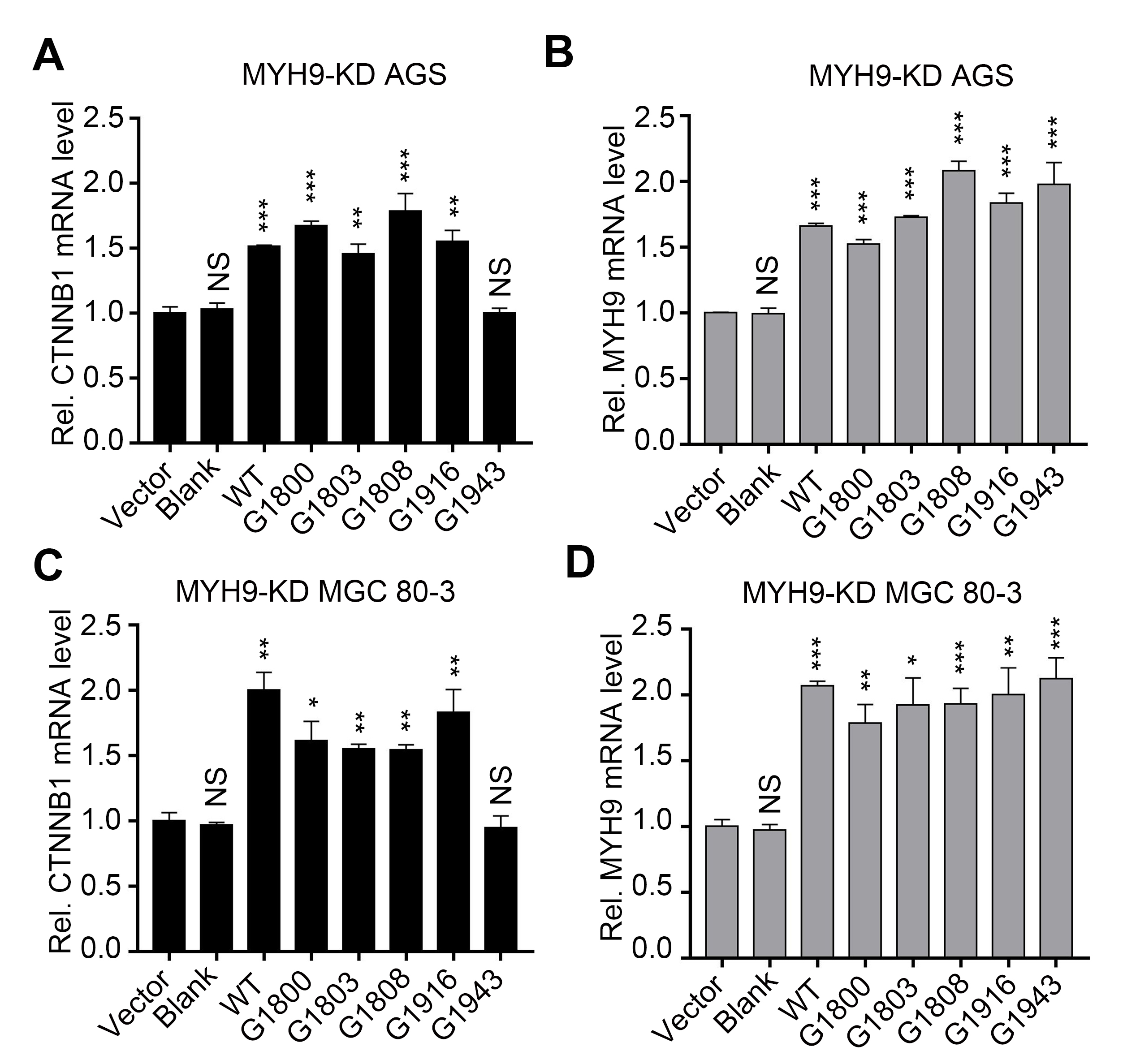


**Figure S15. MYH9 phosphorylation at S1943 promotes CTNNB1 transcription**

AGS (A, B) and MGC 80-3 (C, D) cells with MYH9 knockdown (MYH9-KD) were transfected with different MYH9 mutation plasmids (G1800, G1803, G1808, G1916 and G1943) and then subjected to qPCR analysis of CTNNB1 (A, C) and MYH9 (B,D) mRNAs.


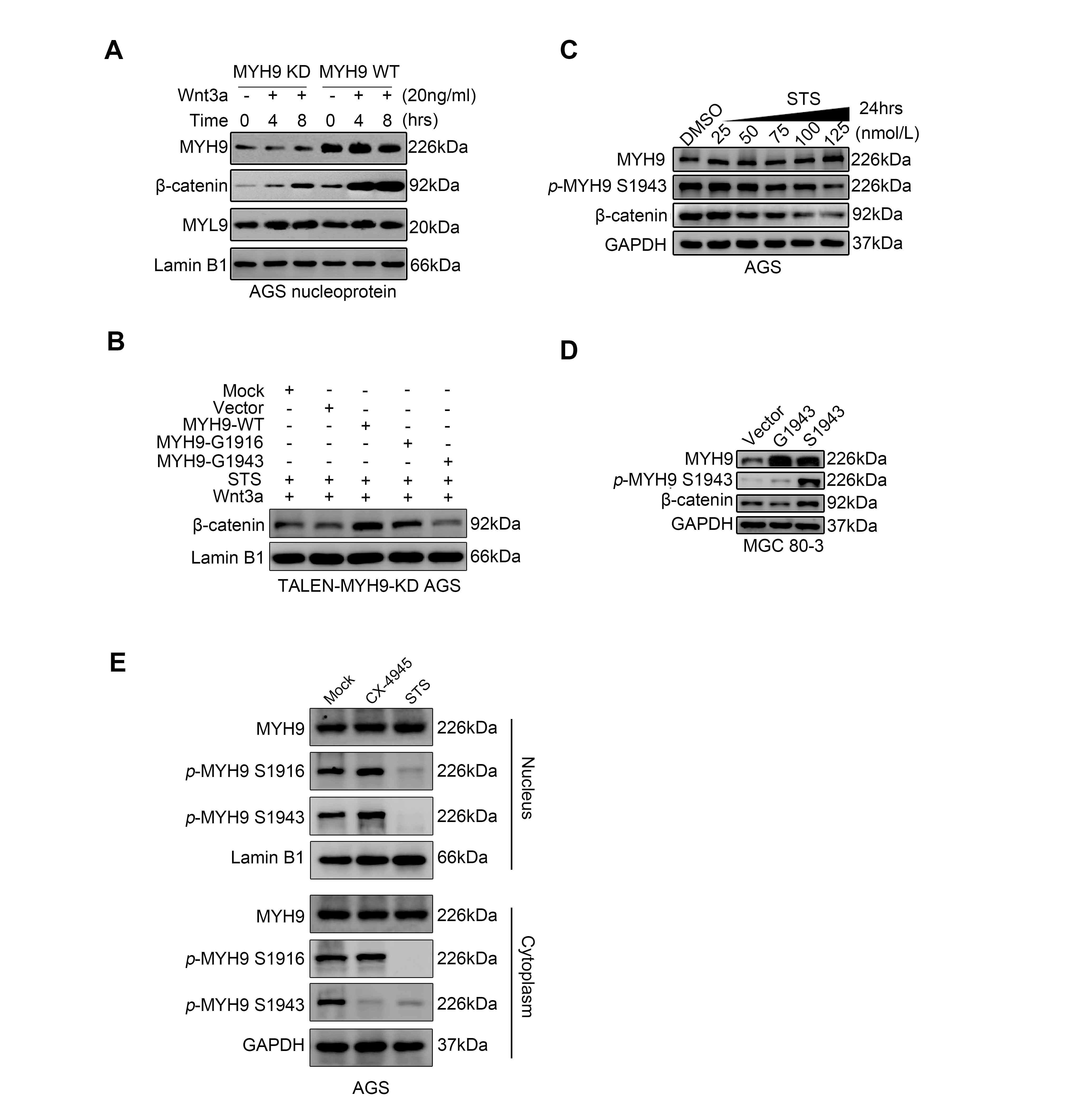


**Figure S16. MYH9 phosphorylation at S1943 promotes CTNNB1 transcription and wnt3a-activated β-catenin accumulation, which can be inhibited by STS**

(A) AGS cells were treated with Wnt3a (20 ng/mL) for 0, 4 or 8 h, and then were subjected to nuclear protein extraction and western blot analysis of MYH9, β-catenin and MYL9. MYH9 KD, AGS cells with MYH9 knockdown. MYH9 WT, wild-type AGS cells. Lamin B1 served as the internal control for nuclear protein. (B) AGS cells with MYH9 knockdown (TALEN-MYH9-KD) were transfected with MYH9 mutation plasmids (G1916 or G1943), treated with STS (100 nmol/L) for 24 h and Wnt3a (20 ng/mL) for 8 h, and then subjected to nuclear protein extraction and western blot analysis of β-catenin expression. (C) AGS cells were treated with STS (25, 50, 75, 100 and 125 nmol/L) for 24 h and then subjected to western blot analysis of *p*-MYH9 (S1943) and β-catenin levels. (D) MGC 80-3 cells were transfected with MYH9 G1943 or S1943 plasmids, and then subjected to western blot analysis of *p*-MYH9 (S1943) and β-catenin levels. (E) AGS cells were grown and treated with STS (100 nmol/L) or CX-4945 (5 μmol/L) for 24 h, and then subjected to nuclear and cytoplasmic protein extraction and western blot.


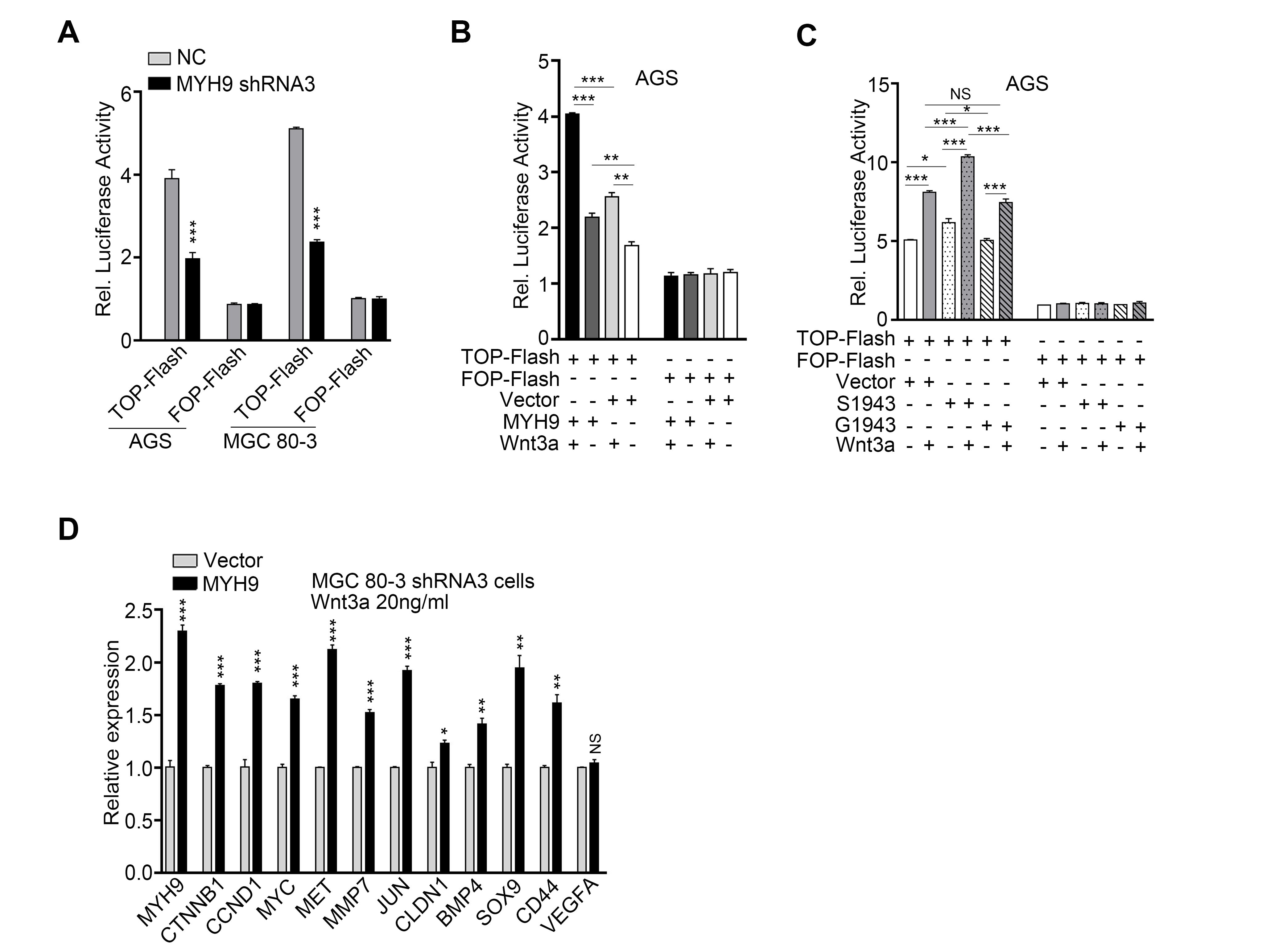


**Figure S17. MYH9-mediated CTNNB1 transcription increases the activation of wnt/β-catenin signaling in GC cells**

(A) AGS and MGC 80-3 cells with MYH9 shRNA3 were co-transfected with a luciferase reporter plasmid (TOP-flash or FOP-flash) and the Renilla control plasmid for 16 h, and then treated with Wnt3a (20 ng/ml) for 8 h and subjected to the luciferase reporter assay. NC, a negative control of MYH9 shRNA3. (B) AGS cells were co-transfected with the MYH9 plasmid, luciferase reporter plasmid (TOP-flash or FOP-flash) and the Renilla control plasmid for 16 h, and then treated with Wnt3a (20 ng/mL) for 8 h and subjected to the luciferase reporter assay. (C) AGS cells were co-transfected with MYH9 plasmid (1943S, 1943G or its control vector), luciferase reporter plasmid (TOP-flash or FOP-flash) and the Renilla control plasmid for 16 h, and then treated with Wnt3a (20 ng/mL) for 8 h and subjected to the luciferase reporter assay. (D) The qPCR analysis was performed to measure the mRNA levels of β-catenin-induced target genes in MGC 80-3 MYH9 shRNA3 cells transfected with the MYH9 plasmid for 24 h. The cells were treated with Wnt3a (20 ng/mL) for 8 h before being harvested for RNA cleavage. Vector was used as a negative control.


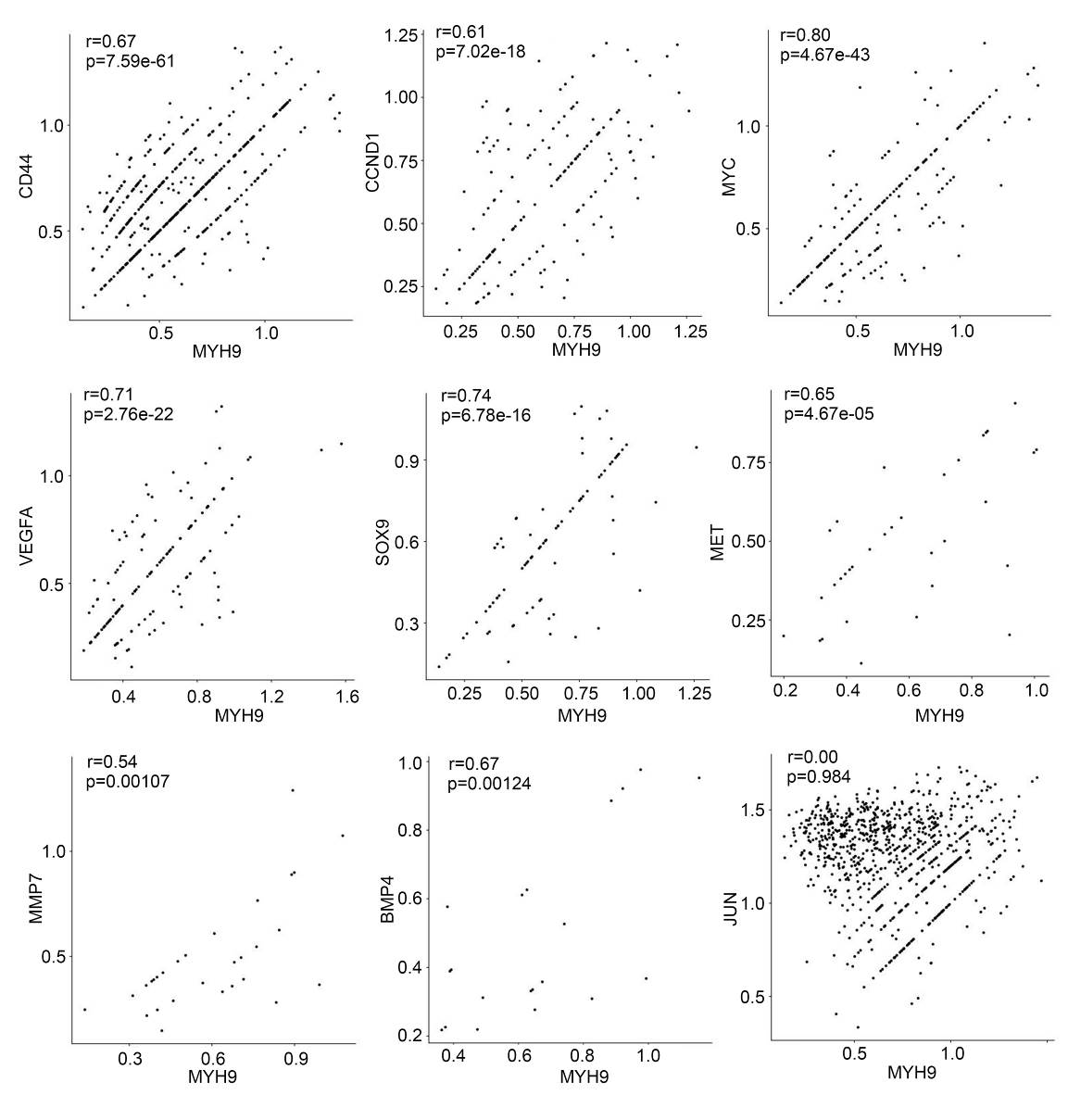


**Figure S18. MYH9 mRNA expression was positively associated with expression of β-catenin-induced mRNAs in GC tissues**

The association of MYH9 mRNA with levels of β-catenin-induced mRNAs (e.g., CD44, CCND1 and MYC) was analyzed in gastric epithelial-derived cells (gastric epithelial cells and GC cells) using single-cell transcriptome data.


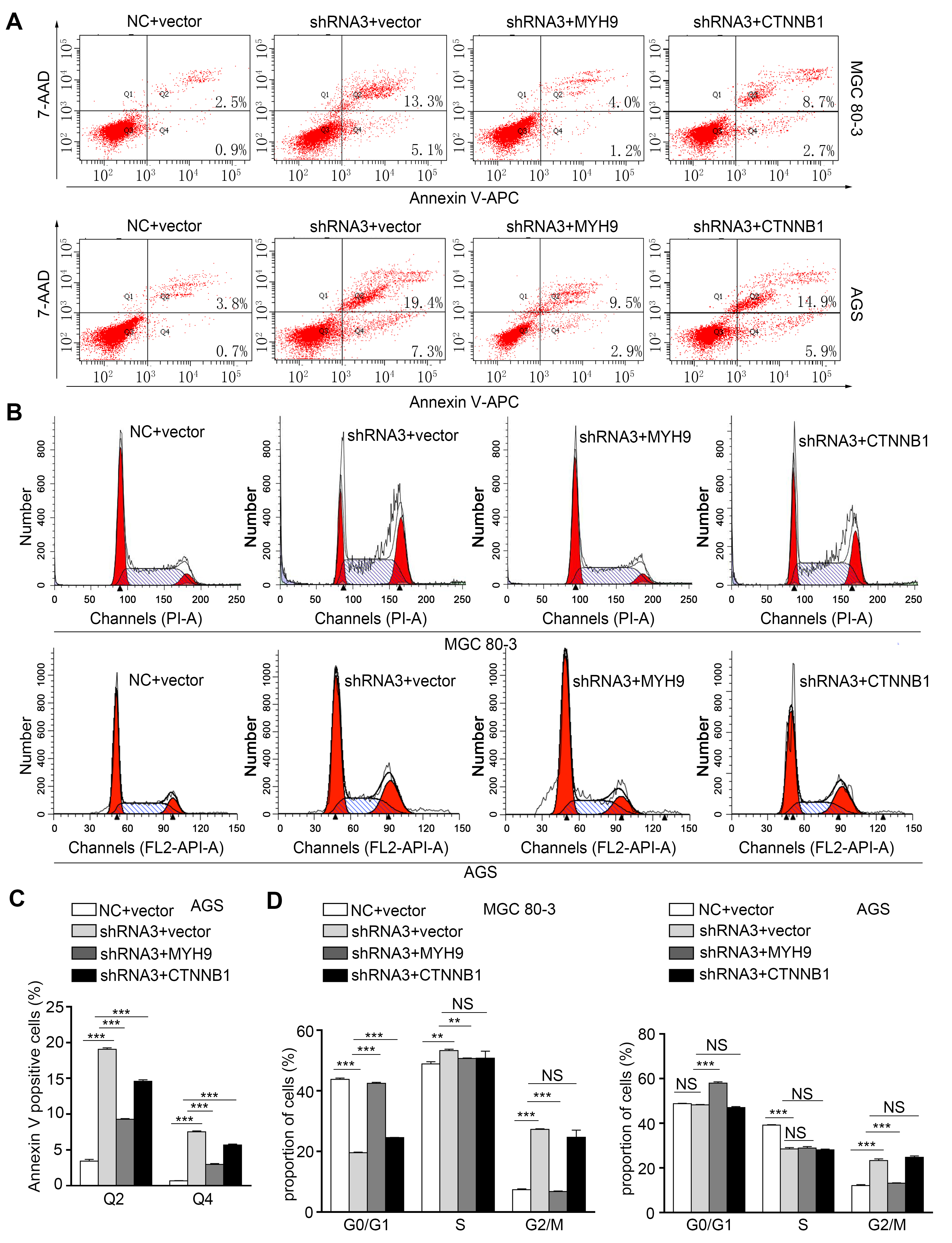


**Figure S19. MYH9-mediated CTNNB1 transcription inhibits GC apoptosis but not the cell cycle transition from G2 to M**

(A) Flow cytometric apoptosis assay. AGS and MGC 80-3 cells transfected with NC+vector, shRNA3+vector, shRNA3+MYH9 or shRNA3+CTNNB1 were subjected to flow cytometric annexin V and 7-AAD assays. The cells stained with annexin V were considered apoptotic and the data were presented as percent of apoptotic cells. (B) Flow cytometry cell cycle distribution assay. The duplicated cells were analyzed with the cell cycle distribution assay. (C) Summarized data of apoptosis assay (see also in Figure 6C and S19A). (D) Summarized data of cell cycle assay in AGS and MGC 80-3 cells (see also in S19B).


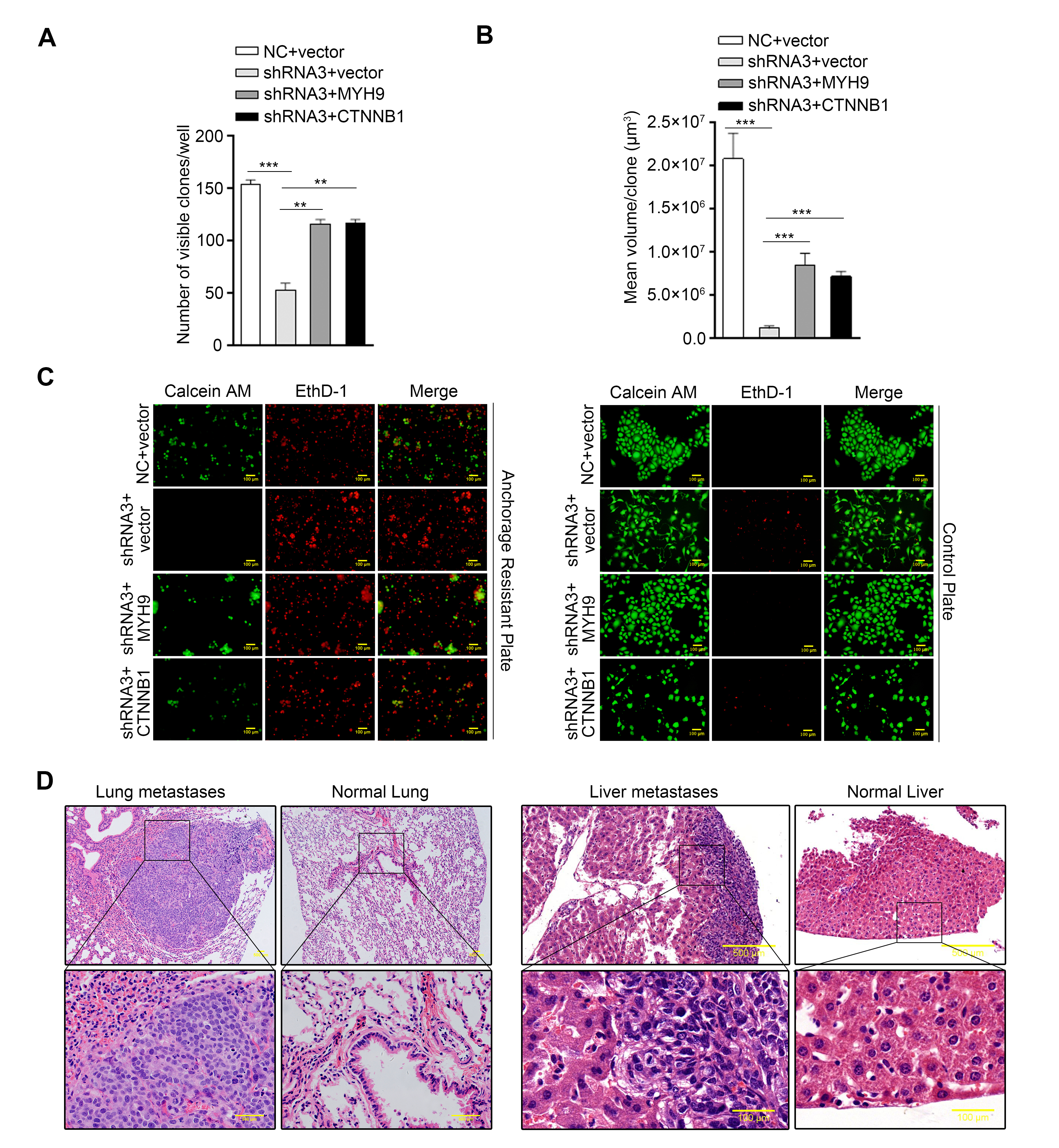


**Figure S20. MYH9-mediated CTNNB1 transcription promotes anoikis resistance *in vitro* and *in vivo***

(A-B) MGC 80-3 cells transfected with different plasmids (NC+vector, shRNA3+vector, shRNA3+MYH9 or shRNA3+CTNNB1) were seeded into a 12-well plate for 21 days, and then subjected to a soft agar colony formation assay. (A) Histogram analysis of the number of visible clones per well. (B) Histogram analysis of the mean volume per clone. (C) MGC 80-3 cells transfected with different plasmids (NC+vector, shRNA3+vector, shRNA3+MYH9 or shRNA3+CTNNB1) were seeded into a poly-hema pre-coated plate (or a control cell culture plate) to grow for 24 h and then subjected to the calcein AM/EthD-1 fluorometric detection assay. The live cells were stained with calcein AM (green), while the anoikis-like cells were stained with EthD-1 (red). (D) MGC 80-3 cells transfected with different plasmids (NC+vector, shRNA3+vector, shRNA3+MYH9 or shRNA3+CTNNB1) were injected into the mouse tail vein. After eight weeks, the mice were sacrificed, and different organs were resected. The lung and liver metastases were detected by hematoxylin & eosin (H&E) staining.


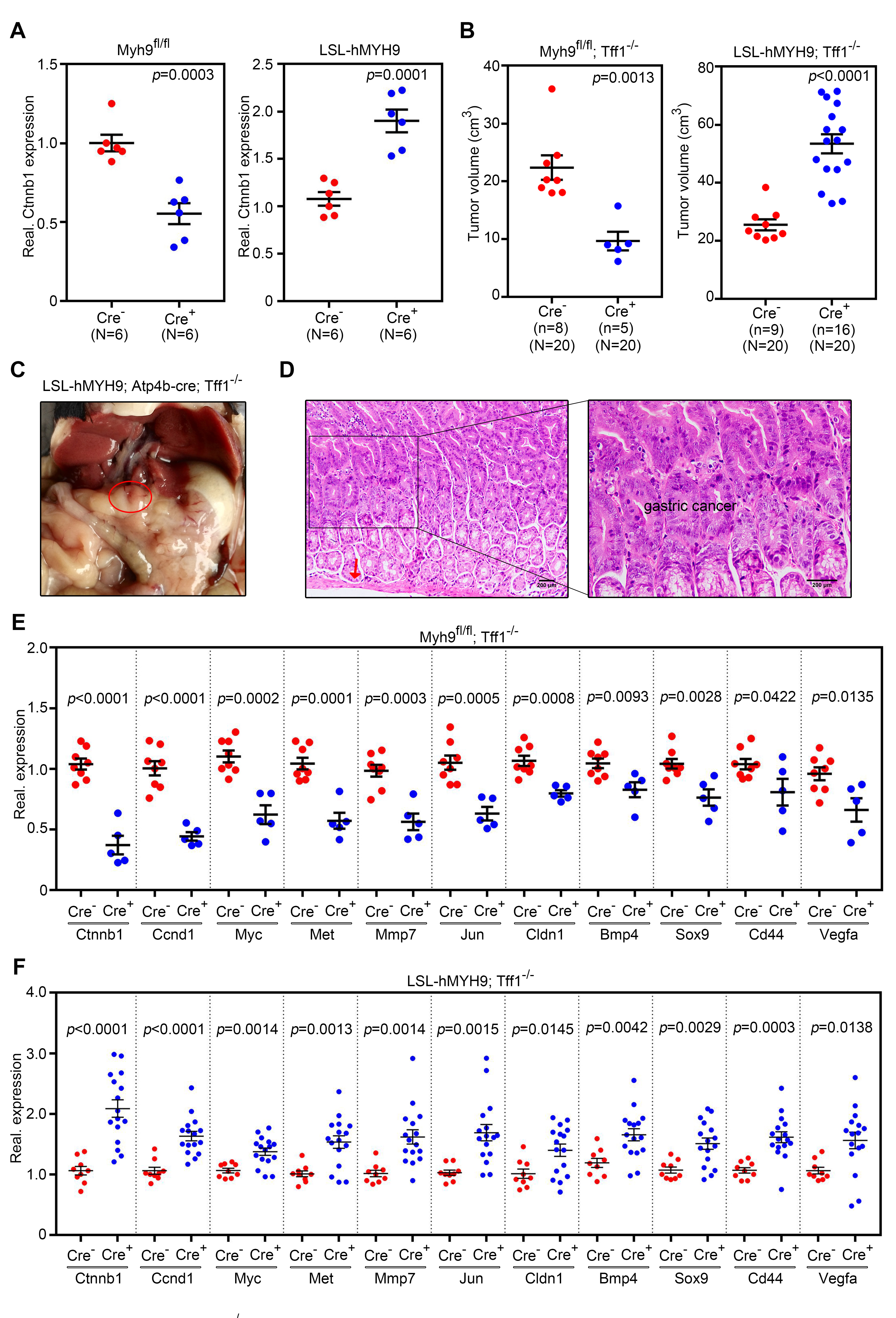


**Figure S21. MYH9-mediated CTNNB1 expression promotes gastric cancer progression and cancer cell metastasis *in vivo***

(A) Level of Ctnnb1 mRNA was assessed using qPCR in gastric tissue samples from conditional Myh9 knockout mice (Myh9^fl/fl^; Atp4b-cre; Tff^-/-^) or hMYH9 transgenic mice (LSL-hMYH9; Atp4b-cre; Tff^-/-^ mice). (B) The stomach tumor volume in Myh9^fl/fl^; Atp4b-cre; Tff^-/-^ mice compared with that in Myh9^fl/fl^; Tff^-/-^ mice, or the stomach tumor volume in LSL-hMYH9; Atp4b-cre; Tff^-/-^ mice compared with that in LSL-hMYH9; Tff^-/-^ mice. N, the total number of mice in the experiment; n, the number of mice with GC. (C) A representative image of invasive GC in LSL-hMYH9; Atp4b-cre; Tff^-/-^ mice. The red circle indicates a tumor lesion. (D) GC tissues in LSL-hMYH9; Atp4b-cre; Tff^-/-^ mice were detected by hematoxylin & eosin (H&E) staining. The red arrow indicates infiltrating GC cells. (E) qPCR. GC tissues from Myh9^fl/fl^; Atp4b-cre^-^ or Myh9^fl/fl^; Atp4b-cre^+^ mice were resected and then subjected to qPCR analysis of β-catenin-induced target genes. (F) GC tissues from LSL-hMYH9; Atp4b-cre^-^ or LSL-hMYH9; Atp4b-cre^+^ mice were resected and subjected to qPCR analysis of β-catenin-induced target genes.


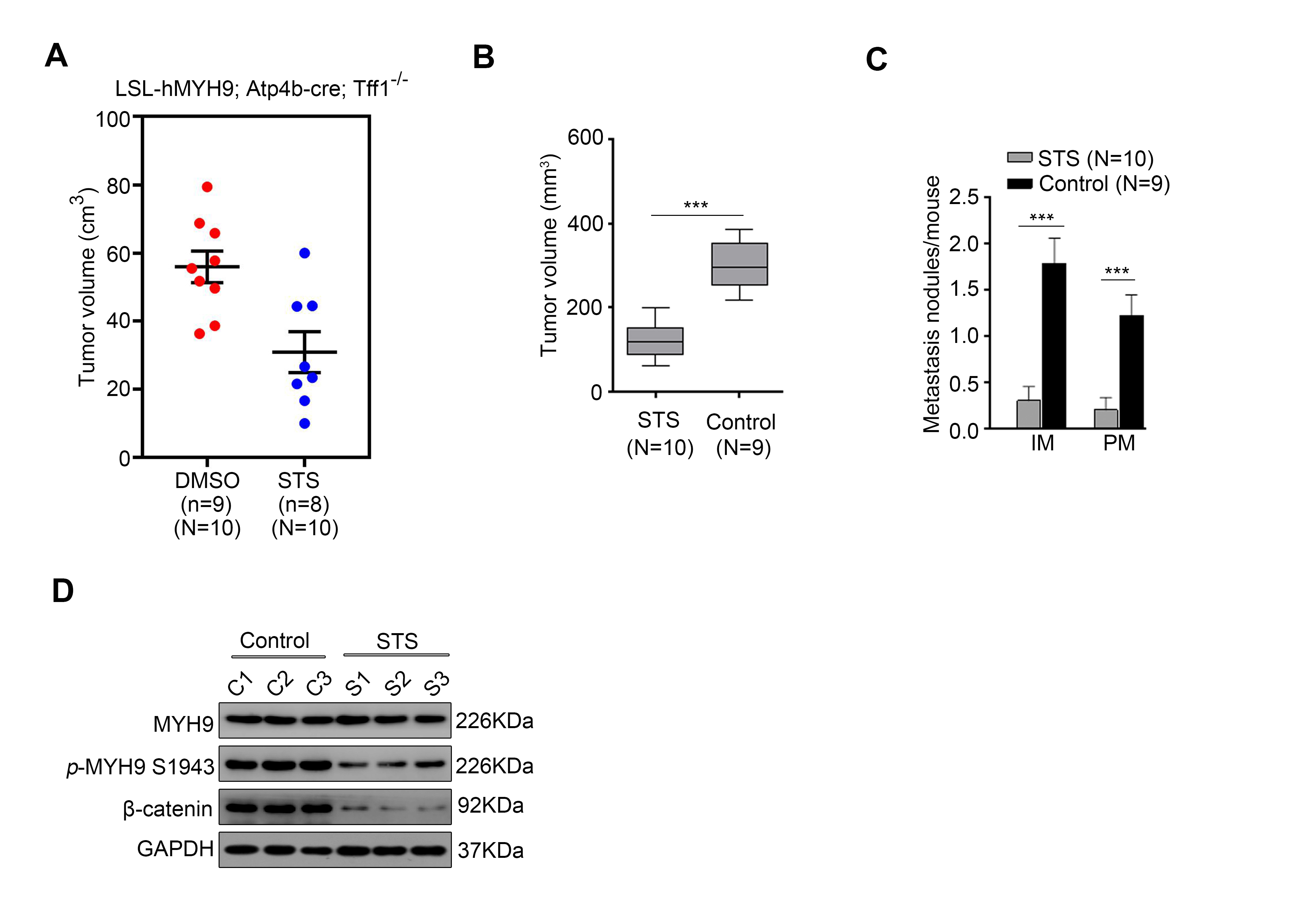


**Figure S22. STS inhibits stomach tumor progression *in vivo* by decreasing MYH9 phosphorylation at S1943**

(A) The stomach tumor volumes from LSL-hMYH9; Atp4b-cre; Tff1^-/-^ mice treated with STS (9/10) or DMSO (8/10) were analyzed. N, the total number of mice in the experiment; n, the number of mice with GC. (B) The primary tumor volumes of GC orthotropic xenograft nude mouse models were analyzed. (C) The metastatic nodules per GC orthotropic xenograft nude mouse were counted. (D) Three primary GC tissues from orthotropic xenograft nude mice treated with STS (or DMSO) were used for western blot analysis of *p*-MYH9 (S1943) and β-catenin levels.


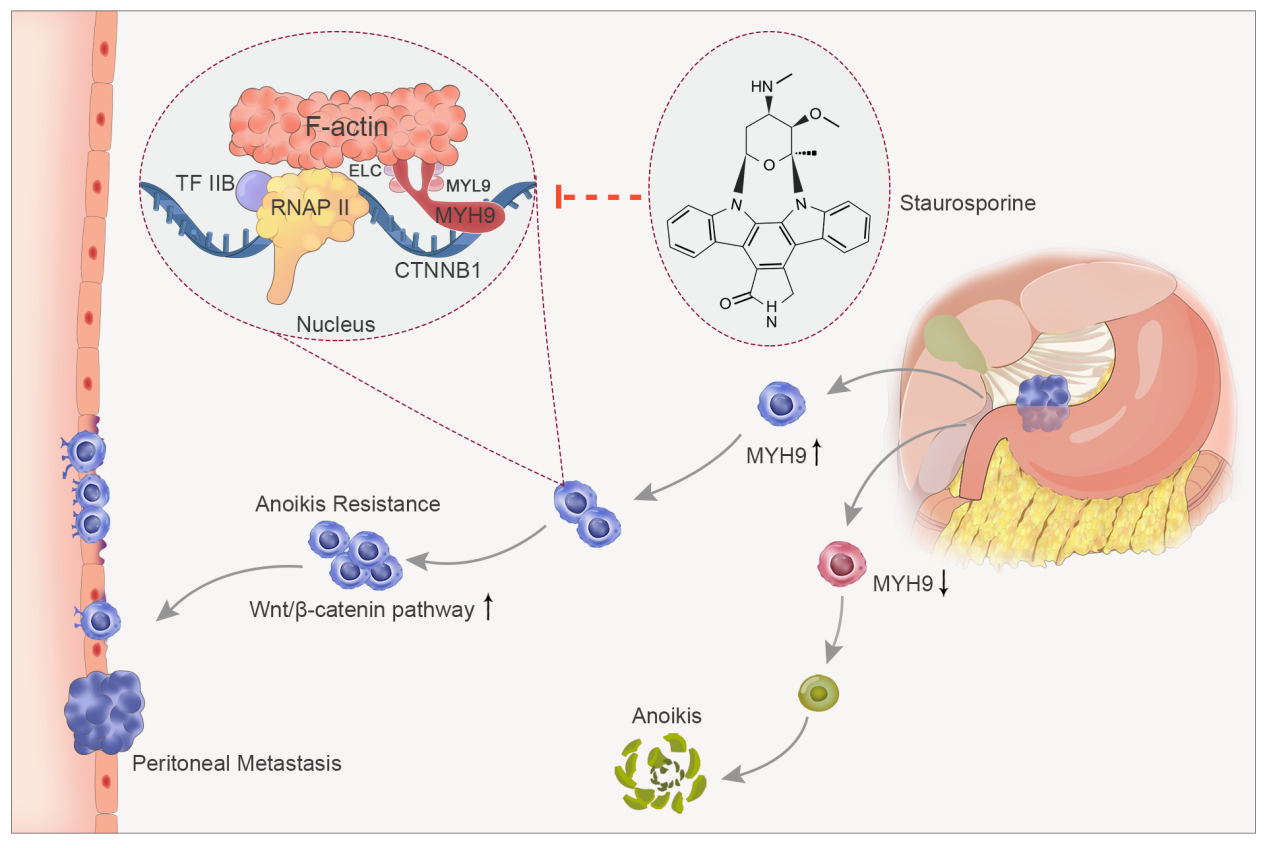


**Figure S23. Graphical Abstract**

Nuclear MYH9 binds to the CTNNB1 promoter at its DNA binding domain, and interacts with myosin light chain 9, β-actin and RNA polymerase II to promote CTNNB1 transcription, which conferred a resistance to anoikis in GC cells. GC cells with low MYH9 expression easily undergo anoikis, while those cells with high MYH9 expression continue to proliferate and form peritoneal metastases. Staurosporine (STS) is able to inhibit nuclear MYH9 S1943 phosphorylation, CTNNB1 transcription and canonical Wnt/β-catenin signaling. These data reveal a potential method through which GC peritoneal metastasis can be inhibited.


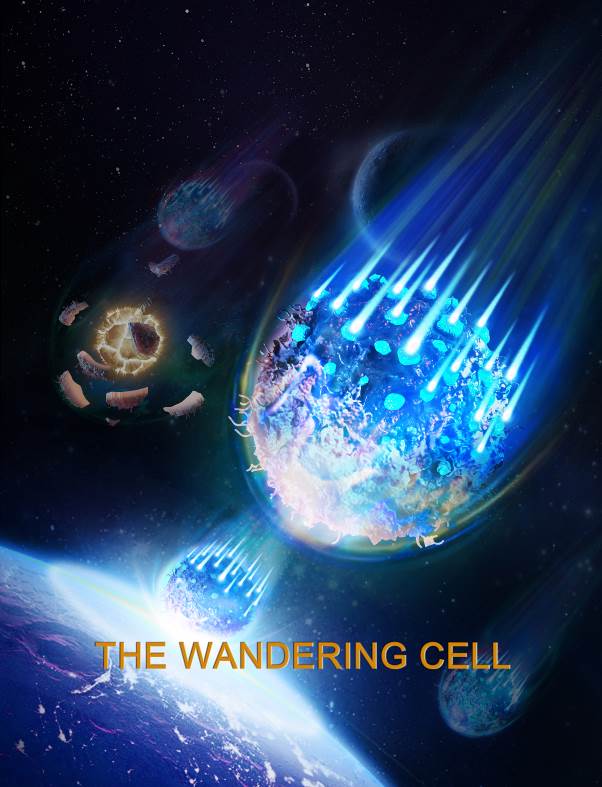


**Figure S24. Research story**

Our research focuses on the mechanism of gastric cancer peritoneal metastasis. In this study, we found nuclear MYH9 promoted CTNNB1 transcription, which lead to enhanced tumor cell anoikis resistance, and staurosporine was able to inhibit this effect. As a metaphor for the recent science fiction movie "Wandering Earth", the first such film in China, MYH9 is the "excavator", β-catenin acts as the "rocks", while Wnt is the "heavy fusion propulsion system". Though stromal cells in the tumor microenvironment are important for cancer metastasis, the "self-improvement" of tumor cells is critical for gastric cancer peritoneal metastasis by wandering in the peritoneal cavity. Cancer cells with high MYH9 expression were prone to avoid anoikis for survival before forming a metastatic lesion.

**Supplementary Tables**

**Table S1. CyDye labeling of GC tissue samples**

| Gel # | Cy2 (50 μg) | Cy3 (50 μg) | Cy5 (50 μg) |
| --- | --- | --- | --- |
| Gel 1 | Pooled | Gastric epithelia | Primary GC |
| Gel 2 | Pooled | Primary GC | Peritoneal metastases |
| Gel 3 | Pooled | Peritoneal metastases | Gastric epithelia |
| Gel 4 | Pooled | Primary GC | Gastric epithelia |
| Gel 5 | Pooled | Peritoneal metastases | Primary GC |
| Gel 6 | Pooled | Gastric epithelia | Peritoneal metastases |

**Table S2. DEPs expressed in GC versus normal tissues identified by MALDI-TOF/TOF**

**Table S3. Primer sequences used for qPCR in this study**

| Gene | Primer sequence | | GC (%) | Tm (°C) |
| --- | --- | --- | --- | --- |
| MYH9 |  | 5'-ACCATGGAGGCCATGAGGATTA-3' | 50.0 | 60.97 |
|  |  | 5'-CGATGTTGCCGAGCTGAAGA-3' | 55.0 | 60.74 |
| CTNNB1 |  | 5'-GCTTGTTCGTGCACATCAGGATA-3' | 47.8 | 64.50 |
|  |  | 5'-GGCTCCGGTACAACCTTCAACTA-3' | 52.2 | 63.60 |
| CCND1 |  | 5'-GCTGCGAAGTGGAAACCATC-3' | 55.00 | 59.83 |
|  |  | 5'-CCTCCTTCTGCACACATTTGAA-3' | 45.45 | 59.11 |
| MYC |  | 5'-GGCTCCTGGCAAAAGGTCA-3' | 57.89 | 60.23 |
|  |  | 5'-CTGCGTAGTTGTGCTGATGT-3' | 50.00 | 58.57 |
| MET |  | 5'-AGCGTCAACAGAGGGACCT-3' | 57.89 | 60.53 |
|  |  | 5'-GCAGTGAACCTCCGACTGTATG-3' | 54.55 | 60.99 |
| MMP7 |  | 5'-GAGTGAGCTACAGTGGGAACA-3' | 52.38 | 59.38 |
|  |  | 5'-CTATGACGCGGGAGTTTAACAT-3' | 45.45 | 58.55 |
| JUN |  | 5'-TCCAAGTGCCGAAAAAGGAAG-3' | 47.62 | 59.05 |
|  |  | 5'-CGAGTTCTGAGCTTTCAAGGT-3' | 47.62 | 58.24 |
| VEGFA |  | 5'-AGGGCAGAATCATCACGAAGT-3' | 47.62 | 59.44 |
|  |  | 5'-AGGGTCTCGATTGGATGGCA-3' | 55.00 | 60.98 |
| CLDN1 |  | 5'-TCTGGCTATTTTAGTTGCCACAG-3' | 43.48 | 58.99 |
|  |  | 5'-AGAGAGCCTGACCAAATTCGT-3' | 47.62 | 59.37 |
| BMP4 |  | 5'-AAAGTCGCCGAGATTCAGGG-3' | 55.00 | 60.11 |
|  |  | 5'-GACGGCACTCTTGCTAGGC-3' | 63.16 | 60.81 |
| SOX9 |  | 5'-AGCGAACGCACATCAAGAC-3' | 52.63 | 58.85 |
|  |  | 5'-CTGTAGGCGATCTGTTGGGG-3' | 60.00 | 60.18 |
| CD44 |  | 5'-CTGCCGCTTTGCAGGTGT-3' | 61.11 | 61.29 |
|  |  | 5'-CATTGTGGGCAAGGTGCTATT-3' | 47.62 | 59.17 |
| Myh9 |  | 5'-GGCCCTGCTAGATGAGGAGT-3' | 60.00 | 60.76 |
|  |  | 5'-CTTGGGCTTCTGGAACTTGG-3' | 55.00 | 58.75 |
| Ctnnb1 |  | 5'-ATGGAGCCGGACAGAAAAGC-3' | 55.00 | 60.68 |
|  |  | 5'-CTTGCCACTCAGGGAAGGA-3' | 57.89 | 58.93 |
| Ccnd1 |  | 5'-GCGTACCCTGACACCAATCTC-3' | 57.14 | 60.74 |
|  |  | 5'-CTCCTCTTCGCACTTCTGCTC-3' | 57.14 | 60.74 |
| Myc |  | 5'-ATGCCCCTCAACGTGAACTTC-3' | 52.38 | 60.88 |
|  |  | 5'-CGCAACATAGGATGGAGAGCA-3' | 52.38 | 60.20 |
| Met |  | 5'-GTGAACATGAAGTATCAGCTCCC-3' | 47.83 | 59.13 |
|  |  | 5'-TGTAGTTTGTGGCTCCGAGAT-3' | 47.62 | 59.10 |
| Mmp7 |  | 5'-CTGCCACTGTCCCAGGAAG-3' | 63.16 | 60.00 |
|  |  | 5'-GGGAGAGTTTTCCAGTCATGG-3' | 52.38 | 58.28 |
| Jun |  | 5'-CCTTCTACGACGATGCCCTC-3' | 60.00 | 59.97 |
|  |  | 5'-GGTTCAAGGTCATGCTCTGTTT-3' | 45.45 | 59.11 |
| Cldn1 |  | 5'-GGGGACAACATCGTGACCG-3' | 63.16 | 60.74 |
|  |  | 5'-AGGAGTCGAAGACTTTGCACT-3' | 47.62 | 59.31 |
| Bmp4 |  | 5'-TTCCTGGTAACCGAATGCTGA-3' | 47.62 | 59.37 |
|  |  | 5'-CCTGAATCTCGGCGACTTTTT-3' | 47.62 | 58.92 |
| Sox9 |  | 5'-GAGCCGGATCTGAAGAGGGA-3' | 60.00 | 60.76 |
|  |  | 5'-GCTTGACGTGTGGCTTGTTC-3' | 55.00 | 60.32 |
| Cd44 |  | 5'-TCTGCCATCTAGCACTAAGAGC-3' | 50.00 | 59.64 |
|  |  | 5'-GTCTGGGTATTGAAAGGTGTAGC-3' | 47.83 | 59.06 |
| Vegfa |  | 5'-CTGCCGTCCGATTGAGACC-3' | 63.16 | 60.52 |
|  |  | 5'-CCCCTCCTTGTACCACTGTC-3' | 60.00 | 59.39 |
| Gapdh |  | 5'-AGGTCGGTGTGAACGGATTTG-3' | 52.38 | 60.88 |
|  |  | 5'-TGTAGACCATGTAGTTGAGGTCA-3' | 43.48 | 58.59 |
| GAPDH |  | 5'-GCACCGTCAAGGCTGAGAAC-3' | 60.0 | 61.57 |
|  |  | 5'-TGGTGAAGACGCCAGTGGA-3' | 57.89 | 61.14 |
| CTNNB1 |  | 5'-GCTTGTTCGTGCACATCAGGATA-3' | 47.83 | 61.47 |
|  |  | 5'-GGCTCCGGTACAACCTTCAACTA-3' | 52.17 | 62.19 |

**Table S4. The number of high-quality cells from each sample in the single-cell transcriptome analysis.**

**Table S5. MYH9 shRNA and NC shRNA sequences**

| **Lentivirus** | **Sequence** |
| --- | --- |
| LV3 (H1/GFP&Puro)-MYH9 Homo 938 (shRNA1) | 5'-GCAAATTCATTCGCATCAA-3' |
| LV3 (H1/GFP&Puro)-MYH9 Homo 1025 (shRNA 2) | 5'-CCATGAGGATTATGGGCAT-3' |
| LV3 (H1/GFP&Puro)-MYH9 Homo 1365 (shRNA 3) | 5'-GGGTATCAATGTGACCGAT-3' |
| LV3 (H1/GFP&Puro)-MYH9 Homo1434 (shRNA 4) | 5'-CCAGAAGGCGCAGACTAAA-3' |
| LV3 (H1/GFP&Puro)-NC (control) | 5’-TTCTCCGAACGTGTCACGTTTC-3’ |

**Table S6. TALEN plasmids and PCR primers**

| **TALEN Resource** | **Sequence** | **Note** | |
| --- | --- | --- | --- |
| TALEN vectors^#^ |  |  |  |
| TALEN-MYH9-L1 | 5'-CATCAATCCTTACAAG-3' | N/A |  |
| TALEN-MYH9-L2 | 5'-GGTCATCAATCCTTACA-3' | N/A |  |
| TALEN-MYH9-R1 | 5'-CCACAATCTCTTCAGAGT-3' | N/A |  |
| TALEN-MYH9-R2 | 5'-TCCACAATCTCTTCAGA-3' | N/A |  |
| TALEN-MYH9-R3 | 5'-TTCCACAATCTCTTCA-3' | N/A |  |
| TALEN PCR primers* | Primer sequences | GC (%) | Tm (°C) |
| 747-14s202-site3-u268-1s | 5'-GGGAGGATCTTCACTTACAGGGCAG | 56.00 | 64.40 |
| 748-14s202-site3-u239-2s | 5'-AGGATGCCGGTGCTTCACACC-3' | 61.90 | 65.30 |
| 749-14s202-site3-d294-1a | 5'-AACATGTCCTCTGGATAGCCACTGG-3' | 52.00 | 64.14 |
| 750-14s202-site3-d248-2a | 5'-CTACAGACCTCCATGATGCACTGCC-3' | 56.00 | 65.14 |

^#^TALEN vectors of left and right arms, TALEN-MYH9-L and TALEN-MYH9-R; *****PCR primers were used for the amplification and DNA sequencing of the products.

**Table S7. Sublines of GC cells with *MYH9* knocked down using TALEN technology.**

**Table S8. DNA probes used to target the *CTNNB1* promoter sequences**

| **DNA Probe** | **Targeted DNA Sequence** |
| --- | --- |
| O_1_ | 5'-GTGGCGGCAGGATACAGCGGCTTCTGCGCG-3' |
| O_2_ | 5'-AGCGGCTTCTGCGCGACTTATAAGAGCTCC-3' |
| O_3_ | 5'-ACTTATAAGAGCTCCTTGTGCGGCGCCATT-3' |
| O_4_ | 5'-TTGTGCGGCGCCATTTTAAGCCTCTCGGTC-3' |
| O_5_ | 5'-TTAAGCCTCTCGGTCTGTGGCAGCAGCGTTG-3' |
| O_3_L | 5'-ACTTATAAGAGCTCC-3' |
| O_3_M | 5'-GAGCTCCTTGTGCGG-3' |
| O_3_R | 5'-TTGTGCGGCGCCATT-3' |

**Table S9. Docking of the potential MYH9 DNA binding domains with the core *CTNNB1* promoter**

| **DBDs** | **Potential DBDs** | **Docking** | | **Binding energy** |
| --- | --- | --- | --- | --- |
|  |  | **Amino acid of MYH9 protein^#^** | **Nucleic acid of CTNNB1 promoter*** |  |
| DBD1 | 5'-LEEEQIILEDQNCKLAKEKKLL-3' | Asp 985 | C +10 | -24.7498 kJ/mol |
|  |  | Lys 1020 | C +10 |  |
| DBD2 | 5'-LEKTRRKLEGDSTDLSDQIAEL-3' | Glu 927 | T +8 | -120.947 kJ/mol |
|  |  | Arg 1050 | C +7 |  |
|  |  | Lys 992 | C +7 |  |
|  |  | Glu 993 | C +7 |  |
| DBD3 | 5'-LEKAKQTLENERGELANEVKVL-3' | Arg 1268 | G +4 | -781.88 kJ/mol |
|  |  | Lys 1274 | G +4 |  |
|  |  | Arg 1268 | A +5 |  |
|  |  | Lys 1217 | A +5 |  |
|  |  | Lys 1219 | C +7 |  |
|  |  | Lys 1352 | T +12 |  |
|  |  | Lys 1145 | T +14 |  |

^#^Numbering indicates the localization in MYH9 protein, while *numbering starts from the transcriptional start site (TSS) of *CTNNB1*.

**Table S10. Plasmids carrying *MYH9* mutation in the DBDs and key MYH9 phosphorylation sites**

| **Mutated Plasmids** | **Mutation site in *MYH9*** | | **Relative site in wild type *MYH9*** | |
| --- | --- | --- | --- | --- |
|  | **Amino acid of MYH9^#^** | **Nucleic acid of *MYH9**** | **Amino acid of MYH9^#^** | **Nucleic acid of *MYH9**** |
| DBD1M | Gly 985 | GGA  (2597 to 2599) | Asp 985 | GAC  (2597 to 2599) |
|  | Gly 1020 | GGA  (3058 to 3060) | Lys 1020 | AAG  (3058 to 3060) |
| DBD2M | Gly 927 | GGA  (2779 to 2781) | Glu 927 | GAG  (2779 to 2781) |
|  | Gly 1050 | GGA  (3148 to 3150) | Arg 1050 | CGC  (3148 to 3150) |
|  | Gly 992 | GGA  (2974 to 2976) | Lys 992 | AAG  (2974 to 2976) |
|  | Gly 993 | GGA  (2977 to 2979) | Glu 993 | GAA  (2977 to 2979) |
| DBD3M | Gly 1268 | GGA  (3802 to 3804) | Arg 1268 | CGC  (3802 to 3804) |
|  | Gly 1274 | GGA  (3820 to 3822) | Lys 1274 | AAG  (3820 to 3822) |
|  | Gly 1268 | GGA  (3802 to 3804) | Arg 1268 | CGC  (3802 to 3804) |
|  | Gly 1217 | GGA  (3649 to 3651) | Lys 1217 | AAG  (3649 to 3651) |
|  | Gly 1219 | GGA  (3655 to 3657) | Lys 1219 | AAG  (3655 to 3657) |
|  | Gly 1352 | GGA  (4054 to 4056) | Lys 1352 | AAG  (4054 to 4056) |
|  | Gly 1145 | GGA  (3433 to 3435) | Lys 1145 | AAA  (3433 to 3435) |
| G1800 | Gly 1800 | GGA  (5398 to 5400) | Thr 1800 | ACT  (5398 to 5400) |
| G1803 | Gly 1803 | GGA  (5407 to 5409) | Ser 1803 | TCC  (5407 to 5409) |
| G1808 | Gly 1808 | GGA  (5422 to 5424) | Ser 1808 | TCC  (5422 to 5424) |
| G1916 | Gly 1916 | GGA  (5746 to 5748) | Ser 1916 | TCC  (5746 to 5748) |
| G1943 | Gly 1943 | GGA  (5827 to 5829) | Ser 1943 | TCC  (5827 to 5829) |

^#^Numbering indicates the localization in MYH9 protein, while *number starts with the open reading frame sequence of *MYH9*.

**Table S11. Plasmids carrying *MYH9* mutations (M1–4) in the four putative NLSs**

| **Plasmids** | **Mutated sequence** | **Wild type sequence** | **Base position** | **Putative NLS** |
| --- | --- | --- | --- | --- |
| NLS1M (M'1) | **5'-**GCCGCCGCCCAC-3' | 5'-AAGAAGAGGCAC-3' | 421 bp to 432 bp | 5'-KKRH-3' |
| NLS2M (M'2) | 5'-GCGGCCGCCGCC-3' | 5'-CACAAGCGCAAG-3' | 3733 bp to 3744 bp | 5'-HKRK-3' |
| NLS3M (M'3) | 5'-GCCGCCGCCGCC-3' | 5'-AAGCGCAAGAAA-3' | 3736 bp to 3747 bp | 5'-KRKK-3' |
| NLS4M (M'4) | 5'-GCCGCCGAGGAGGCCCAGGCCCAGGAGCTGGAGGCCACCGCCGCCGCCCTG-3' | 5'-CGCAGGGAGGAGAAGCAGCGACAGGAGCTGGAGAAGACCCGCCGGAAGCTG-3' | 3109 bp to 3159 bp | 5'-RREEKQRQELEKTRRKL-3' |

**Table S12. Cell percentage with nuclear MYH9 expression after transfection with MYH9 mutation plasmids as analyzed using immunofluorescence and confocal microscopy of the MYH9-GFP protein**

| **Plasmids** | **Total cell number** | **Number of cells without nuclear MYH9-GFP** | **Number of cells with nuclear MYH9-GFP** | **Ratio of cells with nuclear MYH9-GFP (%)** |
| --- | --- | --- | --- | --- |
| Vector | 60 | 60 | 0 | 0 |
| Blank | 60 | 60 | 0 | 0 |
| WT | 60 | 0 | 60 | 100 |
| NLS1M (M'1) | 60 | 0 | 60 | 100 |
| NLS2M (M'2) | 60 | 0 | 60 | 100 |
| NLS3M (M'3) | 60 | 5 | 55 | 91.67 |
| NLS4M (M'4) | 60 | 3 | 57 | 95 |
| NLS1M+2M (M'5) | 60 | 2 | 58 | 96.67 |
| NLS1M+3M (M'6) | 60 | 5 | 55 | 91.67 |
| NLS1M+4M (M'7) | 60 | 4 | 56 | 93.33 |
| NLS2M+3M (M'8) | 60 | 4 | 56 | 93.33 |
| NLS2M+4M (M'9) | 60 | 6 | 54 | 90 |
| NLS3M+4M (M'10) | 60 | 25 | 35 | 58.33 |
| NLS1M+2M+3M (M'11) | 60 | 4 | 56 | 93.33 |
| NLS1M+2M+4M (M'12) | 60 | 5 | 55 | 91.67 |
| NLS1M+3M+4M (M'13) | 60 | 60 | 0 | 0 |
| NLS2M+3M+4M (M'14) | 60 | 59 | 1 | 1.67 |
| NLS1M+2M+3M+4M (M'15) | 60 | 60 | 0 | 0 |

**Table S13. PCR primers for transgenic mouse models**

| **Mice** | **Name of PCR primer** | **DNA sequences** |
| --- | --- | --- |
| Atp4b-cre | A1.0-cre-F | 5'-GCAGATAGCAAGCAAGCTCCAACC-3' |
|  | A1.0-cre-R | 5'-GGATTAACATTCTCCCACCGTCAG-3' |
| Myh9^fl/fl^ | Myh9_F1 | 5'-CTGGCTGGAATCTGAGTTGTGATTG-3' |
|  | Myh9_R1 | 5'-TTCTCGAGGACGGCATTCAAATC-3' |
|  | Myh9_F2 | 5'-CCTTGGCTATAGAACGGACATGTT-3' |
|  | Myh9_R2 | 5'-CACTGTGATGGGACGGAGAACAT-3' |
|  | Myh9_F3 | 5'-CGAATTCCGAAGTTCCTATTCTCTAG-3' |
|  | Myh9_R3 | 5'-GCTATCACTCTTGAGCCCATCCCT-3' |
|  | Cre Primer F | 5'-GAACGCACTGATTTCGACCA-3' |
|  | Cre Primer R | 5'-GCTAACCAGCGTTTTCGTTC-3' |
| LSL-hMYH9 | Transgene PCR primer_F (9TF) | 5'-CCCCATCAAGCTGATCCGGA-3' |
|  | Transgene PCR primer_R (9TR) | 5'-GGAGAACTTGGGCGGGTTCAT-3' |
|  | Internal control PCR primer_F (9IF) | 5'-TCAAAGCTGTCATTCCAGTGCTGC-3' |
|  | Internal control PCR primer_R (9IR) | 5'-GGATCAGGGCCAGGTACATTTGAT-3' |
|  | C1392_Cre_F1 | 5'-CCGGCGGCTCTAGAGCCTCTGCT-3' |
|  | C1392_Cre_R1 | 5'-CAGCTTCTTGGCAGCCCAGTCGG-3' |
|  | C1392_Cre_F2 | 5'-TAGAACTAGTGGATCCGGAACCC-3' |
|  | C1392_Cre_R2 | 5'-AGCTCTGCCATGTCCTCCACCTT-3' |
| Tff1 KO | Tff1_45466_F | 5'-CCTAGAGGCAACCCCTGATG-3' |
|  | Tff1_45466_R | 5'-TGTGTGCTGGCTAGGTGGAG-3' |

**Table S14. STS dose escalation data in nude mice and C57BL/6 mice**

| **Group** | | **Daily drug dose (mg/kg/day)** | | **Days per course** | | **Treatment course** | | **Survival rate**  **(n/N)** | | **Change in body weight**  **(mean ± SEM)** | |
| --- | --- | --- | --- | --- | --- | --- | --- | --- | --- | --- | --- |
| **Nude mice** | | | | | | | | | | |  |
| A | 0.4 | | 5 | | 1 | | 5/5 | | 0.02 ± 0.46 | |  |
| B | 0.6 | | 5 | | 1 | | 5/5 | | -0.34 ± 0.70 | |  |
| C | 0.8 | | 5 | | 1 | | 2/5 | | -2.96 ± 0.46 | |  |
| D | 1 | | 5 | | 1 | | 0/5 | | -3.74 ± 0.26 | |  |
| **C57BL/6 mice (one week)** | | | | | | | | | | |  |
| A | 0.4 | | 5 | | 1 | | 3/5 | | 0.03 ± 0.38 | |  |
| B | 0.6 | | 5 | | 1 | | 5/5 | | 0.07 ± 0.45 | |  |
| C | 0.8 | | 5 | | 1 | | 5/5 | | -1.45 ± 0.38 | |  |
| **C57BL/6 mice (14 week)** | | | | | | | | | | |  |
| A | 0.6 | | 5 | | 14 | | 2/5 | | 11.04 ± 2.78 | |  |
| B | 0.6 | | 5 | | 7 | | 5/5 | | 32.12 ± 0.21 | |  |
| C | 0.6 | | 5 | | 5 | | 5/5 | | 35.60 ± 0.33 | |  |
| N, number of mice per group in the experiment; n, number of survivors per group at the end of the experiment. | | | | | | | | | | |  |

**References**

1. Muhammad Aslam MK, Kumaresan A, Rajak SK, Tajmul M, Datta TK, Mohanty TK, et al. Comparative proteomic analysis of Taurine, Indicine, and crossbred (Bos taurus x Bos indicus) bull spermatozoa for identification of proteins related to sperm malfunctions and subfertility in crossbred bulls. Theriogenology. 2015; 84: 624-33.

2. Biasini M, Bienert S, Waterhouse A, Arnold K, Studer G, Schmidt T, et al. SWISS-MODEL: modelling protein tertiary and quaternary structure using evolutionary information. Nucleic Acids Res. 2014; 42: W252-8.

3. Ye G, Huang K, Yu J, Zhao L, Zhu X, Yang Q, et al. MicroRNA-647 Targets SRF-MYH9 Axis to Suppress Invasion and Metastasis of Gastric Cancer. Theranostics. 2017; 7: 3338-53.

4. Hockemeyer D, Wang HY, Kiani S, Lai CS, Gao Q, Cassady JP, et al. Genetic engineering of human pluripotent cells using TALE nucleases. Nat Biotechnol. 2011; 29: 731-4.

5. Liang H, He SM, Yang JY, Jia XY, Wang P, Chen X, et al. PTEN alpha, a PTEN Isoform Translated through Alternative Initiation, Regulates Mitochondrial Function and Energy Metabolism. Cell Metab. 2014; 19: 836-48.

6. Hozak P, Jackson DA, Cook PR. Replication factories and nuclear bodies: the ultrastructural characterization of replication sites during the cell cycle. J Cell Sci. 1994; 107 ( Pt 8): 2191-202.

7. Hofmann WA, Stojiljkovic L, Fuchsova B, Vargas GM, Mavrommatis E, Philimonenko V, et al. Actin is part of pre-initiation complexes and is necessary for transcription by RNA polymerase II. Nat Cell Biol. 2004; 6: 1094-101.

8. Li Q, Sarna SK. Nuclear myosin II regulates the assembly of preinitiation complex for ICAM-1 gene transcription. Gastroenterology. 2009; 137: 1051-60, 60 e1-3.

9. Zhang N, Wei P, Gong A, Chiu WT, Lee HT, Colman H, et al. FoxM1 promotes beta-catenin nuclear localization and controls Wnt target-gene expression and glioma tumorigenesis. Cancer Cell. 2011; 20: 427-42.

10. Tang WL, Chen WC, Roy A, Undzys E, Li SD. A Simple and Improved Active Loading Method to Efficiently Encapsulate Staurosporine into Lipid-Based Nanoparticles for Enhanced Therapy of Multidrug Resistant Cancer. Pharm Res. 2016; 33: 1104-14.

11. Shimada S, Mimata A, Sekine M, Mogushi K, Akiyama Y, Fukamachi H, et al. Synergistic tumour suppressor activity of E-cadherin and p53 in a conditional mouse model for metastatic diffuse-type gastric cancer. Gut. 2012; 61: 344-53.

12. Syder AJ, Karam SM, Mills JC, Ippolito JE, Ansari HR, Farook V, et al. A transgenic mouse model of metastatic carcinoma involving transdifferentiation of a gastric epithelial lineage progenitor to a neuroendocrine phenotype. Proc Natl Acad Sci U S A. 2004; 101: 4471-6.

13. Zhao Z, Hou N, Sun Y, Teng Y, Yang X. Atp4b promoter directs the expression of Cre recombinase in gastric parietal cells of transgenic mice. J Genet Genomics. 2010; 37: 647-52.

14. Conti MA, Even-Ram S, Liu C, Yamada KM, Adelstein RS. Defects in cell adhesion and the visceral endoderm following ablation of nonmuscle myosin heavy chain II-A in mice. J Biol Chem. 2004; 279: 41263-6.

15. Vicente-Manzanares M, Ma X, Adelstein RS, Horwitz AR. Non-muscle myosin II takes centre stage in cell adhesion and migration. Nat Rev Mol Cell Biol. 2009; 10: 778-90.

16. Yanagihara N, Tachikawa E, Izumi F, Yasugawa S, Yamamoto H, Miyamoto E. Staurosporine: an effective inhibitor for Ca2+/calmodulin-dependent protein kinase II. J Neurochem. 1991; 56: 294-8.
